# Cu(II) and Zn(II) Complexes of New 8-Hydroxyquinoline Schiff Bases: Investigating Their Structure, Solution Speciation, and Anticancer Potential

**DOI:** 10.1021/acs.inorgchem.3c01066

**Published:** 2023-07-13

**Authors:** Leonor Côrte-Real, Vivien Pósa, Matilde Martins, Raquel Colucas, Nóra V. May, Xavier Fontrodona, Isabel Romero, Filipa Mendes, Catarina Pinto Reis, Maria Manuela Gaspar, João Costa Pessoa, Éva A. Enyedy, Isabel Correia

**Affiliations:** † Centro de Química Estrutural, Institute of Molecular Sciences, and Department of Chemical Engineering, Instituto Superior Técnico, Universidade de Lisboa, Avenida Rovisco Pais 1, 1049-001 Lisboa, Portugal; ‡ MTA-SZTE Lendület Functional Metal Complexes Research Group, Department of Inorganic and Analytical Chemistry, Interdisciplinary Excellence Centre, University of Szeged, Dóm tér 7, H-6720 Szeged, Hungary; § Centre for Structural Science, Research Centre for Natural Sciences, Eötvös Loránd Research Network, Magyar Tudósok krt. 2, H-1117 Budapest, Hungary; ∥ Departament de Química and Serveis Tècnics de Recerca, Universitat de Girona, Campus de Montilivi, E-17071 Girona, Spain; ⊥ Centro de Ciências e Tecnologias Nucleares and Department of Nuclear Sciences and Engineering, Instituto Superior Técnico, Universidade de Lisboa, Estrada Nacional 10 (km139,7), 2695-066 Bobadela LRS, Portugal; # Research Institute for Medicines (iMed.ULisboa), Faculty of Pharmacy, Universidade de Lisboa, 1649-003 Lisboa, Portugal; g Instituto de Biofísica e Engenharia Biomédica, Faculdade de Ciências, Universidade de Lisboa, 1749-016 Lisboa, Portugal

## Abstract

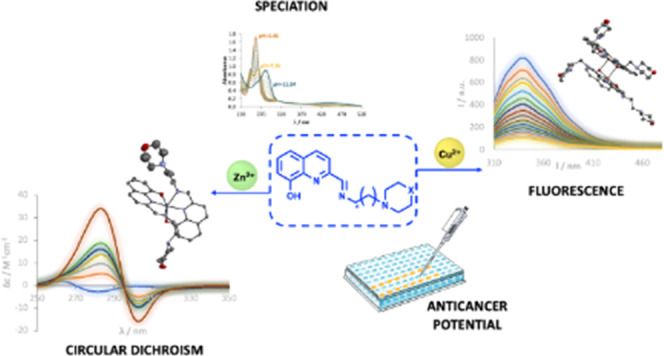

We report the synthesis and characterization of three novel Schiff bases (**L1**–**L3**) derived from the condensation of 2-carbaldehyde-8-hydroxyquinoline with amines containing morpholine or piperidine moieties. These were reacted with CuCl_2_ and ZnCl_2_ yielding six new coordination compounds, with the general formula ML_2_, where M = Cu(II) or Zn(II) and L = **L1**–**L3**, which were all characterized by analytical, spectroscopic (Fourier transform infrared (FTIR), UV–visible absorption, nuclear magnetic resonance (NMR), or electron paramagnetic resonance (EPR)), and mass spectrometric techniques, as well as by single-crystal X-ray diffraction. In the solid state, two Cu(II) complexes, with **L1** and **L2**, are obtained as dinuclear compounds, with relatively short Cu–Cu distances (3.146 and 3.171 Å for **Cu**_**2**_**(L1)**_**4**_ and **Cu**_**2**_**(L2)**_**4**_, respectively). The free ligands show moderate lipophilicity, while their complexes are more lipophilic. The p*K*_a_ values of **L1**–**L3** and formation constants of the complex (for ML and ML_2_) species were determined by spectrophotometric titrations, with the Cu(II) complexes showing higher stability than the Zn(II) complexes. EPR indicated the presence of several species in solution as pH varied and binding modes were proposed. The binding of the complexes to bovine serum albumin (BSA) was evaluated by fluorescence and circular dichroism (CD) spectroscopies. All complexes bind BSA, and as demonstrated by CD, the process takes several hours to reach equilibrium. The antiproliferative activity was evaluated in malignant melanoma cells (A375) and in noncancerous keratinocytes (HaCaT). All complexes display significant cytotoxicity (IC_50_ < 10 μM) but modest selectivity. The complexes show higher activity than the free ligands, the Cu(II) complexes being more active than the Zn(II) complexes, and approximately twice more cytotoxic than cisplatin. A Guava ViaCount assay corroborated the antiproliferative activity.

## Abstract 2

Six new Cu(II) and Zn(II) 8-hydroxyquinoline Schiff bases containing piperidine or morpholine moieties are synthesized and structurally characterized in the solid state and solution. The Cu(II) complexes show higher stability than the Zn(II) complexes, as well as higher cytotoxic activity against malignant melanoma.

## 1 Introduction

Cancer represents a huge public health and economic problem, and its burden is expected to increase in the next decades. In 2020, nearly 10 million people died of cancer worldwide.^1^ The global cancer incidence is expected to be 28.4 million cases in 2040, a 47% increase from 2020.^2^ Thus, the development of new treatment strategies and of new chemotherapeutic drugs is of utmost importance.

Malignant melanoma develops in melanocytes, and despite being less common than other types of skin cancers, it is the most aggressive due to its high ability to spread rapidly to other organs if not treated at an early stage. Current treatments involve surgery and immunotherapy, and for metastatic diseases, chemotherapeutic alkylating drugs such as dacarbazine and temozolomide have been used alone or in combination with other drugs (e.g., cisplatin). Despite the increased overall survival rate, many malignant melanoma patients develop treatment resistance or experience relapse; thus, new treatment options are urgently needed.^2,3^

Nitrogen heterocycles are important constituents of pharmaceutical drugs, such as dacarbazine and temozolomide. A study by Njardarson,^4^ which analyzed the frequency and structural variety of *N*-heterocycles in FDA-approved small molecule drugs, showed that they are featured in more than 59% of drugs. Among these compounds, the most prevalent nitrogen ring is piperidine, followed by pyridine, with morpholine also being highly frequent. These 6-membered *N*-heterocycles present a wide range of therapeutic effects such as antioxidant, anti-inflammatory, antimicrobial, anticancer, antidiabetic, antituberculosis, and antidepressant action, among many others. Several reviews have been published in recent years highlighting their therapeutic value.^5−7^

8-Hydroxyquinolines are also considered a privileged structure in medicinal chemistry. They are constituted by two fused aromatic rings, one pyridine and one phenol, and are suitable chelators to metal ions through the formation of (N,O) 5-membered chelate rings. They also show a wide range of biological activities and are important ionophores, able to transport endogenous metal ions through cellular membranes.^8^

In our ongoing search for new anticancer drugs, we have developed different derivatives of 8-hydroxyquinolines (8HQ) and complexed them to different metal ions to take advantage of the synergistic effect of combining a bioactive organic moiety and biologically significant metals, such as copper, zinc, vanadium, iron, nickel, and ruthenium.^9−12^ Coordination compounds present several advantages when compared to organic molecules since they may influence the size, charge, lipophilicity, and interaction with biomolecules, showing different coordination numbers and geometries and may also present redox activity. Our strategy explored the increase in the coordination ability of 8HQ by introducing substituents in the *ortho* position to the quinoline N atom while also containing donor groups in suitable positions to participate in the chelation of the metal ion. Condensation with different nitrogen-containing molecules yielded different types of ligands, and overall, the compounds showed cytotoxic activity in different cancer cell lines such as malignant melanoma, lung, and colon.^9,10,12^ The presence of two bioactive moieties, 8HQ and benzothiazole (another *N*-heterocycle), yielded a Schiff base with interesting anticancer properties.^10,12^ Its Zn(II) complex showed the ability to reduce the tumor volume in an *in vivo* study with a syngeneic colon cancer mouse model upon incorporation in a liposomal formulation to increase drug aqueous solubility and targeting efficacy.^12^

A family of benzohydrazones derived from 8HQ-2-carbaldehyde and benzylhydrazides substituted in the *para* position was also recently developed by us and complexed to oxidovanadium(IV)^9^ and copper(II).^13^ Their solution behavior was evaluated, and they were screened in malignant melanoma (A375) and lung (A549) cancer cells, showing IC_50_ values in the low micromolar concentration range. Overall, the metal complexes exhibited the ability to induce the generation of reactive oxygen species (ROS) and double-stranded breaks in both cancer cell lines, as well as apoptosis.

Another example, a work from Enyedy and co-workers,^14^ showed cytotoxic activity (IC_50_ values 0.20–3.27 μM) of 8-hydroxyquinoline-derived Mannich bases linked to morpholine, piperidine, and other types of substituents in the drug-resistant human uterine sarcoma MES-SA/Dx5 cell line. Moreover, these compounds were also tested in primary hepatocytes, revealing a much better selectivity profile than the common chemotherapeutic agent doxorubicin. The work from Gable and co-workers^15^ also highlighted the potential of morpholine-type Schiff base complexes in cancer research. They prepared complexes with several metal ions, with the general structure [ML]^n+^ (M = Zn(II), Cd(II), Mn(II), Cu(II), Ni(II), Ag(I), Fe(III), and Co(II), *n* = 0, 1, 2) bearing a new morpholine-based ligand (L) as a product of condensation between 3-morpholinopropylamine and salicylaldehyde. Results revealed that Zn(II), Cd(II), Mn(II), Ni(II), and Ag(I) complexes possess different anticancer potentials in MCF-7, MDA-MB-231, and PC-3 cancer cells. More importantly, it was observed that Zn(II), Mn(II), and Ni(II) compounds demonstrated weaker cytotoxic activity in the WI-38 normal cell line, meaning that these compounds have good selectivity behavior toward cancer cells.

**Scheme 1:**
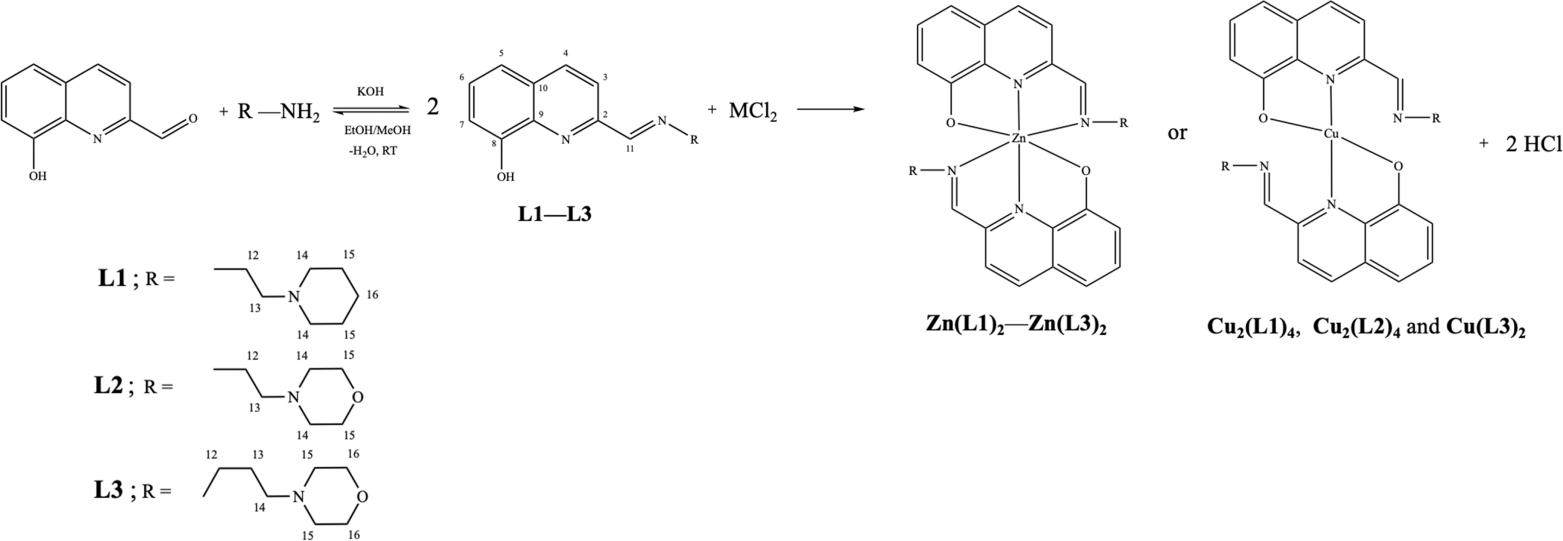
– Outline of the Synthesis of the New Schiff Bases, **L1**–**L3** and of Metal Complexes **Zn(L1)**_**2**_–**Zn(L3)**_**2**_ and **Cu**_**2**_**(L1)**_**4**_, **Cu**_**2**_**(L2)**_**4**_, and **Cu(L3)**_**2**_. The Atoms Are Numbered for NMR Assignment

The results obtained with these types of ligands inspired us to develop a new ligand family in which we aim to take advantage of the synergistic effect of combining 8HQ and morpholine or piperidine heterocycles in the same molecule. Three molecules were obtained by the condensation of 8HQ-2-carbaldehyde with amines containing morpholine or piperidine moieties, and different length spacers, that were reacted with Cu(II) and Zn(II) salts to yield six new coordination compounds (see Scheme 1) characterized by analytic and spectroscopic techniques, as well as by single-crystal X-ray diffraction. Their solution behavior was studied by UV–visible (UV–vis) spectrophotometry and corroborated by electron paramagnetic resonance (EPR) or nuclear magnetic resonance (NMR), which allowed the proposal of their binding modes in solution and particularly at physiological pH. A preliminary biological study is presented in which the compounds were screened in malignant melanoma cells, as well as a noncancerous cell line, to evaluate their selectivity.

## 2 Experimental Section

All usual laboratory chemicals were acquired from commercial suppliers and used without additional purification. Synthesized compounds were characterized by C, H, and N elemental analyses and by UV–vis, Fourier transform infrared (FTIR), ^1^H/^13^C NMR, and EPR spectroscopies (whenever appropriate).

### 2.1 Materials

8-Hydroxy-2-quinolinecarboxaldehyde, 1-(2-aminoethyl)-piperidine, 4-(2-aminoethyl)morpholine, and 3-morpholinopropylamine were purchased from Sigma-Aldrich. 4-(2-Hydroxy ethyl)-1-piperazine ethanesulfonic acid (HEPES) was obtained from Sigma-Aldrich, and KCl, KOH, HCl, ethylenediamine tetraacetic acid (EDTA), and potassium hydrogen phthalate were obtained from Reanal (Hungary). All products were used without further purification. ZnCl_2_ and CuCl_2_ were purchased from Thermo Scientific and BDH Chemicals, respectively. CuCl_2_ and ZnCl_2_ stock solutions were prepared by the dissolution of anhydrous CuCl_2_ and ZnCl_2_ in water; their concentrations were determined by complexometry with EDTA. The water used in all biological studies was double-deionized in a Milli-Q system (Millipore). All of the remaining chemicals used were of analytical grade.

### 2.2 Apparatus

^1^H and ^13^C NMR spectra were recorded at ambient temperature on a Bruker Avance +400 MHz spectrometer. ^1^H and ^13^C chemical shifts (δ) are expressed in ppm relative to Me_4_Si or to the deuterated solvent residual peak. For the compound’s characterization, electronic UV–vis absorption spectra were recorded with a PerkinElmer Lambda 35 spectrophotometer. Infrared spectra (IR, 4000–400 cm^–1^) were recorded on a Jasco FT/IR 4100 spectrophotometer in KBr pellets. Elemental analysis for C, H, and N, was carried out on a FISONS EA 1108 CHNS-O apparatus at the *Laboratório de Análises* of *Instituto Superior Técnico*. An LCQ FleetTM ion trap mass spectrometer from Thermo Scientific was used to measure ESI-MS spectra of methanolic solutions of the compounds in both the positive and negative ion modes. Circular dichroism (CD) spectra were recorded at 25 °C on a JASCO 720 spectropolarimeter with quartz Suprasil cells (10 mm optical path), using the 260–900 nm range and a UV–vis photomultiplier as a detector. Fluorescence measurements were carried out on a spectrofluorometer PerkinElmer LS 55 equipped with a xenon lamp and in quartz cuvettes with 10 mm optical path; these experiments were carried out at room temperature and were all steady-state measurements.

Single-crystal X-ray diffraction data were measured at 100 K on a Bruker D8 Quest Eco three-circle diffractometer equipped with a ceramic X-ray tube (Mo Kα, λ = 0.71073 Å) and a doubly curved silicon crystal Triumph monochromator.

### 2.3 Synthesis and Characterization

#### 2.3.1 Synthesis of the Ligand Precursors, L1–L3

To an ethanolic (**L1**) or methanolic (**L2**, **L3**) solution of the selected amine (1 mmol) and KOH (1 mmol), 8-hydroxy-2-quinolinecarboxaldehyde (1 mmol) was added. The resulting orange solution was stirred for 1 h at room temperature. The solvent was then evaporated, and the oily residue was dissolved in dichloromethane, filtered, and recrystallized with diethyl ether (**L1**) or *n*-hexane (**L2** and **L3**).

**L1**: Yield: 91%. Elemental analyses calcd for C_17_H_20_KN_3_O·0.1H_2_O (323.26 g/mol) (%): C: 63.16, H: 6.30, N: 13.00. Found (%): C: 63.4, H: 6.4, N: 12.8. Mass spectrometry MS (ESI^+^): *m*/*z* (calcd): 322.13, found: 322.12 [M + K^+^]. ^1^H NMR [DMSO-*d*_6_, Me_4_Si, δ/ppm]: 8.49 [s, 1, H_11_]; 8.10 [d, 1, ^3^*J*_HH_ = 8, H_4_]; 7.84 [d, 1, ^3^*J*_HH_ = 8, H_3_]; 7.26 [t, 1, ^3^*J*_HH_ = 8, H_6_]; 6.88 [d, 1, ^3^*J*_HH_ = 8, H_5_]; 6.84 [d, 2, ^3^*J*_HH_ = 8, H_7_]; 3.73* [m, 2, H_12_]; 2.55 [t, 2, ^3^*J*_HH_ = 8, H_13_]; 2.39 [m, 4, H_14_], 1.47 [m, 6, H_15_+H_16_]. ^13^C NMR [DMSO-*d*_6_, Me_4_Si, δ/ppm]: 163.40 (C_11_); 162.32 (C_8_); 150.05 (C_2_); 141.34 (C_9_); 136.03 (C_4_); 130.54 (C_10_); 129.50 (C_6_); 117.38 (C_3_); 113.31 (C_7_); 110.64 (C_5_); 59.25 (C_13_); 58.09 (C_12_); 54.28 (C_14_); 25.63 (C_15_+C_16_). *Under the water (solvent) peak. UV–vis: [DMSO, λ_max_/nm (ε/M^–1^ cm^–1^)]: 270 (18362), 314 (sh), 347 (2471). FTIR [KBr, cm^–1^]: 3044 (ν_C–H_ aromatic); 2933 (ν_C–H_ aliphatic); 1648 (ν_C=N_); 1541 and 1447 (ν_C=C_ aromatic); 1092 (ν_C–O_).

**L2**: Yield: 80%. Elemental analyses calcd for C_16_H_18_KN_3_O_2_·1.2 H_2_O (345.05 g/mol) (%): C: 55.69, H: 5.96, N: 12.18. Found (%): C: 55.6, H: 5.9, N: 11.9. Mass spectrometry MS (ESI^+^): *m*/*z* (calcd): 324.1, found: 324.03 [M + K^+^]. ^1^H NMR [DMSO-*d*_6_, Me_4_Si, δ/ppm]: 8.46 [s, 1, H_11_]; 8.10 [d, 1, ^3^*J*_HH_ = 12, H_4_]; 7.83 [d, 1, ^3^*J*_HH_ = 8, H_3_]; 7.26 [t, 1, ^3^*J*_HH_ = 8, H_6_]; 6.86 [m, 2, H_5_+H_7_]; 3.73 [m, 2, H_12_]; 3.55 [m, 4, H_15_]; 2.59 [t, 2, ^3^*J*_HH_ = 8, H_13_]; 2.42 [m, 4, H_14_]. ^13^C NMR [DMSO-*d*_6_, Me_4_Si, δ/ppm]: 163.59 (C_11_); 162.90 (C_8_); 149.81 (C_2_); 142.06 (C_9_); 136.00 (C_4_); 130.64 (C_10_); 129.60 (C_6_); 117.37 (C_3_); 113.37 (C_7_); 110.11 (C_5_); 66.20 (C_15_); 58.85 (C_13_); 57.65 (C_12_); 53.54 (C_14_). UV–vis: [DMSO, λ_max_/nm (ε/M^–1^ cm^–1^)]: 269 (19282), 314 (sh), 349 (2040). FTIR [KBr, cm^–1^]: 2960 (ν_C–H_ aliphatic); 1649 (ν_C=N_); 1541 and 1448 (ν_C=C_ aromatic); 1115 (ν_C–O_).

**L3**: Yield: 72%. Elemental analyses calcd for C_17_H_20_KN_3_O_2_·2.0H_2_O (373.49 g/mol) (%): C: 54.67, H: 6.48, N: 11.25. Found (%): C: 54.7, H: 6.4, N: 11.2. Mass spectrometry MS (ESI^+^): *m*/*z* (calcd): 338.12, found: 338.05 [M + K^+^]. ^1^H NMR [DMSO-*d*_6_, Me_4_Si, δ/ppm]: 8.47 [s, 1, H_11_]; 8.05 [d, 1, ^3^*J*_HH_ = 8, H_4_]; 7.81 [d, 1, ^3^*J*_HH_ = 8, H_3_]; 7.22 [t, 1, ^3^*J*_HH_ = 8, H_6_]; 6.75 [t, 2, ^3^*J*_HH_ = 8, H_5_+H_7_]; 3.61* [m, 2, H_12_]; 3.56 [m, 4, H_16_]; 2.34 [m, 6, H_13_+H_15_]; 1.77 [t, 2, ^3^*J*_HH_ = 8, H_14_]. ^13^C NMR [DMSO-*d*_6_, Me_4_Si, δ/ppm]: 164.37 (C_8_); 163.06 (C_11_); 149.86 (C_2_); 142.12 (C_9_); 135.89 (C_4_); 130.86 (C_10_); 129.75 (C_6_); 116.62 (C_3_); 113.57 (C_7_); 107.99 (C_5_); 66.27 (C_16_); 58.48 (C_12_); 56.23 (C_13_); 53.44 (C_15_); 27.49 (C_14_). *Under the water (solvent) peak. UV–vis: [DMSO, λ_max_/nm (ε/M^–1^ cm^–1^)]: 269 (19592), 314 (sh), 349 (1929). FTIR [KBr, cm^–1^]: 2953 (ν_C–H_ aliphatic); 1649 (ν_C=N_); 1541 and 1434 (ν_C=C_ aromatic); 1115 (ν_C–O_).

#### 2.3.2 Synthesis of the Zn(II) Complexes, Zn(L1)_2_–Zn(L3)_2_

To an ethanolic solution of the adequate amine (2 mmol) and KOH (2 mmol), 8-hydroxy-2-quinolinecarboxaldehyde (2 mmol) was added. The resulting orange solution was stirred for 1 h at room temperature. ZnCl_2_ (1 mmol), was then added, and the resulting solution was stirred at room temperature for 1 h and subsequently the solvent was evaporated. The precipitate was dissolved in dichloromethane, filtered, and recrystallized with diethyl ether.

**Zn(L1)**_**2**_: Yield: 89%. Elemental analyses calcd for C_34_H_40_N_6_O_2_Zn·0.3 H_2_O (635.52 g/mol) (%): C: 64.26, H: 6.44, N: 13.22. Found (%): C: 64.3, H: 6.5, N: 13.1. Mass spectrometry MS (ESI^+^): *m*/*z* (calcd): 629.11, found: 629.26 [M + H^+^]. ^1^H NMR [DMSO-*d*_6_, Me_4_Si, δ/ppm]: 8.62 [d, 2, ^3^*J*_HH_ = 8, H_4_]; 8.59 [s, 2, H_11_]; 7.92 [d, 2, ^3^*J*_HH_ = 8, H_3_]; 7.46 [t, 2, ^3^*J*_HH_ = 8, H_6_]; 6.93 [d, 2, ^3^*J*_HH_ = 8, H_5_]; 6.58 [d, 2, ^3^*J*_HH_ = 8, H_7_]; 3.16 [t, 4, ^3^*J*_HH_ = 8, H_12_]; 1.77 [m, 8, H_14_]; 1.66 [t, 4, ^3^*J*_HH_ = 8, H_13_]; 1.64 [m, 12, H_15_+H_16_]. ^13^C NMR [DMSO-*d*_6_, Me_4_Si, δ/ppm]: 164.35 (C_8_); 158.78 (C_11_); 142.14 (C_2_); 139.89 (C_4_); 139.68 (C_9_); 132.61 (C_6_); 129.58 (C_10_); 121.00 (C_3_); 111.74 (C_7_); 106.98 (C_5_); 57.06 (C_13_); 55.12 (C_12_); 53.41 (C_14_); 25.36 (C_15_+C_16_); UV–vis: [DMSO, λ_max_/nm (ε/M^–1^ cm^–1^)]: 300 (46260); 342 (sh); 370 (sh); 497 (3200). UV–vis: [CH_2_Cl_2_, λ_max_/nm (ε/M^–1^ cm^–1^)]: 300 (29475), 374 (sh); 503 (2092). FTIR [KBr, cm^–1^]: 3058 (ν_C–H_ aromatic); 2932 (ν_C–H_ aliphatic); 1643 (ν_C=N_); 1593 and 1447 (ν_C=C_ aromatic); 1112 (ν_C–O_).

**Zn(L2)**_**2**_: Yield: 63%. Elemental analyses calcd for C_32_H_36_N_6_O_4_Zn·0.5H_2_O (643.02 g/mol) (%): C: 59.77, H: 5.80, N: 13.07. Found (%): C: 59.9, H: 5.8, N: 13.0. Mass spectrometry MS (ESI^+^): *m*/*z* (calcd): 633.22, found: 633.14 [M + H^+^]. ^1^H NMR [DMSO-*d*_6_, Me_4_Si, δ/ppm]: 8.62 [m, 4, H_4_+H_11_]; 7.92 [d, 2, ^3^*J*_HH_ = 8, H_3_]; 7.46 [t, 2, ^3^*J*_HH_ = 8, H_6_]; 6.94 [d, 2, ^3^*J*_HH_ = 8, H_5_]; 6.60 [d, 2, ^3^*J*_HH_ = 8, H_7_]; 3.33 [m, 8, H_15_]; 3.18 [t, 4, ^3^*J*_HH_ = 8, H_12_]; 1.81 [m, 8, H_14_]; 1.68 [t, 4, ^3^*J*_HH_ = 8, H_13_]. ^13^C NMR [DMSO-*d*_6_, Me_4_Si, δ/ppm]: 163.75 (C_8_); 159.07 (C_11_); 144.72 (C_2_); 139.94 (C_4_); 139.66 (C_9_); 132.62 (C_6_); 131.65 (C_10_); 121.43 (C_3_); 111.47 (C_7_); 107.12 (C_5_); 65.94 (C_15_); 56.67 (C_13_); 54.81 (C_12_); 52.69 (C_14_). UV–vis: [DMSO, λ_max_/nm (ε/M^–1^ cm^–1^)]: 301 (42486); 342 (sh); 370 (sh); 497 (2906). UV–vis: [CH_2_Cl_2_, λ_max_/nm (ε/M^–1^ cm^–1^)]: 301 (44646), 374 (sh); 504 (2934). FTIR [KBr, cm^–1^]: 3053 (ν_C–H_ aromatic); 2955 (ν_C–H_ aliphatic); 1641 (ν_C=N_); 1593 and 1451 (ν_C=C_ aromatic); 1113 (ν_C–O_).

**Zn(L3)**_**2**_: Yield: 51%. Elemental analyses calcd for C_34_H_40_N_6_O_4_Zn·0.2H_2_O (665.72 g/mol) (%): C: 61.34, H: 6.12, N: 12.62. Found (%): C: 61.3, H: 6.2, N: 12.7. Mass spectrometry MS (ESI^+^): *m*/*z* (calcd): 661.25, found: 661.03 [M + H^+^]. ^1^H NMR [DMSO-*d*_6_, Me_4_Si, δ/ppm]: 8.66 [s, 2 H_11_]; 8.60 [d, 2, ^3^*J*_HH_ = 8, H_4_]; 7.92 [d, 2, ^3^*J*_HH_ = 8, H_3_]; 7.44 [t, 2, ^3^*J*_HH_ = 8, H_6_]; 6.91 [d, 2, ^3^*J*_HH_ = 8, H_5_]; 6.57 [d, 2, ^3^*J*_HH_ = 8, H_7_]; 3.33* [m, 8, H_16_]; 3.19 [m, 4, H_12_]; 1.80 [m, 12, H_13_+H_15_], 0.95 [m, 4, H_14_]. ^13^C NMR [DMSO-*d*_6_, Me_4_Si, δ/ppm]: 164.28 (C_8_); 158.53 (C_11_); 142.33 (C_2_); 139.78 (C_4_); 139.57 (C_9_); 132.53 (C_6_); 131.13 (C_10_); 121.32 (C_3_); 111.78 (C_7_); 107.01 (C_5_); 66.01 (C_16_); 56.46 (C_12_); 55.19 (C_13_); 52.36 (C_15_); 25.88 (C_14_). *Under the solvent signal. UV–vis: [DMSO, λ_max_/nm (ε/M^–1^ cm^–1^)]: 300 (43292); 342 (sh); 370 (sh); 498 (3161); UV–vis: [CH_2_Cl_2_, λ_max_/nm (ε/M^–1^ cm^–1^)]: 301 (49769); 374 (sh); 504 (3102). FTIR [KBr, cm^–1^]: 3056 (ν_C–H_ aromatic); 2954 (ν_C–H_ aliphatic); 1639 (ν_C=N_); 1594 and 1447 (ν_C=C_ aromatic); 1112 (ν_C–O_).

#### 2.3.3 Synthesis of the Cu(II) Complexes

To a methanolic solution of the adequate amine (2 mmol) and KOH (2 mmol), 8-hydroxy-2-quinolinecarboxaldehyde (2 mmol) was added. The resulting orange solution was stirred for 1 h at room temperature. CuCl_2_ (1 mmol), was then added, and the resulting solution was stirred at room temperature for 1 h and subsequently the solvent was evaporated. The precipitate was dissolved in dichloromethane, filtered, and recrystallized with *n*-hexane.

**Cu**_**2**_**(L1)**_**4**_. Yield: 35%. Elemental analyses calcd for C_68_H_80_N_12_O_4_Cu_2_·0.5 H_2_O (1263.51 g/mol) (%): C: 64.54, H: 6.45, N: 13.28. Found (%): C: 64.4, H: 6.5, N: 13.2 Mass spectrometry MS (ESI^+^): *m*/*z* (calcd): 628.26, found: 627.79 [M + 2H^2+^]. UV–vis: [DMSO, λ_max_/nm (ε/M^–1^ cm^–1^)]: 268 (37291); 312 (sh); 422 (2383). [CH_2_Cl_2_, λ_max_/nm (ε/M^–1^ cm^–1^)]: 271 (41828); 309 (sh); 412 (5199). FTIR [KBr, cm^–1^]: 3050 (ν_C–H_ aromatic); 2934 (ν_C–H_ aliphatic); 1636 (ν_C=N_); 1558 and 1455 (ν_C=C_ aromatic); 1112 (ν_C–O_).

**Cu**_**2**_**(L2)**_**4**_. Yield: 56%. Elemental analyses calcd for C_64_H_72_N_12_O_8_Cu_2_·2.0 H_2_O (1298.44 g/mol) (%): C: 59.11, H: 5.89, N: 12.92. Found (%): C: 59.0, H: 5.7, N: 12.7. Mass spectrometry MS (ESI^+^): *m*/*z* (calcd): 632.22, found: 631.82 [M + 2H^2+^]. UV–vis: [DMSO, λ_max_/nm (ε/M^–1^ cm^–1^)]: 297 (53105); 308 (sh); 422 (3368). [CH_2_Cl_2_, λ_max_/nm (ε/M^–1^ cm^–1^)]: 268 (30435); 305 (sh); 423 (3278). FTIR [KBr, cm^–1^]: 3434 (br, H_2_O molecules); 2921 (ν_C–H_ aliphatic); 1632 (ν_C=N_); 1558 and 1448 (ν_C=C_ aromatic); 1112 (ν_C–O_).

**Cu(L3)**_**2**_. Yield: 57%. Elemental analyses calcd for C_34_H_40_N_6_O_4_Cu·0.5H_2_O (668.25 g/mol) (%): C: 61.02, H: 6.17, N: 12.56. Found (%): C: 61.2, H: 6.1, N: 12.6. Mass spectrometry MS (ESI^+^): *m*/*z* (calcd): 660.25, found: 659.73 [M + H^+^]. UV–vis: [DMSO, λ_max_/nm (ε/M^–1^ cm^–1^)]: 270 (38607); 293 (sh); 418 (2410). [CH_2_Cl_2_, λ_max_/nm (ε/M^–1^ cm^–1^)]: 270 (38701); 317 (sh); 423 (3528). FTIR [KBr, cm^–1^]: 3434 (br, H_2_O molecules); 2922 (ν_C–H_ aliphatic); 1646 (ν_C=N_); 1558 and 1448 (ν_C=C_ aromatic); 1113 (ν_C–O_).

#### 2.3.4 Single-Crystal X-ray Diffraction

The experimental data for all single crystals were recorded using the APEX3 software.^16^ The frames were integrated with the SAINT software using a narrow-frame algorithm.^17^ Data were corrected for absorption effects using the multiscan method (SADABS). The structures were solved and refined using the SHELXTL Software.^18^

CCDC 2251161 (for **Zn(L1)**_**2**_), 2251164 (for **Zn(L2)**_**2**_), 2251166 (for **Zn(L3)**_**2**_), 2251163 (for **Cu**_**2**_**(L1)**_**4**_), 2251162 (for **Cu**_**2**_**(L2)**_**4**_), and 2251165 (for **Cu(L3)**_**2**_) contain the supplementary crystallographic data for this paper. These data can be obtained free of charge from The Cambridge Crystallographic Data Centre via www.ccdc.cam.ac.uk/products/csd/request/.

**Zn(L1)**_**2**_: a red block-like crystal of C_34_H_40_N_6_O_2_Zn, approximate dimensions 0.040 mm × 0.050 mm × 0.100 mm, was used. The integration of the data using an orthorhombic unit cell yielded a total of 34,317 reflections to a maximum θ angle of 22.04° (0.95 Å resolution), of which 7273 were independent (average redundancy 4.718, completeness = 94.5%, *R*_int_ = 13.72%, *R*_sig_ = 11.27%) and 5372 (73.86%) were greater than 2σ(*F*^2^). The final cell constants of *a* = 8.3415(10) Å, *b* = 25.879(3) Å, *c* = 28.960(3) Å, and volume = 6251.6(13) Å^3^ are based upon the refinement of the *XYZ*-centroids of 3208 reflections above 20 σ(I) with 6.457° < 2θ < 43.14°. The structure was solved and refined using the space group *P*2_1_2_1_2_1_. The final anisotropic full-matrix least-squares refinement on *F*^2^ with 746 variables converged at *R*_1_ = 8.53% for the observed data and w*R*_2_ = 25.30% for all data. The goodness-of-fit was 1.120. The largest peak in the final difference electron density synthesis was 1.077 e^–^/Å^3^, and the largest hole was −0.963 e^–^/Å^3^ with an RMS deviation of 0.188 e^–^/Å^3^. On the basis of the final model, the calculated density was 1.339 g/cm^3^ and F_000_ 2656 e^–^.

**Zn(L2)**_**2**_: a dark red block-like crystal of C_32_H_36_N_6_O_4_Zn, approximate dimensions 0.080 mm × 0.100 mm × 0.150 mm, was used. The integration of the data using a monoclinic unit cell yielded a total of 127,929 reflections to a maximum θ angle of 27.54° (0.77 Å resolution), of which 14,334 were independent (average redundancy 8.925, completeness = 96.7%, *R*_int_ = 8.10%, *R*_sig_ = 4.51%) and 11,910 (83.09%) were greater than 2σ(*F*^2^). The final cell constants of *a* = 26.39(2) Å, *b* = 8.319(7) Å, *c* = 29.52(2) Å, β = 108.04(3)°, and volume = 6162.(9) Å^3^ are based upon the refinement of the *XYZ*-centroids of 9924 reflections above 20 σ(I) with 5.806° < 2θ < 54.26°. The structure was solved and refined using the space group *P*2_1_/*n*. The final anisotropic full-matrix least-squares refinement on *F*^2^ with 776 variables converged at *R*_1_ = 8.32% for the observed data and w*R*_2_ = 23.96% for all data. The goodness-of-fit was 1.048. The largest peak in the final difference electron density synthesis was 1.350 e^–^/Å^3^, and the largest hole was −0.845 e^–^/Å^3^ with an RMS deviation of 0.128 e^–^/Å^3^. On the basis of the final model, the calculated density was 1.367 g/cm^3^ and *F*_000_ 2656 e^–^.

**Zn(L3)**_**2**_: a red block-like crystal of C_34_H_40_N_6_O_4_Zn, approximate dimensions 0.120 mm × 0.120 mm × 0.330 mm, was used. The integration of the data using a monoclinic unit cell yielded a total of 136,193 reflections to a maximum θ angle of 28.38° (0.75 Å resolution), of which 15680 were independent (average redundancy 8.686, completeness = 99.3%, *R*_int_ = 2.96%, *R*_sig_ = 1.83%) and 13,823 (88.16%) were greater than 2σ(*F*^2^). The final cell constants of *a* = 26.525(17) Å, *b* = 8.140(5) Å, *c* = 30.553(17) Å, β = 107.00(2)°, and volume = 6309.(7) Å^3^ are based upon the refinement of the *XYZ*-centroids of 9199 reflections above 20 σ(*I*) with 5.545° < 2θ < 56.48°. The structure was solved and refined using the space group *P*2_1_/*n*. The final anisotropic full-matrix least-squares refinement on *F*^2^ with 978 variables converged at *R*_1_ = 7.43% for the observed data and w*R*_2_ = 18.85% for all data. The goodness-of-fit was 0.943. The largest peak in the final difference electron density synthesis was 1.550 e^–^/Å^3^, and the largest hole was −1.224 e^–^/Å^3^ with an RMS deviation of 0.093 e^–^/Å^3^. On the basis of the final model, the calculated density was 1.394 g/cm^3^ and *F*_000_ 2784 e^–^.

**Cu**_**2**_**(L1)**_**4**_: a green plate-like crystal of C_68_H_80_Cu_2_N_12_O_4_, approximate dimensions 0.040 mm × 0.310 mm × 0.330 mm, was used. The integration of the data using a monoclinic unit cell yielded a total of 96,755 reflections to a maximum θ angle of 27.74° (0.76 Å resolution), of which 13,482 were independent (average redundancy 7.177, completeness = 95.8%, *R*_int_ = 9.22%, *R*_sig_ = 6.78%) and 9950 (73.80%) were greater than 2σ(*F*^2^). The final cell constants of *a* = 11.8429(10) Å, *b* = 34.553(3) Å, *c* = 15.4582(13) Å, β = 108.999(3)°, and volume = 5981.0(9) Å^3^ are based upon the refinement of the *XYZ*-centroids of 9429 reflections above 20 σ(*I*) with 5.574° < 2θ < 55.18°. The structure was solved and refined using the space group *P*2_1_/*c*. The final anisotropic full-matrix least-squares refinement on *F*^2^ with 775 variables converged at *R*_1_ = 10.84% for the observed data and w*R*_2_ = 23.66% for all data. The goodness-of-fit was 1.229. The largest peak in the final difference electron density synthesis was 0.928 e^–^/Å^3^, and the largest hole was −2.180 e^–^/Å^3^ with an RMS deviation of 0.125 e^–^/Å^3^. On the basis of the final model, the calculated density was 1.395 g/cm^3^ and *F*_000_ 2648 e^–^.

**Cu**_**2**_**(L2)**_**4**_: a yellow-brown plate-like crystal of C_64_H_72_Cu_2_N_12_O_8_, approximate dimensions 0.020 mm × 0.120 mm × 0.300 mm, was used. The integration of the data using a triclinic unit cell yielded a total of 60814 reflections to a maximum θ angle of 27.59° (0.77 Å resolution), of which 13,298 were independent (average redundancy 4.573, completeness = 99.4%, *R*_int_ = 9.78%, *R*_sig_ = 9.64%) and 7716 (58.02%) were greater than 2σ(*F*^2^). The final cell constants of *a* = 11.8098(11) Å, *b* = 15.6385(15) Å, *c* = 17.6304(17) Å, α = 98.169(3)°, β = 104.908(3)°, γ = 108.875(3)°, and volume = 2885.5(5) Å^3^ are based upon the refinement of the *XYZ*-centroids of 9927 reflections above 20 σ(*I*) with 5.608° < 2θ < 54.00°. The structure was solved and refined using the space group *P*1̅. The final anisotropic full-matrix least-squares refinement on *F*^2^ with 775 variables converged at *R*_1_ = 5.83% for the observed data and w*R*_2_ = 16.85% for all data. The goodness-of-fit was 1.026. The largest peak in the final difference electron density synthesis was 1.612 e^–^/Å^3^, and the largest hole was −0.674 e^–^/Å^3^ with an RMS deviation of 0.099 e^–^/Å^3^. On the basis of the final model, the calculated density was 1.455 g/cm^3^ and *F*_000_ 1324 e^–^.

**Cu(L3)**_**2**_: a brown plate-like crystal of C_34_H_40_CuN_6_O_4_, approximate dimensions 0.080 mm × 0.160 mm × 0.400 mm, was used. The integration of the data using a monoclinic unit cell yielded a total of 66,608 reflections to a maximum θ angle of 30.51° (0.70 Å resolution), of which 4621 were independent (average redundancy 14.414, completeness = 99.7%, *R*_int_ = 2.48%, *R*_sig_ = 1.05%) and 4451 (96.32%) were greater than 2σ(*F*^2^). The final cell constants of *a* = 4.6248(3) Å, *b* = 27.1085(16) Å, *c* = 12.1597(7) Å, β = 97.052(2)°, and volume = 1512.95(16) Å^3^ are based upon the refinement of the *XYZ*-centroids of 9882 reflections above 20 σ(*I*) with 6.753° < 2θ < 60.97°. The structure was solved and refined using the space group *P*2_1_/*n*. The final anisotropic full-matrix least-squares refinement on *F*^2^ with 205 variables converged at *R*_1_ = 3.37% for the observed data and w*R*_2_ = 7.75% for all data. The goodness-of-fit was 1.202. The largest peak in the final difference electron density synthesis was 0.504 e^–^/Å^3^, and the largest hole was −0.345 e^–^/Å^3^ with an RMS deviation of 0.059 e^–^/Å^3^. On the basis of the final model, the calculated density was 1.449 g/cm^3^ and *F*_000_ 694 e^–^.

#### 2.3.5 Stability Studies

Stock solutions of each complex were freshly prepared in DMSO and diluted with HEPES buffer (10 mM, pH 7.4), ensuring that the organic solvent content was less than 5% (v/v). The samples were monitored by UV–vis absorption spectrophotometry for 6 consecutive hours, and a final measurement was done after 24 h.

#### 2.3.6 UV–Vis Spectrophotometric Titrations

The UV–vis titrations were performed with a 0.10 M carbonate-free KOH solution using a Metrohm 665 Dosimat buret and an Orion 710A pH-meter equipped with a Metrohm combined electrode (type 6.0234.100). The electrode system was calibrated to the pH = −log[H^+^] scale by means of blank titrations (HCl vs KOH) according to the method suggested by Irving et al.^19^ The average water ionization constant (p*K*_w_) is 13.76 ± 0.05. The path length was 1 cm, and the temperature was 25.0 ± 0.1 °C. An ionic strength of 0.10 M KCl was used to keep the activity coefficients constant. The samples were deoxygenated by bubbling argon through the system for 10 min. Spectrophotometric titrations were carried out using an Agilent Cary 8454 diode array spectrophotometer (Santa Clara, CA) in water on samples containing the ligands at 60 μM, in the pH range from 1.5 to 11.5 in the absence or in the presence of 1, 0.5, or 0.25 equiv of Cu(II) and Zn(II) ions, using 10.00 mL of sample volumes. Proton dissociation constants (*K*_a_) of the ligand precursors, as well as stoichiometry and overall stability constants (β) of the metal complexes, were calculated by the computer program PSEQUAD.^20^

#### 2.3.7 NMR Spectroscopic Titrations

A Bruker Avance III HD Ascend 500 Plus instrument (Billerica, MA) was used for NMR spectroscopic titrations and time-dependent studies applying a WATERGATE water suppression pulse scheme in the presence of 10% (v/v) D_2_O/H_2_O, and the sodium trimethylsilylpropanesulfonate (DSS) standard was added to samples as the internal reference. ^1^H NMR titrations were carried out for the ligand precursors in the presence of 0.1 M KCl. For free ligands and complexes’ stability measurements, ^1^H NMR spectra were recorded in DMSO-*d*_6_ or 30% (v/v) DMSO-*d*_6_/H_2_O.

#### 2.3.8 Lipophilicity and Solubility Studies

The distribution coefficients (*D*_7.4_) of the compounds were determined by the shake-flask method in *n*-octanol/buffered aqueous solution at pH 7.40 (25.0 ± 0.2 °C). The ligand precursors and the complexes were dissolved in 10 mM HEPES presaturated with *n*-octanol. The aqueous solution and *n*-octanol (1:1 ratio) were gently mixed with a 360° vertical rotation (∼20 rpm) for 3 h; then, the samples were centrifuged at 3000 rpm for 5 min. Two phases were separated, and their UV–vis spectra were recorded. The *D*_7.4_ value was calculated as follows
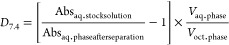
1

Thermodynamic solubility (*S*) of the free ligands and complexes was determined for the saturated solutions in water at pH 7.40 in 10 mM HEPES at 37.0 ± 0.2 °C. The concentration of the compounds was determined by UV–vis spectrophotometry using stock solutions with known concentrations dissolved in 100% DMSO, 50%, and 25% (v/v) DMSO/buffered aqueous solutions for the calibration.

#### 2.3.9 EPR

All CW-EPR spectra were recorded with a BRUKER EleXsys E500 spectrometer. The microwave frequency was 9.45 GHz. A microwave power of 13 mW, a modulation amplitude of 5 G, and a modulation frequency of 100 kHz were used. Two titrations were performed for Cu(II)–**L2** and Cu(II)–**L3** systems. For Cu(II)–**L2**, one was with 0.23 mM of Cu(II) and 0.25 mM of ligand concentration and another was with 0.23 mM of Cu(II) and 0.51 mM of ligand concentration, both in 30% (v/v) DMSO/water solution. In the case of the Cu(II)–**L3** system, the titrations were performed using 0.25 mM of Cu(II) and ligand and 0.23 mM of Cu(II) and 0.50 mM of ligand concentrations in 30% (v/v) DMSO/water solution. The titrations were done with a KOH solution. At different pH values, 0.2 mL of the sample was transferred into EPR quartz tubes and samples were frozen immediately. The solution EPR spectra were measured in a Dewar containing liquid nitrogen (77 K). For the Cu(II)–**L2** and Cu(II)–**L3** systems, 30 and 26 spectra were obtained, respectively. The powders of solid complexes **Cu**_**2**_**(L1)**_**4**_, **Cu**_**2**_**(L2)**_**4**_, and **Cu(L3)**_**2**_ were dissolved in pure DMSO, and their room temperature and frozen solution EPR spectra were also measured. For the latter spectrum, 0.2 mL of the DMSO stock solution was transferred into EPR tubes to which 25 μL of MeOH was added, and the EPR spectrum was recorded at a low temperature (77 K).

EPR spectra were simulated by the EPR simulation software of Rockenbauer and Korecz.^21^ Axial or rhombic *g*- and copper hyperfine *A*-tensors (*I*_Cu_= 3/2) were considered. Nitrogen splitting (*I*_N_ = 1), when resolved, was taken into account with rhombic superhyperfine couplings (*a*_x_^N^, *a*_y_^N^, *a*_*z*_^N^), where *x, y*, and *z* refer to the *g*-tensor orientations. For the description of the line width, orientation-dependent α, β, and γ parameters were used, where α, β, and γ define the line widths through the equation σ_MI_ = α + βM_I_ + γM_I_^2^, where *M*_I_ denotes the magnetic quantum number of the Cu(II) ion. Room-temperature spectra were fitted with the help of the obtained anisotropic data considering rotational averaging. The rotation correlation time (τ_*z*_), the anisotropy parameter (τ_*x*_/τ_*z*_), and the line widths (ω) were fitted. Since the natural CuCl_2_ was used for the measurements, spectra were calculated as the sum of the spectra of ^63^Cu and ^65^Cu weighted by their natural abundances. The hyperfine and superhyperfine coupling constants and the relaxation parameters were obtained in field units (Gauss = 10^–4^ T).

#### 2.3.10 Interaction with BSA

For the UV–vis spectrophotometric measurements, bovine serum albumin (BSA) stock solutions of lyophilized protein were first prepared in HEPES buffer (10 mM, pH 7.4) and allowed to hydrate for 24 h in a refrigerator. To evaluate the interaction of the complexes with BSA, equimolar solutions of BSA and Zn(II) complexes (55 and 250–265 μM) or Cu(II) complexes (35–53 and 330–400 μM) in 5% (v/v) DMSO/HEPES were prepared. An initial measurement was made right after the addition of the complex to the BSA solution and another one 30 min after. During the next 6 h, the spectrum was recorded every hour, and a final measurement was made after 24 h. Quartz cuvettes with an optical path of 1.0 cm were used, and the spectra were measured between 260 and 900 nm.

For the circular dichroism (CD) measurements, the BSA concentration was ca. 20 μM (in HEPES buffer). The circular dichroism (CD) spectra are the average of 3 scans measured between 250 and 500 nm. Parameters were as follows: scanning speed of 100 nm/min, bandwidth of 1 nm, and response of 2 s. Increasing amounts of the complexes’ DMSO stock solutions were added directly to the cuvette (1.0 cm) and yielded CD spectra under the same conditions after 10 min of equilibration. The CD spectrum of BSA was subtracted from all other spectra.

The steady-state fluorescence emission spectra of a solution of 0.5–1.0 μM BSA (in HEPES buffer) were recorded between 310 and 500 nm with λ_exc_ = 295 nm after additions of the complexes’ solutions directly to the cuvette (optical path of 1.0 cm). The UV–vis spectrum of each sample was used to make corrections and minimize the inner filter and reabsorption interferences.^22^ Solutions with the same concentration of complex and no BSA were recorded and used as blank spectra.

Data were analyzed by fitting a second-order equation, which accounts for both static and dynamic quenching
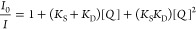
2where *I*_0_ and *I* are the fluorescence intensities in the absence and presence of quencher (*Q*), and *K*_S_ and *K*_D_ are the static and dynamic quenching constants, respectively.

The Stern–Volmer equation was used for low quencher concentrations to determine the Stern–Volmer quenching constant (*K*_SV_), a measure of the quenching strength
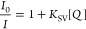
3

#### 2.3.11 Redox Properties

The reaction of selected zinc(II)–**Zn(L2)**_**2**_ and copper(II)–**Cu**_**2**_**(L2)**_**4**_ complexes with glutathione (GSH) and ascorbic acid (AA) was monitored spectrophotometrically, every 5 min, under anaerobic conditions at pH 7.4 in the presence of 5% (v/v) DMSO. The complex/GSH or AA ratio was 1:55 (*c*_complex_ = 55 μM, *c*_GSH_ or *c*_AA_ = 3 mM). Nitrogen was bubbled through the solutions before measurements, and the quartz Suprasil cells (10 mm optical path) were kept under nitrogen during spectrophotometric measurements. The reaction was followed until equilibrium was reached.

#### 2.3.12 Cell Culture Conditions and Antiproliferative Activity Studies

Cell culture media and antibiotics were obtained from Gibco (Thermo Fisher Scientific). Human malignant melanoma A375 cells (ATCC CRL-1619TM) and human keratinocytes HaCaT (CLS 300493) were cultured in DMEM-Glutamax supplemented with 10% fetal bovine serum (FBS), hereafter designated as complete medium. For human keratinocytes, 100 IU/mL of penicillin and 100 μg/mL of streptomycin (Gibco, Thermo Fisher Scientific) were also used. The cell lines were kept at 37 °C with a 5% CO_2_ atmosphere. The maintenance of cell cultures was performed every 2–3 days until the cells reached a confluency of about 80%.

The CellTiter-Glo 3D kit (Promega) was used to assess the viability of the A375 cells in the presence or absence (negative control) of increasing concentrations of the free ligands and complexes. The kit detects adenosine triphosphate, which is proportional to the number of viable cells, therefore allowing the assessment of cell proliferation. First, A375 cells were plated at a concentration of 5 × 10^4^ cells/mL in 96-well plates (200 μL/well) and cultured for 24 h in the culture conditions described above. Then, cells were incubated with the free ligands (1–100 μM), Zn(II) complexes (1 to 80 μM), and Cu(II) complexes (1–20 μM) in 96-well plates. After an incubation period of 48 h, 100 μL of each well was discarded. Then, 40 μL of CellTiter-Glo 3D reagent was added into each well and the contents were mixed vigorously for 15 min to induce cell lysis at room temperature. Finally, the luminescence was measured using a Varioskan Lux multimode plate reader (Thermo Scientific). Values presented are mean ± standard deviation (SD) of two independent biological experiments with 4 technical replicates per condition.

The MTT assay was used to assess cell viability in the presence of increasing concentrations of selected compounds. Briefly, HaCaT cells were plated at a concentration of 5 × 10^4^ cells/mL in 96-well plates (200 μL/well) and cultured for 24 h in the culture conditions described above. Then, cells were incubated with free ligands and metal complexes at concentrations ranging from 5 to 30 μM. Negative controls were cells maintained in complete culture medium. The culture medium was discarded after 48 h, cells were washed with PBS (2 times), and then 50 μL of MTT at 0.5 mg/mL in incomplete medium was added to all wells. After 2–3 h of incubation period at 37 °C, 100 μL of DMSO was added to each well to solubilize the formazan crystals, and absorbance was measured at 570 nm using a microplate reader Model 680 (Bio-Rad, CA). Two independent experiments were carried out, with six replicates per condition.

GraphPad Prism8 (GraphPad Software, San Diego, CA) was used to analyze cell proliferation, and values were plotted and fit a standard log dose–response curve to generate IC_50_ values.

#### 2.3.13 Guava ViaCount Assay

For the Guava ViaCount assay, A375 cells were plated at a concentration of 8 × 10^4^ cells/mL in 24-well plates (1 mL/well) and cultured for 24 h in the culture conditions described above.^23^ Cells were then incubated with **Zn(L2)**_**2**_ and **Cu**_**2**_**(L2)**_**4**_ at concentrations of 15 and 12 μM, respectively. The positive control, dacarbazine (DTIC), was tested at 70 μM. Cells in the presence of complete culture medium constituted the negative control group. After an incubation period of 48 h, cell culture supernatants were collected, and adherent cells were detached using TrypLEExpress (Invitrogen). Detached cells were then added to supernatants, and the wells were washed using PBS with 2% FBS to recover any remaining cells. The recovered suspension was centrifuged for 5 min at 700 *g*. The supernatants were discarded, and cells were suspended in PBS with 2% FBS. Then, on a 96-well plate, 20 μL of cell suspension was incubated with 180 μL of Guava ViaCount reagent (Merck Millipore, Darmstadt, Germany) for 5 min at r.t. Using a Guava EasyCyte 5HT Flow cytometer (Guava Technologies, Inc., Hayward, CA), data were collected and analyzed with the ViaCount software module with an acquisition of 10,000 events per sample. GraphPad Prism8 was used to analyze cell proliferation (GraphPad Software, San Diego, CA). One experiment was carried out, with three replicates per condition.

## 3 Results and Discussion

The work was initiated with the synthesis of the compounds **L1**–**L3**, which were obtained by a base-catalyzed condensation between equimolar amounts of 8-hydroxy-2-quinolinecarboxaldehyde (8HQ-2CHO) and three different piperidine/morpholine-type amines. The ligand precursors were reacted (*in situ*) with ZnCl_2_ or CuCl_2_ in ethanol or methanol, yielding Zn(II) [**Zn(L1)**_**2**_–**Zn(L3)**_**2**_] or Cu(II) [**Cu**_**2**_**(L1)**_**4**_, **Cu**_**2**_**(L2)**_**4**_, and **Cu(L3)**_**2**_] complexes, in moderate to good yields (Scheme 1), using methodologies similar to those described in the literature for related compounds.^9,10,12,24^ The complexes are stable under air and moisture in the solid state, in contrast to the free ligands, which are hygroscopic and therefore were stored in sealed ampules.

All ligand precursors and complexes were characterized by elemental analysis (CHN) and by the usual spectroscopic techniques in both the solid state and solution. The final compounds’ elemental analyses (Experimental Section) and ESI-MS data (Supporting Information (SI), Table S1) are in good agreement with the expected structural formulas. Single crystals were obtained for all complexes, allowing the determination of their molecular structures by single-crystal X-ray diffraction (SC-XRD) analysis.

### 3.1 NMR Spectroscopic Characterization of the Ligands and Zn(II) Complexes

The ^1^H NMR spectra of the free ligands and corresponding Zn(II) complexes (Figures S1 and S2) were recorded in DMSO-*d*_6_ and are in accordance with the proposed structures. The successful synthesis of compounds **L1**–**L3** was confirmed by the presence of the H_11_ proton in the azomethine group (HC = N) that resonates as a singlet around 8.48 ppm, in good agreement with analogous compounds.^9,10^ The aromatic protons of the 8HQ ring are found between 8.05 and 6.86 ppm, of which the most deshielded is the H_4_ proton. Upon complexation with Zn(II), downfield shifts are observed for both H_11_ and H_4_ due to coordination with the nitrogen lone pair of electrons of the azomethine (CH = N) and to the effect of the positively charged nitrogen of the 8HQ ring, respectively. The main ^13^C NMR chemical shifts exhibited by the complexes show downfield and upfield shifts of the hydroxyl carbon (C_8_) and azomethine (HC = N) carbon (C_11_), respectively, compared with the free ligand, which further confirms coordination of the azomethine nitrogen and hydroxyl oxygen atoms to the metal center.

### 3.2 EPR Spectroscopic Measurements of the Isolated Cu(II) Complexes

**Figure 1:**
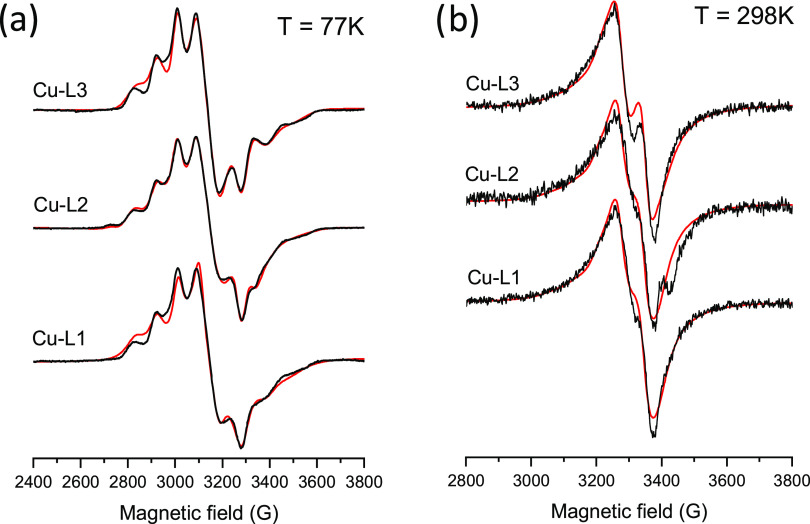
Measured (black) and simulated (red) EPR spectra of complexes **Cu**_**2**_**(L1)**_**4**_, **Cu**_**2**_**(L2)**_**4**_, and **Cu(L3)**_**2**_ dissolved in DMSO and recorded at (a) low temperature (77 K) and (b) room temperature (298 K). The simulation data are listed in Table 1 [*c*_complex_ = 0.8 mM].

The Cu(II) complexes, **Cu**_**2**_**(L1)**_**4**_, **Cu**_**2**_**(L2)**_**4**_, and **Cu(L3)**_**2**_, were dissolved in DMSO, and their EPR spectra were recorded at both low and room temperatures (Figure 1), and the determined EPR parameters are included in Table 1. The frozen solution EPR spectra of the dissolved complexes revealed a strongly rhombic *g*-tensor, which reflects that the ground state of the unpaired electron is a linear combination of the d_*x*2-*y*2_ and d_*z*2_ orbitals. In regular situations, when the geometry is elongated octahedral, square-pyramidal, or square planar, the ground state is the d_*x*2-*y*2_ orbital and the relation *g*_*z*_ > *g*_*y*_ ≈ *g*_*x*_ > 2.0023 is expected (normal spectrum). In the compressed octahedral or trigonal bipyramidal geometry, the ground state is the d_*z*2_ orbital and an “inverse” spectrum can be measured (*g*_*x*_ ≈ *g*_*y*_ > *g*_*z*_ > 2.0023). The distortion parameter *R* = (*g*_2_ – *g*_3_)/(*g*_1_ – *g*_2_) (where *g*_1_ > *g*_2_ > *g*_3_) was used^25^ to calculate the predominance of the d_z2_ or d_x2-y2_ orbital. If *R* > 1, the greater contribution to the ground state arises from the d_*z*2_ orbital and if *R* < 1, it arises from the d_*x*2-*y*2_ orbital. In the dissolved complexes, the obtained values fall between 0.69 < R < 1 (Table 1), showing an intermediate case when a mixture of the two-ground states is detected with a somewhat greater contribution from the d_*x*2-*y*2_ orbital. This finding suggests a compressed octahedral or strongly distorted octahedral geometry of the complexes, appearing as monomers when dissolved in DMSO. These results suggest the NNO coordination of both ligands around the Cu(II) ion in a distorted octahedral geometry similar to that of the Zn(II) complexes detected by the SC-XRD technique (described in Section 3.5.1). The recorded room-temperature EPR spectra also support this geometry since instead of the four-line spectrum of the usual elongated octahedral, a broad singlet-like spectrum was obtained. The spectra could be satisfactorily described by the rotational averaging of the anisotropic parameters determined in the frozen solution, with parameters collected in Table 1.

**Table 1:** EPR Parameters Obtained by the Simulation of Frozen Solution and Room-Temperature Spectra of the **Cu**_**2**_**(L1)**_**4**_, **Cu**_**2**_**(L2)**_**4**_, and **Cu(L3)**_**2**_ Complexes Dissolved in DMSO

	*g*_*x*_	*g*_*y*_	*g*_*z*_	*|A*_*x*_|/10^–4^ cm^–1^	*|A*_*y*_*|*/10^–4^ cm^–1^	*|A*_*z*_*|*/10^–4^ cm^–1^	*R*	τ_*z*_/s	τ_*x*_/τ_*z*_	ω/G
*T* = 298 K										
**Cu**_**2**_**(L1)**_**4**_	2.017	2.110	2.240	84	24	105	0.72	4.5 × 10^**–**9^	0.09	25
**Cu**_**2**_**(L2)**_**4**_	2.017	2.110	2.240	84	24	105	0.72	4.5 × 10^**–**9^	0.09	25
**Cu(L3)**_**2**_	2.020	2.110	2.240	89	25	115	0.69	3.0 × 10^**–**9^	0.09	25
*T* = 77 K										
**Cu**_**2**_**(L1)**_**4**_	2.018	2.123	2.267	99	43	90	0.73	-	-	-
**Cu**_**2**_**(L2)**_**4**_^a^	2.027	2.126	2.269	91	26	88	0.69	-	-	-
**Cu(L3)**_**2**_	2.020	2.143	2.265	101	26	85	1.01	-	-	-

a For the fitting of these spectra, 12% of isomer 2 and 6% of component 2 (given in SI,Table S3) were also taken into account.

### 3.3 UV–Vis Characterization of the Ligands and Complexes

**Figure 2:**
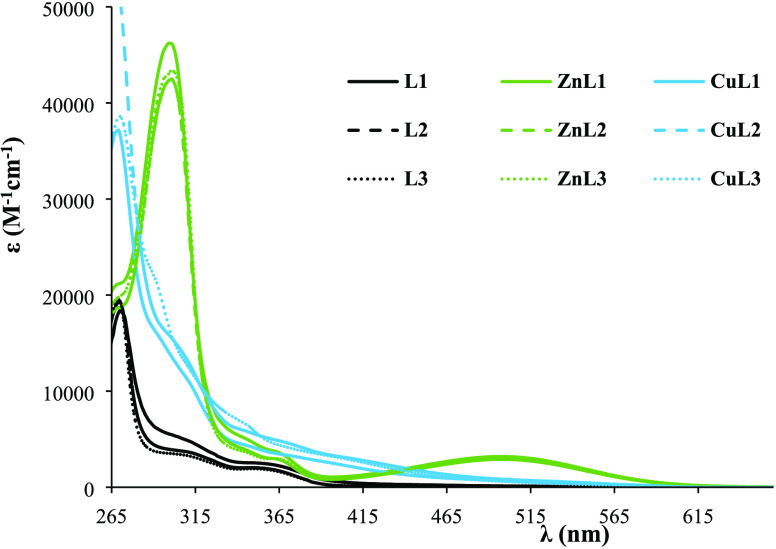
UV–vis molar absorption spectra of the ligands (black) and respective Zn(II) (green) and Cu(II) (blue) complexes in DMSO [*c* = 15–100 μM].

The molecular absorption spectra of all complexes were recorded using 1.0 × 10^–5^–1.0 × 10^–4^ M solutions in CH_2_Cl_2_ and DMSO. Figure 2 shows the spectra of the free ligands and metal complexes in DMSO, whereas Table S2 presents the values obtained for the molar absorptivity coefficient (ε) and the corresponding wavelength (λ_max_).

The electronic absorption spectra of the Schiff bases **L1–L3** in DMSO show one intense absorption band at ∼270 nm and two shoulders in the region 260–400 nm that are assigned to intraligand *n* → π* transitions of the azomethine group. The π → π* transitions of the aromatic rings of 8HQ are below the cutoff of the solvent but can be observed in methanol (see Figure S3 for **L3**). After complexation, the bands of the Cu(II) complexes appear nearly in the same wavelength as in the spectra of their corresponding ligands, revealing that these are intraligand transitions and not much affected by coordination to this metal ion. On the other hand, for the Zn(II) complexes, a bathochromic shift is clearly evident, indicating coordination of the ligand to metal through the azomethine moiety.^26^

Additionally, both Zn(II) and Cu(II) complexes show the presence of a broad band in the visible region that can be ascribed to charge-transfer bands.^27^ In fact, to corroborate this information, the UV–vis spectrum of all complexes was also measured in CH_2_Cl_2_, showing a wavelength shift of 5–10 nm. In methanol, the shift is ca. 30 nm (see Figure S4 for **Zn(L3)**_**2**_). The shoulder band at ∼420 nm observed in the Cu(II) complexes’ spectra (only visible at higher concentrations; Figure S5) can be assigned to a ligand-to-metal charge-transfer (LMCT) band. Moreover, due to the low solubility of the complexes at higher concentrations and the weak intensity of d–d bands (Laporte forbidden), these are not revealed in the spectra recorded for the Cu(II) complexes.

Fluorescence emission spectra were measured for the Zn(II) complexes and are included in Figure S6. Excitation in the UV (at 350 nm) results in emission bands of moderate intensity at *ca*. 450–500 nm. The Cu(II) complexes show only very weak bands (data not shown).

### 3.4 FTIR Characterization

The solid-state FTIR spectra obtained for the free ligands and corresponding complexes display bands that are comparable with those of similar complexes bearing 8-hydroxyquinoline-imine ligands, reported in the literature.^9,28^ They reveal the presence of a band corresponding to the stretching vibration of the azomethine moiety, ν(C = N), at ∼1649 cm^–1^, as expected for a Schiff base coupled to an aromatic ring.^29^ This band shifts to lower wavenumbers (1639–1643 cm^–1^) in the Zn(II) complexes, indicating a decrease in the C = N bond order due to the bond formed between the metal and the imine nitrogen lone pair.^30^ This observation is in line with the results obtained by NMR spectroscopy. All Cu-complex spectra are very similar, suggesting the same type of coordination among the complexes and a decrease in the intensity of the C = N bond vibration when compared to the ligands.

The bands observed at the regions 3050–3053 and 2934–2960 cm^–1^ correspond to the C–H stretching vibrations of the piperidine/morpholine and pyridine, respectively. The peaks observed at 1434–1455 and 1558–1594 cm^–1^ correspond to the stretching C = C vibrations of the quinoline ring. The broad bands centered at *ca*. 3434 cm^–1^ are due to solvent molecules such as water and to OH stretching vibrations.

### 3.5 Single-Crystal X-ray Diffraction

**Figure 3:**
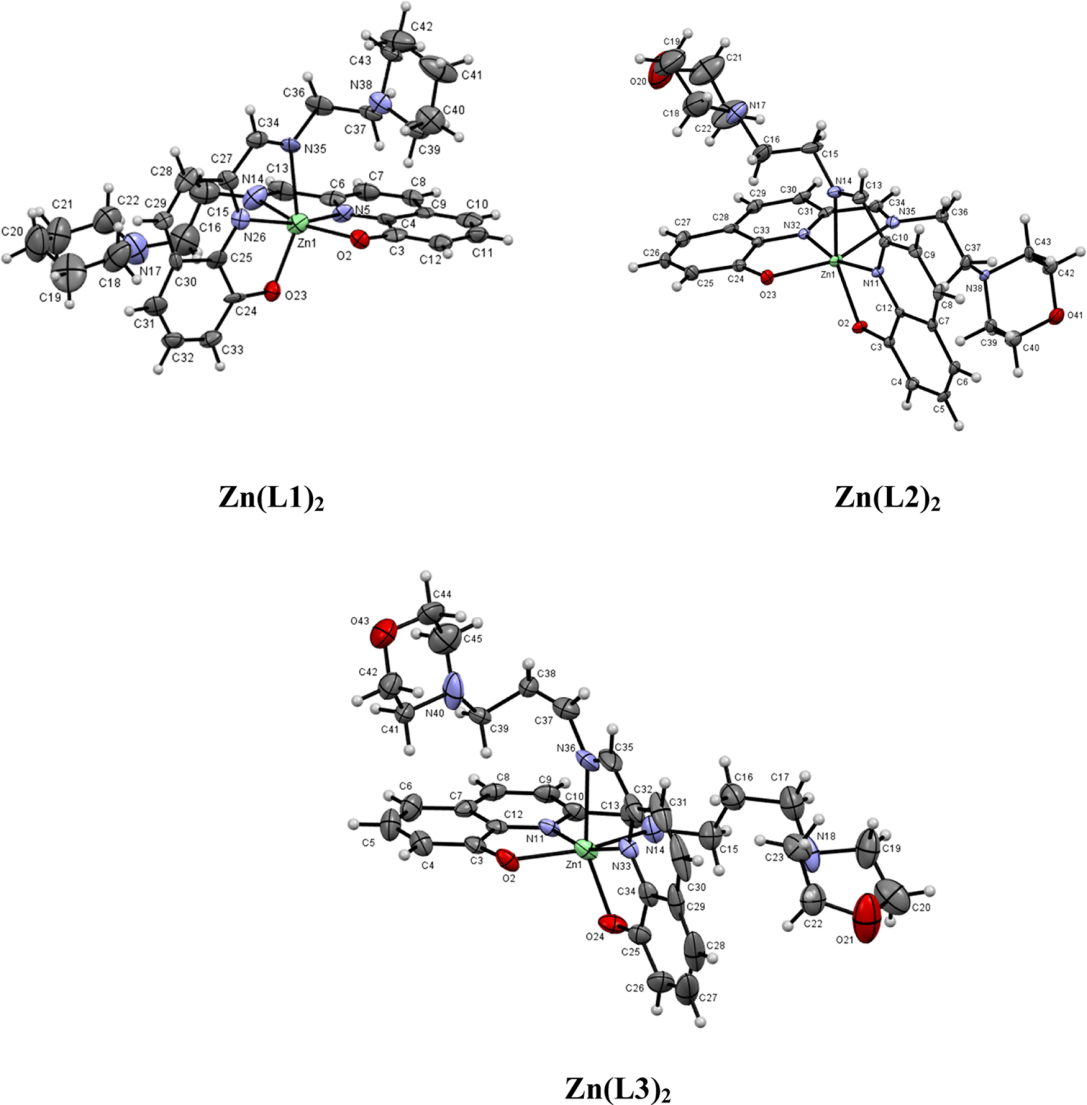
Molecular structure of the Zn(II) complexes determined by SC-XRD.

**Figure 4:**
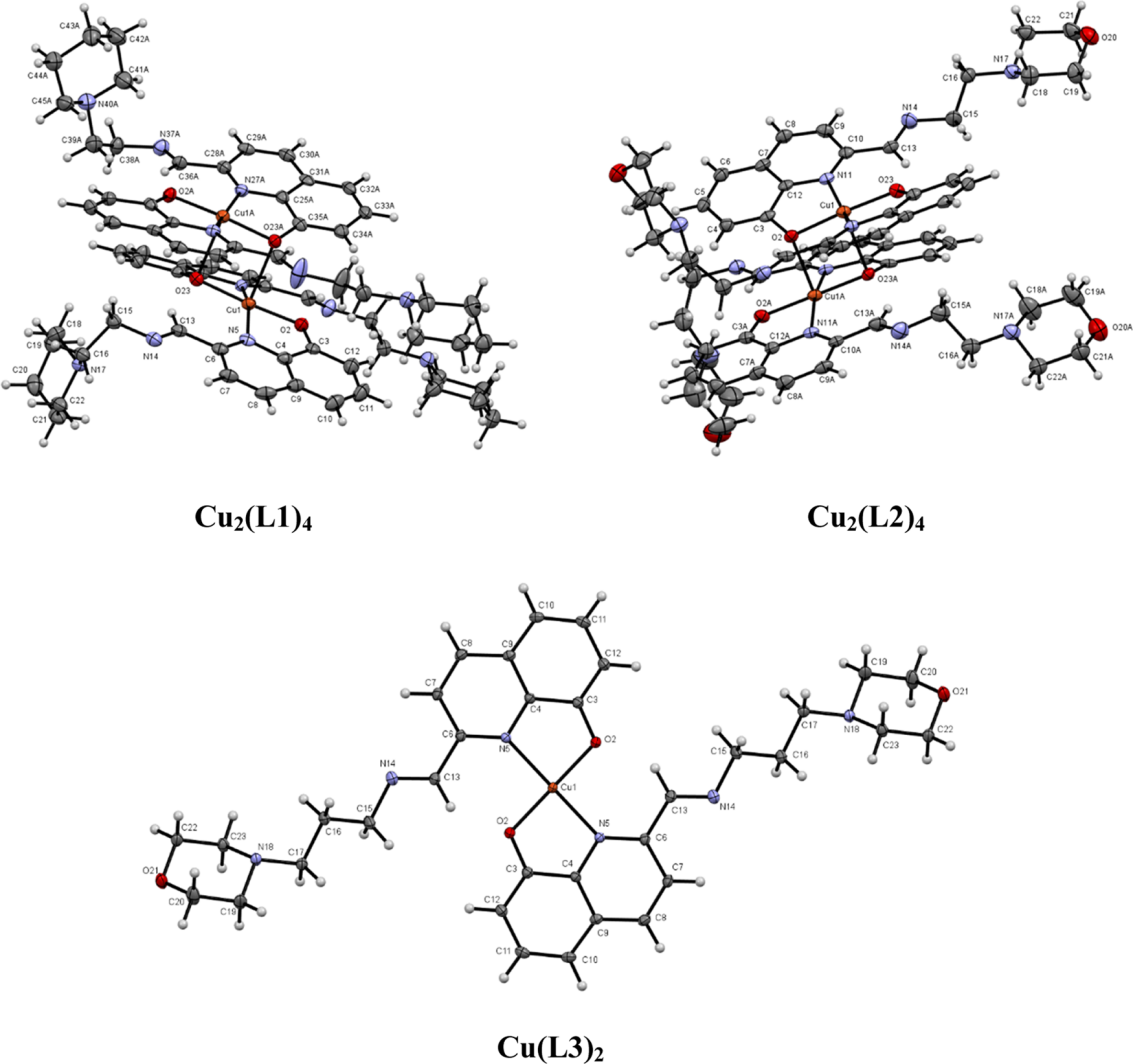
Molecular structure of the Cu(II) complexes determined by SC-XRD analyses.

The crystal structures of the Zn(II) and Cu(II) complexes were solved by X-ray diffraction analysis. Figures 3 and 4 display the ORTEP diagrams of their molecular structures. The main crystallographic data and selected bond distances and angles can be found in the Experimental part and the Supporting Information sections (Table S3).

#### 3.5.1 Molecular Structures of the Zn(II) Complexes

The **Zn(L1)**_**2**_ compound crystallizes in the orthorhombic system, space group *P*2_1_2_1_2_1_, whereas **Zn(L2)**_**2**_ and **Zn(L3)**_**2**_ crystallize in the monoclinic system, space group *P*2_1_/*n*. For all Zn complexes, the unit cells display two enantiomers in the racemic crystal. All complexes contain a six-coordinated Zn(II) ion with a distorted octahedral geometry (Figure 3), to which two monodeprotonated ligands, arranged in a meridional fashion, are coordinated. Each metal, in the corresponding compound, is coordinated in an NNO arrangement of donor atoms from two ligands, with a *cis* orientation of the phenolate moieties [O(2)–Zn-O(23) = 98.0(5)°, O(2)–Zn-O(23) = 98.0(15)°, and O(2)–Zn-O(24) = 101.21(12)° for **Zn(L1)**_**2**_, **Zn(L2)**_**2**_, and **Zn(L3)**_**2**_, respectively]. For the coordinated N atoms, the imine nitrogens are also *cis* to one another, [N(14)–Zn-N(35) = 84.5(6)°, N(14)–Zn-N(35) = 85.71(17)°, and N(14)–Zn-N(36) = 90.62(16)° for **Zn(L1)**_**2**_, **Zn(L2)**_**2**_, and **Zn(L3)**_**2**_, respectively], whereas the pyridine ones are *trans* to one another [N(5)–Zn-N(26) = 161.9(6)°, N(11)–Zn-N(32) = 164.89(18)°, and N(11)–Zn-N(33) = 162.28(12)° for **Zn(L1)**_**2**_, **Zn(L2)**_**2**_, and **Zn(L3)**_**2**_, respectively]. The *cis* orientation of the phenolate moieties and *trans* orientation of the pyridines have been observed in other Zn(II) complexes with 8-hydroxyquinoline derivatives.^31^ The distortion of the octahedral coordination sphere is observed in the angles around the Zn(II) ions, which are different from the ideal angles of 90° for a perfect octahedral. This fact is a consequence of the geometrical restrictions imposed by the NNO binding of the tridentate ligands.

The Zn–O bond distances in the complexes are in the same range (2.045(3)–2.096(3) Å) and not equivalent for each compound, similar to other Zn(II) complexes described in the literature.^32^ On the other hand, the two Zn–N_imine_ bond distances are in general longer than the two Zn–N_quinoline_ (see Table S3). The Zn–N_quinoline_ bonds are *trans* to the other Zn–N_quinoline_ bonds. Thus, the lower distances are consistent with the presence of small Zn–N back bonding. In addition, two intramolecular hydrogen bonds between the neighboring ligands are displayed in the three structures (O23–H16B = 2.750 Å, O2–H37A = 2.796 Å for **Zn(L1)**_**2**_; O23–H16B = 2.836 Å; O2–H37A = 2.715 Å for **Zn(L2)**_**2**_; and O24–H15D = 3.280 Å; O2–H39D = 2.893 Å for **Zn(L3)**_**2**_).

#### 3.5.2 Molecular Structures of the Cu(II) Complexes

Complexes **Cu**_**2**_**(L1)**_**4**_ and **Cu**_**2**_**(L2)**_**4**_ crystallize in the monoclinic system, space group *P*2_1_/*c*, and triclinic system, space group *P*1̅, respectively. Both compounds display dinuclear structures where the two Cu(II) ions show a square-pyramidal geometry (Figure 4), chelated by two monodeprotonated ligands, with the basal positions formed by two N atoms and O atoms from two 8HQ moieties of the ligands (**L1** or **L2**), both in *trans* position to each other, while the fifth axial coordination position is occupied by other O atoms (O(23) and O(23A) in **Cu**_**2**_**(L1)**_**4**_; O(2) and O(23A) in **Cu**_**2**_**(L2)**_**4**_) from another ligand, which forms part of the basal position of the neighboring Cu atom of the dinuclear unit. These axial Cu(1)–O(23A) and Cu(1A)–O(23) distances of 2.381 and 2.360 Å in **Cu**_**2**_**(L1)**_**4**_ are longer than the basal distances Cu(1)–O(2) and Cu(1)–O(23) (1.886 and 1.914 Å) and Cu(1A)–O(2A) and Cu(1A)–O(23A) (1.896 and 1.904 Å). The same trend is observed for **Cu**_**2**_**(L2)**_**4**_ (Table S3). The copper–copper distances in the dinuclear complexes are of 3.146 and 3.171 Å for **Cu**_**2**_**(L1)**_**4**_ and **Cu**_**2**_**(L2)**_**4**_, respectively, slightly shorter than in other dinuclear quinolinate complexes.^14^ The structures are stabilized by four intramolecular hydrogen bonds [O2A-H36C = 2.181 Å; O23A-H13A = 2.112 Å; O23–H13 = 2.208 Å; O2–H34 = 2.169 Å in **Cu**_**2**_**(L1)**_**4**_; and O2A-H34A = 2.142 Å; O23A-H13A = 2.179 Å; O23–H13 = 2.160 Å; O2–H34 = 2.136 Å in **Cu**_**2**_**(L2)**_**4**_]. The angles O–Cu–N of the chelate ligands in the complexes reflect the geometrical restrictions imposed by these ligands, and the values are similar in both compounds (81.74–84.06°). The packing of the two complexes shows the neighboring dinuclear compounds placed face to face with their basal planes (Figure S7).

The molecular structure of complex **Cu(L3)**_**2**_ corresponds to a monomeric Cu(II) complex that crystallizes in the monoclinic system in the space group *P*2_1_/*n*. The metal adopts a four-coordinated square planar geometry with Cu(II) coordinated by two ligands that act as bidentate through the (N,O) atoms, the quinoline N, and the phenolate O. The N-donor and the O-donor atoms are located *trans* to each other. The angles N(5)–Cu(1)–O(2) of the chelate ring are 83.94° in the same range as those observed in **Cu**_**2**_**(L1)**_**4**_ and **Cu**_**2**_**(L2)**_**4**_. The Cu–O distances are shorter than the Cu–N distances (1.891 vs 2.087 Å). The values are in the range shown by other related compounds described in the literature.^33^

The packing structure of the compound shows additional intermolecular hydrogen bonds that stabilize the crystal lattice between the neighboring ligand moieties. Intermolecular hydrogen bonds are displayed between the O(2) and H(13) atoms of two neighboring molecules (O2–H13 = 2.139 Å; Figure S7).

### 3.6 Evaluation of the Stability by UV–Vis and ^1^H NMR Spectroscopies

The compounds’ stability in solution and particularly in aqueous media at physiological pH is an important property to evaluate prior to its biological assessment to ensure that the complexes do not precipitate in aqueous environment and that they are stable to hydrolysis and decomposition in the time span of the studies. The evaluation of the stability of the free ligands and complexes was done by UV–vis and ^1^H NMR spectroscopic measurements in different solvent conditions and for 8 days at room temperature.

Compounds **L1–L3** are Schiff bases; therefore, they may undergo hydrolysis in the presence of water. As a first step, the stability was monitored in DMSO-*d*_6_ and 30% (v/v) DMSO-*d*_6_/H_2_O and spectral changes were followed with time (see representative spectra for **L3** in Figure S8). It was found for all free ligands that up to 10 h the spectra remained almost unchanged in both media, but after that, novel peaks appeared in the spectra and the signals belonging to the azomethine group (*H*C = N, δ *ca*. 8.5 in DMSO) decreased their intensity.

UV–vis spectra were recorded for the Schiff bases **L1–L3** at pH 7.4 and concentrations in the range of 141–228 μM in a buffered medium (10 mM HEPES, with 5% (v/v) DMSO) (see Figures S9 and S10). Some spectral changes were observed; however, they were minor during the first 24 h. Based on these findings, fresh stock solutions of the free ligands were always used for the titrations.

The stability of the Zn(II) and Cu(II) complexes was evaluated in 100% DMSO and in the presence of aqueous buffered media (HEPES, 10 mM, pH 7.4). The latter media was used to mimic physiological conditions. All complexes, evaluated in the concentration range of ca. 35–400 μM, displayed adequate stability in the organic solvent (Figure S11), namely, the ^1^H NMR spectra recorded for the Zn(II) complex of **L1** (in 1 mM concentration) remained unchanged during the tested 8 days period. It should be noted that the coordination of the metal ions stabilizes these Schiff bases (Figure S12). However, in aqueous media containing 5% (v/v) DMSO (Figure S13), evaluated in the range of ca. 35–50 μM for Cu(II) complexes and 100–135 μM for Zn(II) complexes, only the Cu(II) complexes showed no significant changes in the UV–vis absorption spectra over time, indicating adequate stability (average of less than 3% variation over a 24 h period). The Zn(II) complexes, particularly **Zn(L1)**_**2**_ and **Zn(L2)**_**2**_, evaluated at ca. 100–120 μM, did not show signs of decomposition but demonstrated some solubility problems, indicated by a global decrease in absorbance values. However, since lower concentrations are used in the biological assays, we anticipate that both stability and solubility will not be compromised. **Zn(L3)**_**2**_ showed a different absorption profile developing with time, which suggests hydrolysis.

### 3.7 Proton Dissociation Processes and Complexation with Cu(II) and Zn(II) Ions

**Figure 5:**
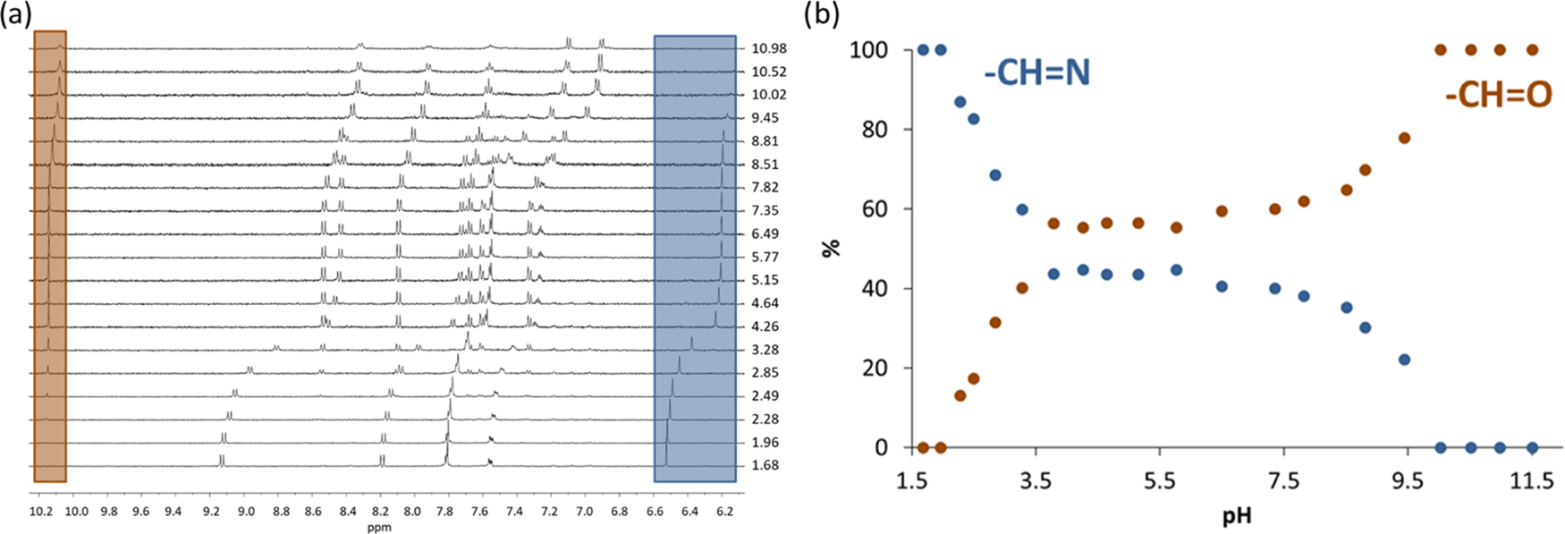
(a) ^1^H NMR spectra of **L2** at various pH values and (b) peak intensities of the HC = N or HC = O protons from ^1^H NMR spectra of **L2** plotted against the pH [*c*_L_ = 415 μM; *t* = 25 °C; *I* = 0.10 M (KCl), 10% (v/v) D_2_O/H_2_O].

As the UV–vis and ^1^H NMR spectra obtained for the ligand precursors revealed the possible partial decomposition in DMSO and in the DMSO/H_2_O solvent mixtures, ^1^H NMR spectra were recorded for **L2** and **L3** at various pH values (Figures 5, S14, and S15). The titration of **L2** was started at an acidic pH, and the total duration of the titration was ca. 4 h. By increasing the pH, the peak assigned to the azomethine proton (*H*C = N) decreased the intensity and a new peak assigned to the *H*C = O proton of the formed aldehyde appeared at pH > 2.5 and it became dominant at pH > 10 (Figure 5). This confirmed the hydrolysis of **L2**, and compound **L3** behaved similarly (Figure S15).

**Figure 6:**
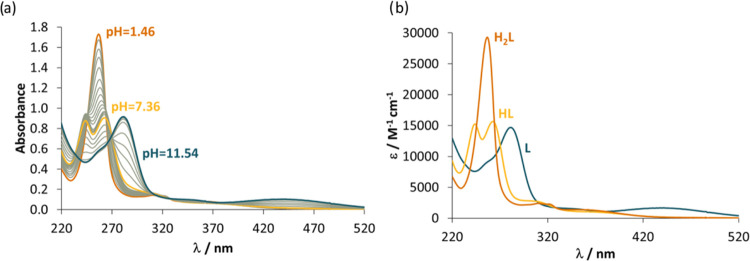
(a) UV–vis spectra of **L1** recorded at various pH values and (b) calculated individual molar absorption spectra for the species in different protonation states. Notes: the morpholinium nitrogen deprotonation was not accompanied by spectral changes. As two p*K*_a_ values could be computed, the fully protonated form is denoted here as H_2_L [*c*_L_ = 60 μM; *t* = 25 °C; *I* = 0.10 M (KCl)].

Next, the proton dissociation processes of the free ligands were followed by UV–vis titrations in aqueous solution. Their duration was much shorter (and the concentration used was lower: 60 vs 415 μM) compared to the ^1^H NMR titrations, but partial decomposition is still possible. These compounds possess three protons that may dissociate in the measured pH range (1.5–11.5), namely, at the quinolinium nitrogen (N_q_H^+^), the phenol OH, and the amine nitrogen (NH^+^) of the heterocycle. The latter one is not present in the chromophoric moiety; thus, its deprotonation is not expected to be accompanied by significant UV–vis spectral changes. Representative UV–vis spectra are shown in Figure 6a for **L1**, which reveal two well-separated processes, and the presence of isosbestic points indicating that in the pH range of each deprotonation process only two species are in equilibrium. This would not be the case if the Schiff base decomposed significantly.

Based on the measured UV–vis spectra, p*K*_a_ values (Table 2) were computed in addition to the molar absorbance spectra (see Figure 6b for **L1**) of the individual species in different protonation states (H_2_L, HL, L). The lower p*K*_a_ values of the free ligands were attributed to the quinolinium nitrogen and the higher one to the OH group. These values are similar for the three studied ligands and are lower than those of the reference compound 8-hydroxyquinoline [p*K*_a_ (N_q_H^+^) = 4.99, p*K*_a_ (OH) = 9.51],^14^ most probably due to the mesomeric effect of the azomethine moiety stabilizing the conjugate base forms. The pH-dependent ^1^H NMR spectra could not be used to calculate p*K*_a_ values due to the ligand decomposition; however, a third deprotonation process could be detected in the pH range of ca. 5–7 based on the changes of the methylene peaks in the upfield range (for **L2** and **L3**, see Figures S14 and S15, respectively). Most likely, this is due to the deprotonation of the morpholinium nitrogen. Globally, the obtained p*K*_a_ values indicate that the free ligands are found in their neutral forms with mostly deprotonated heterocyclic nitrogens at pH 7.4.

**Table 2:** Proton Dissociation Constant (p*K*_a_) Values of **L1**–**L3** and Overall Stability (Formation) Constants (log β) of the Complexes Determined by UV–Vis Spectrophotometric Titrations [*I* = 0.1 M KCl; *t* = 25.0 °C]

	L1	L2	L3
p*K*_a_ (N_q_H^+^)	2.80 ± 0.03	2.83 ± 0.03	2.77 ± 0.03
p*K*_a_ (OH)	8.77 ± 0.03	8.84 ± 0.03	8.78 ± 0.03
log β (CuL)	8.36 ± 0.01	7.98 ± 0.02	8.34 ± 0.01
log β (CuL_2_)	15.60 ± 0.02	15.77 ± 0.03	15.57 ± 0.01
log β (ZnL)	6.90 ± 0.03	6.86 ± 0.04	6.87 ± 0.01
log β (ZnL_2_)	14.42 ± 0.01	14.32 ± 0.02	13.68 ± 0.04

**Figure 7:**
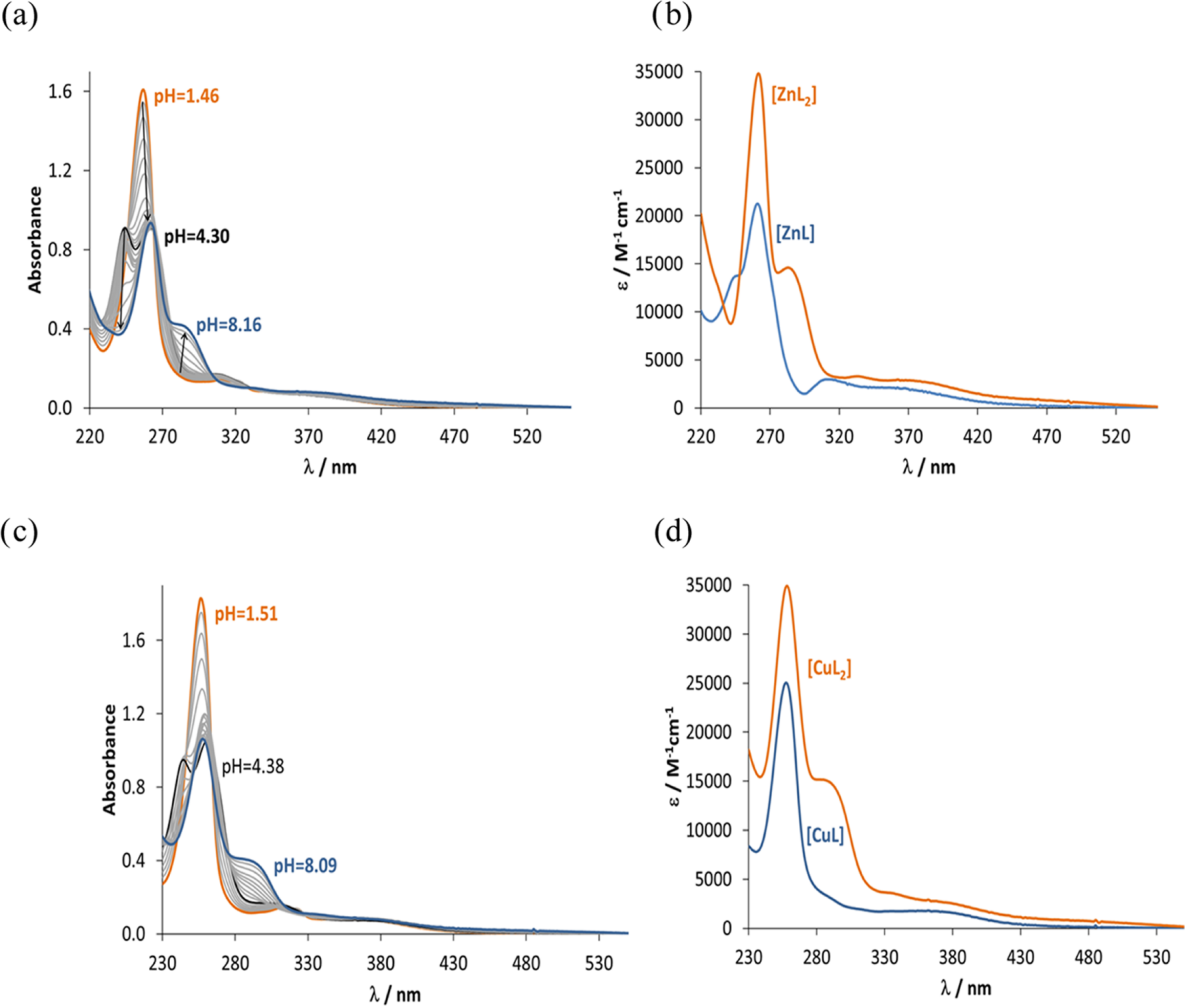
UV–visible spectra of (a) the Zn(II)–**L1** system recorded at various pH values and (b) calculated individual molar absorption spectra for the complex species [*c*_L_ = 57 μM; *c*_Zn(II)_ = 28 μM; *t* = 25 °C; *I* = 0.10 M (KCl)]. (c) UV–visible spectra of the Cu(II)–**L2** system recorded at various pH values and (d) calculated individual molar absorption spectra for the complex species [*c*_L_ = 58 μM; *c*_Cu(II)_ = 32 μM; *t* = 25 °C; *I* = 0.10 M (KCl)].

**Figure 8:**
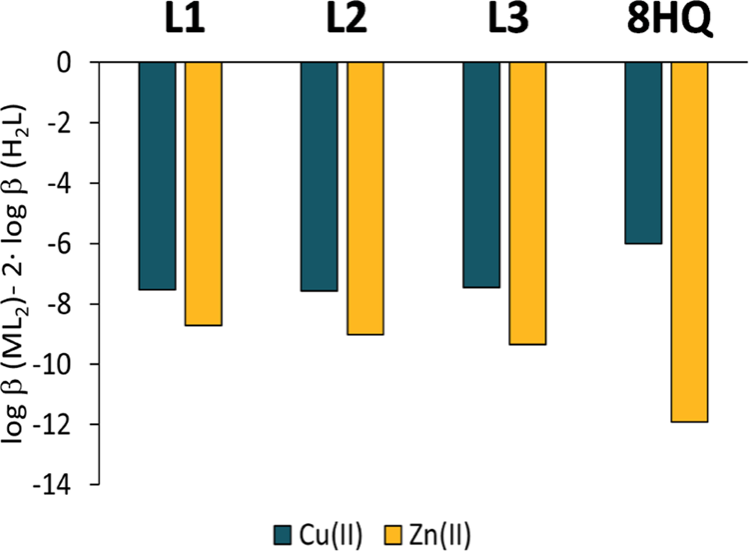
Derived stability constants of the bis-ligand complexes corrected by ligand basicity. Data for 8-hydroxyquinoline (8HQ) are also shown [*c*_L_ = 60 μM; *c*_Cu(II)_ = 30 μM; *t* = 25 °C; *I* = 0.10 M (KCl)].

UV–vis spectrophotometric titrations were done for the metal (Cu(II), Zn(II))–ligand (**L1**, **L2**, **L3**) systems (see representative spectra in Figure 7), and the spectral changes could be interpreted assuming the formation of mono- and bis-ligand complexes (Table 2). To compare the stability of the complexes, derived stability constants were calculated for the bis-ligand complexes taking into consideration the different proton dissociation constants of the ligands (Figure 8). These values show that the Cu(II) complexes possess higher stability than the Zn(II) complexes. Comparing these constants to those of 8-hydroxyquinoline,^14,34^ it can be concluded that the Cu(II) complexes of these Schiff bases have lower stability, while the Zn(II) species have higher stability than the corresponding 8HQ complexes. In the latter case, the higher stability most probably is the consequence of the tridentate coordination of the 8HQ-Schiff base ligands, as shown by the crystallographic analysis.

**Figure 9:**
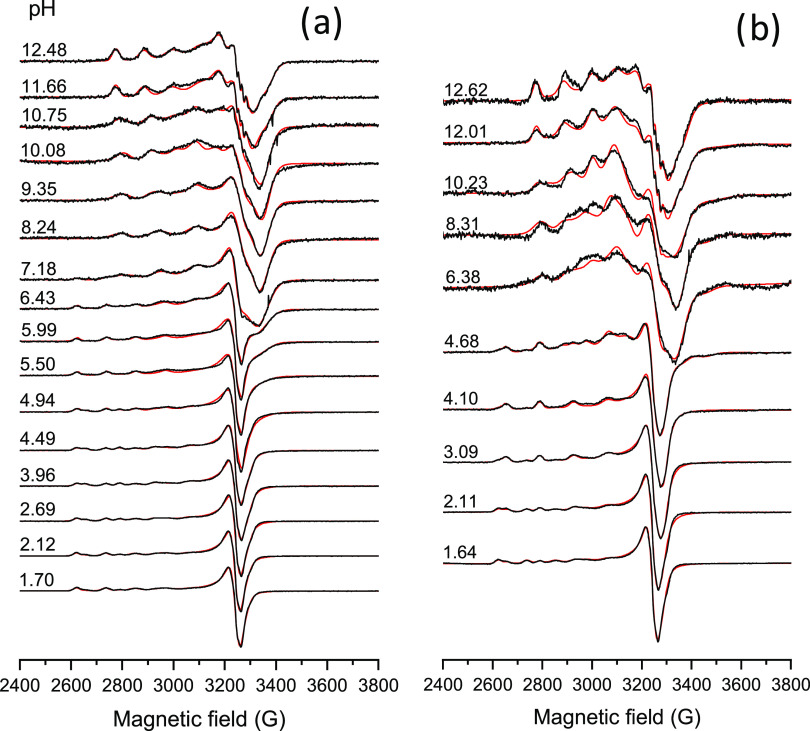
pH-dependent experimental (black) and simulated (red) frozen solution (77 K) EPR spectra recorded for the Cu(II)–**L3** system at concentrations (a) *c*_L_ = 0.25 mM, *c*_Cu_ = 0.25 mM, and (b) *c*_L_ = 0.23 mM, *c*_Cu_ = 0.50 mM in 30% (v/v) DMSO/H_2_O. The spectral intensity was normalized.

Additionally, EPR spectroscopic titrations were also performed for the Cu(II)–**L2** and **L3** systems, which revealed a more complicated speciation at 77 K in 30% (v/v) DMSO/H_2_O (notably, the presence of the cosolvent DMSO was required to provide the higher concentration required in this method). The more complex overview is most likely the consequence of the low temperature, which favors the formation of protonated complexes and dimerization processes. Representative spectra are shown for the Cu(II)–**L3** system at 1:1 and 1:2 metal-to-ligand ratios in Figure 9. In both systems, six components were taken into account to describe the measured spectra, besides the Cu(II) aqua complex. The obtained component spectra are compared in SI Figure S16, and their EPR parameters are collected in Table S5. Among them, component 2 was identified as the monoligand complex, in which the ligand coordinates via the (N,O^–^) donor set based on the similarities of the EPR parameters of a Cu(II) complex of a related 8HQ,^14^ while the EPR parameters of component 6 were similar to those of the isolated bis-complexes dissolved in DMSO (Table S5). A description of the other components is found in the Supporting Information section. Interestingly, in both studied systems, the intensity of the pH-dependent EPR spectra showed a significant decrease in certain ranges (Figure 9). In equimolar solution, the decrease of the second integral of the spectra is significant above pH ∼ 6 and at 2-fold ligand excess already above pH ∼ 2. This phenomenon suggests oligomerization/dimerization in these regions, which hides part of the signal in the baseline. The X-ray crystallographic analysis also showed the formation of dimeric species with **L1** and **L2** in the solid state, similarly to what was reported for related complexes.^14^

### 3.8 Determination of the Distribution Coefficient (*D*_7.40_) and Thermodynamic Solubility (*S*_7.40_)

The thermodynamic solubility (*S*) and the distribution coefficient (*D*) for the ligands and the isolated complexes were determined in water at pH 7.4 using UV–vis spectrophotometry for the analysis (Table 3). For the determination of *S* values, 24 h of incubation times was used. No reliable data are provided for the free ligands due to their partial decomposition, whereas the waiting time was only 3 h in the *n*-octanol/H_2_O partitioning experiments. The complexes have moderate aqueous solubility (*S* ∼ 0.7–1.8 mM) except **Zn(L3)**_**2**_, which is lower. The free ligands possess a moderate lipophilic character, while their complexes are more lipophilic.

**Table 3:** Thermodynamic Solubility (*S*_7.4_) of the Studied Free Ligands and Complexes Determined by UV–Vis Spectrophotometry at 37 °C and Their Distribution Coefficients (log *D*_7.4_) at pH 7.40 Determined Experimentally via *n*-Octanol/Water Partitioning at 25 °C

	log *D*_7.4_	*S*_7.4_ (μM)
**L1**	+0.63 ± 0.10	n.d.
**L2**	+0.64 ± 0.06	n.d.
**L3**	+0.76 ± 0.09	n.d.
**Cu**_**2**_**(L1)**_**4**_	>1.5	1221
**Cu**_**2**_**(L2)**_**4**_	+1.48 ± 0.07	1675
**Cu(L3)**_**2**_	>1.5	1821
**Zn(L1)**_**2**_	>1.5	694
**Zn(L2)**_**2**_	+0.86 ± 0.09	1674
**Zn(L3)**_**2**_	>1.5	87

### 3.9 Evaluation of the Interaction with BSA

Bovine serum albumin (BSA) is commonly used as a model protein for the evaluation of the interactions between bioactive compounds and human serum albumin (HSA) due to its lower cost, availability, and high homology to HSA. In fact, BSA shares 76% of its sequence with HSA and contains two tryptophan (Trp) amino acid residues, located in domains I and II: Trp-134 and Trp-212.^35^ HSA is the most abundant serum protein and a carrier of many drugs in plasma. Therefore, binding to HSA will impact the drug’s pharmacological profile since it may prolong its metabolization and induce a longer action time. BSA is also used as a supplement in mammalian cell cultures of *in vitro* tests due to the addition of fetal bovine serum; moreover, the formation of albumin–drug adducts is known to increase the solubility and bioavailability of several drugs.^36^^37^

First, we evaluated the impact of the presence of BSA on the stability of the complexes. Equimolar solutions of complexes and BSA were prepared in 5%DMSO:HEPES (10 mM, pH 7.4) and monitored over time with UV–vis spectroscopy. These studies are included in Figure S17, and here we present only the main observations.

The Zn(II) complexes present low solubility in aqueous media, as shown above, precipitating heavily in concentrations between 50 and 120 μM in 5% (v/v) DMSO/HEPES buffer. In the presence of equimolar amounts of BSA, two isosbestic points can be observed in the UV–vis spectra of the Zn(II) complexes, meaning that there is an equilibrium occurring between two products, probably the free Zn complex and Zn complex:BSA adducts. The gradual increase in absorbance at ca. 460 nm throughout the 24 h period indicates that this equilibrium is slow and takes at least 6 h to be completed, which contrasts with the complex precipitation in the absence of the protein. For the Cu(II) complexes, only very small changes were observed for 24 consecutive hours by UV–vis absorption spectroscopy.

Circular dichroism (CD) is a spectrophotometric technique widely used to study the affinity and binding modes of small molecules to proteins, such as BSA. When a CD “silent” compound binds the protein, induced CD bands can be observed at wavelengths of electronic transitions of the bound molecules.^38^

**Figure 10:**
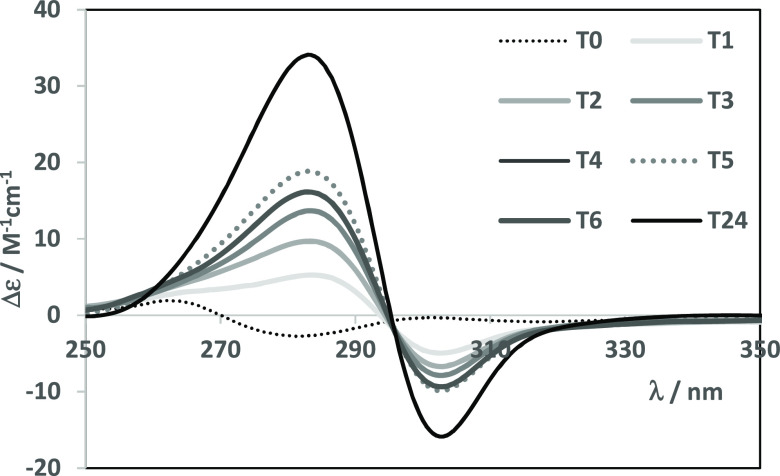
Circular dichroism spectra of solutions containing BSA (20 μM) and **Zn(L1)**_**2**_ (1:1) measured with time (indicated in hours in the legend).

The circular dichroism spectra of BSA–Zn(II) complex (1:1 ratio, ca. 20 μM) solutions show the appearance of induced CD bands due to the interaction. While the spectrum at time zero is weak and presents bands at 260 (+) and 282 (−) nm (see Figures 10 and S18), distinct bands show up after 1 h, with maxima at 284 (+) and 303 (−) nm and an isosbestic point at 296 nm, whose intensity increases with time. Moreover, under the same conditions, the free ligands alone or ZnCl_2_ (both in the presence of BSA) show no CD bands even after 24 h. Since the Zn(II) complexes are not optically active and BSA does not present absorption bands in this wavelength range, the observation of CD bands is clear proof of binding of the complexes close to chiral moieties of BSA. Data suggest that the Zn complexes might be bound to the protein via coordination bonds, as generally when the binding is by intermolecular binding modes, the interaction is fast and the induced CD signals are weaker. At least two binding sites exist, one initial with lower chiral induction and a final one that takes hours to develop, suggesting covalent binding of the metal ion to the protein. The UV–vis spectra also indicated that binding takes place, with isosbestic points clearly present. Zinc has a preference for octahedral geometries, and in this case, they may involve the protein and two bidentate ligand molecules. The overall process is kinetically slow as the bands take time to develop.

For the Cu(II) complexes, weak bands develop in the visible wavelength region (at ca. 600–700 nm) for solutions containing 50 μM BSA and 1:1 molar ratios (data not shown), confirming the binding of the complexes to BSA. Due to the low intensity of d–d bands, much higher concentrations (or path lengths) should be used to further analyze the binding with this technique in this spectral region. But clearly, the process is different from the Zn-complex behavior.

Fluorescence titrations of BSA with the complexes were carried out to evaluate if the compounds may bind near Trp-212 since this residue is buried inside a hydrophobic pocket in subdomain IIA and is sensitive to changes in its surroundings. Exposure of this residue to solvent molecules leads to quenching of its fluorescence upon excitation in the UV (295 nm). Only the Cu(II) complexes were studied since the Zn(II) complexes do not show enough stability in aqueous solution for a clear interpretation of the spectroscopic data at the low concentrations used.

**Figure 11:**
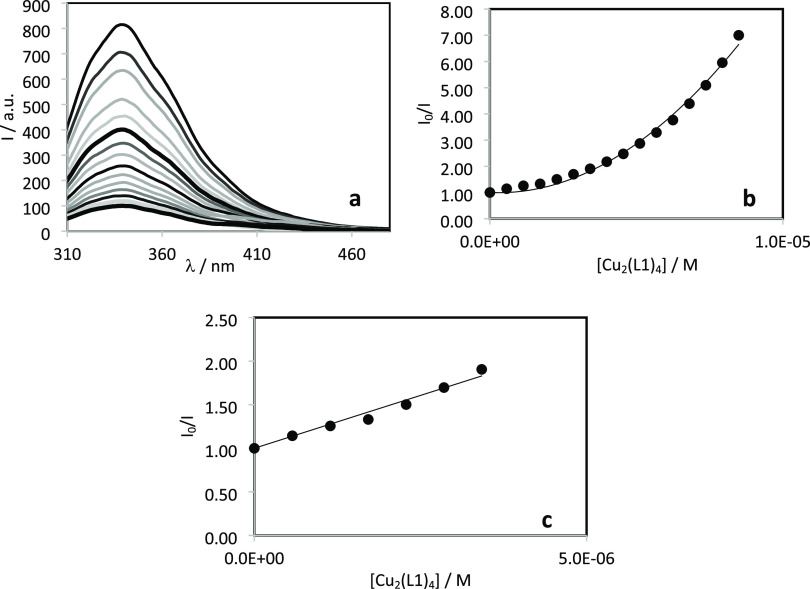
(a) Fluorescence emission (*I*) titration of BSA (0.47 μM) with **Cu**_**2**_**(L1)**_**4**_ (λ_exc_ = 295 nm). (b) Fitting of *I*_0_/*I* (λ_em_ = 340 nm) vs [**Cu**_**2**_**(L1)**_**4**_] with a second-order equation [*I*_0_/*I* = 80*x*^2^ – 4.7 × 10^4^*x* + 1, *R*^2^ = 0.993]. (c) Fitting of *I*_0_/*I* (λ_em_ = 340 nm) vs [**Cu**_**2**_**(L1)**_**4**_] according to the Stern–Volmer equation [*I*_0_/*I* = 2.5 × 10^5^*x* + 1, *R*^2^ = 0.977].

The titrations were carried out as detailed in the Experimental Section, and Figure 11 shows the fluorescence quenching data for **Cu**_**2**_**(L1)**_**4**_ (see Figure S19 for the other Cu(II) complexes). At [complex]:[BSA] ∼ 10, the quenching percentage was 68, 84, and 48% for **Cu**_**2**_**(L1)**_**4**_, **Cu**_**2**_**(L2)**_**4**_, and **Cu(L3)**_**2**_, respectively. All Stern–Volmer plots (*I*_0_/*I* vs [*Q*]) clearly show an upward curvature (Figures 11 and S19) and were fitted with a second-order equation, indicating that combined static and dynamic quenching processes occur. Note that while [*Q*] in the original approach indicates the equilibrium concentration of a free quencher, herein total concentrations were used assuming that [*Q*] and *c*_Q_ are only somewhat different in the case of moderate/weak binding and at relatively high *Q* excess. To obtain the dynamic quenching constant, fluorescence lifetime studies would be necessary, but these are outside the scope of the work; thus, the *K*_SV_ constants were simply obtained by fitting the quenching data in the lower range of quencher concentrations in which linearity is observed (see Table 4).

**Table 4:** Florescence Quenching Parameters: % Quenching at Molar Ratio Complex:BSA = 10; Stern–Volmer Quenching Constant and *R*^2^ Coefficient

complex	% quenching	*K*_SV_/M^–1^	*R*^2^
**Cu**_**2**_**(L1)**_**4**_	68	(2.5 ± 0.2) × 10^5^	0.977
**Cu**_**2**_**(L2)**_**4**_	84	(1.46 ± 0.05) × 10^5^	0.993
**Cu(L3)**_**2**_	48	(2.20 ± 0.07) × 10^5^	0.991

All Cu(II) complexes present Stern–Volmer constants in the 10^5^ range, with **Cu**_**2**_**(L1)**_**4**_ being the one that shows the strongest quenching ability. Thus, the presence of the piperidine moiety seems to have the highest impact on the binding to albumin. The presence of a longer linker between the imine and the morpholine also seems to increase the binding ability of the Cu(II) complex to BSA. The *K*_SV_ constants are within the range of reversible binding. A rough determination of the binding constant using linearization procedures (data not shown) yields values above 10^6^ M^–1^. Since for the Cu complexes the kinetics of protein binding is fast and the binding affinity is very high, different binding modes may be involved, and covalent binding cannot be excluded. Additional studies, outside the scope of this work, could yield a more conclusive picture.

Given these results, we can conclude that BSA in the cell incubation media of *in vitro* experiments, besides maintaining the integrity of the complexes in solution, is expected to promote their uptake by cells containing albumin receptors. We may also anticipate that the complexes may also bind HSA, allowing their transport in blood.

### 3.10 Redox Properties

**Figure 12:**
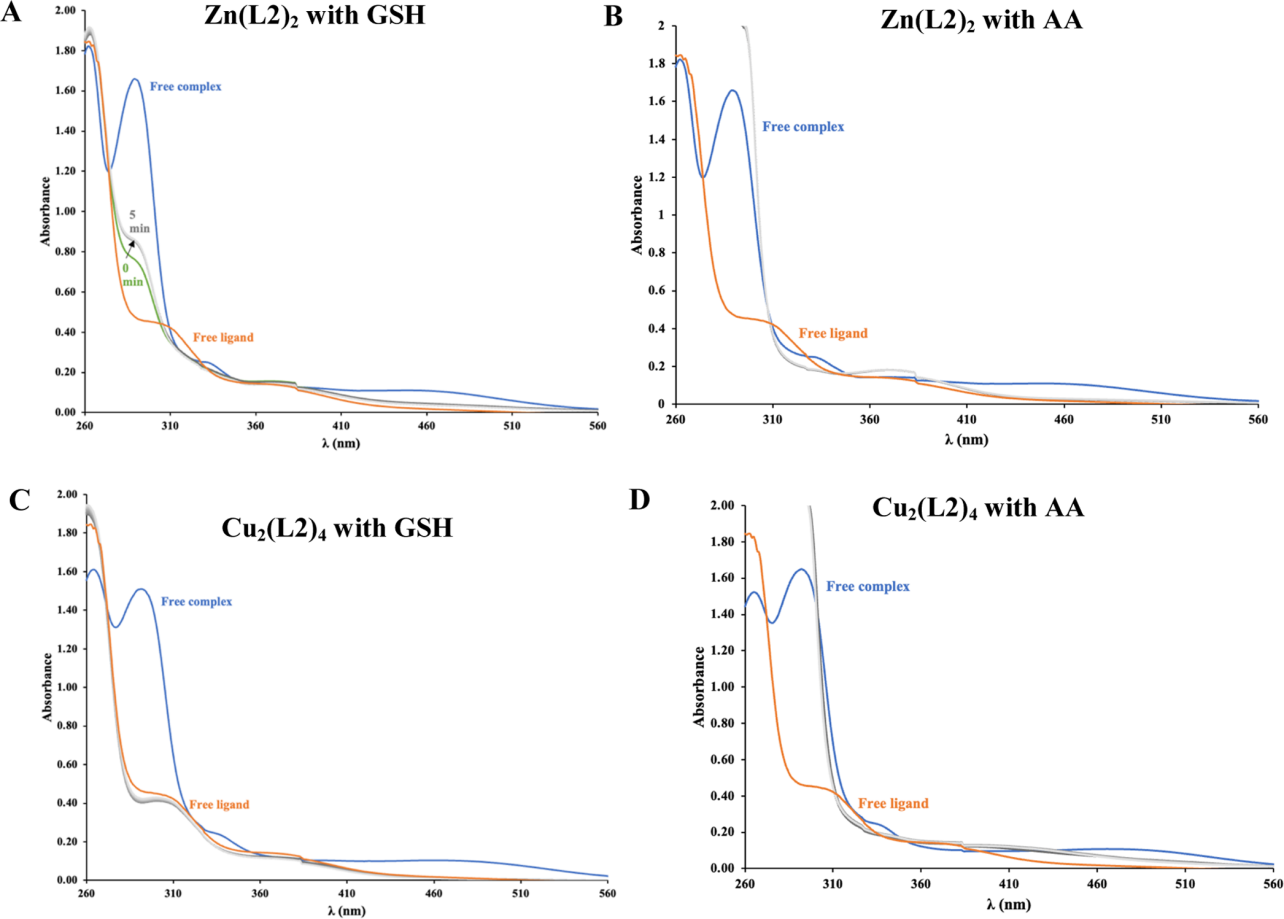
Time-dependent changes of the UV–vis spectra of **Zn(L2)**_**2**_ (A, B) and **Cu**_**2**_**(L2)**_**4**_ (C, D) complexes (55 μM) in the presence of 55 equiv of GSH and ascorbic acid (AA) (3 mM) at pH 7.4 in 5% (v/v) DMSO/H_2_O under anaerobic conditions. The spectra of the free ligand and free metal complexes are included for comparison.

As a preliminary study, we investigated the potential of selected zinc(II)–**Zn(L2)**_**2**_ and copper(II)–**Cu**_**2**_**(L2)**_**4**_ complexes to undergo intracellular reactions with biologically relevant reducing agents, particularly glutathione (GSH) and ascorbic acid (AA), by UV–vis absorption. After the addition of both reducing agents to the Zn(II) and Cu(II) complexes, significant spectral changes were observed (Figure 12). The reactions reached equilibrium on the first measurement (5 min), except for **Zn(L2)**_**2**_ in the presence of GSH, where an intermediate step was found at 5 min, meaning that the kinetics for this complex with GSH is slightly slower than for the other tested conditions and Cu complex.

Interestingly, only in the case of **Cu**_**2**_**(L**_**2**_**)**_**4**_ with GSH, liberation of the free ligand was observed, most probably involving a redox reaction between the complex and GSH and consequent formation of free Cu(I). For the other conditions, instead of the spectrum of free ligand, a new spectrum was obtained, possibly ascribed to the formation of ternary complexes with the reducing agents, as previously reported.^39^ Clearly, these complexes may induce changes in the redox state of the cell, simply by binding cellular reductants such as GSH and changing its free concentration or in the case of the Cu complexes through involvement in Fenton reactions after the formation of free Cu(I).

### 3.11 Assessment of the Antiproliferative Activity in Melanoma Cells

To assess the antiproliferative activities of the free ligands and complexes, a human melanoma cell line A375 was used. The effect of the free ligands and the complexes was evaluated after 48 h of incubation with a luminescence-based method. The IC_50_ values were calculated from dose–response curves (Figure S20) using the GraphPad Prism software, and the results are shown in Table 5.

**Table 5:** *In Vitro* Antiproliferative Activity of Compounds **L1**–**L3**, **Zn(L1)**_**2**_–**Zn(L3)**_**2**_, and **Cu**_**2**_**(L1)**_**4**_, **Cu**_**2**_**(L2)**_**4**_, **and Cu(L3)**_**2**_ after 48 h of incubation, in the Melanoma Cell Line A375, Determined by a Luminescent Method^a^

compounds	IC_50_ (μM) ± SD
L1	15.2 ± 0.2
Zn(L1)_2_	9.1 ± 1.5
Cu_2_(L1)_4_	5.2 ± 0.4
L2	15.9 ± 0.1
Zn(L2)_2_	10.3 ± 1.6
Cu_2_(L2)_4_	7.7 ± 0.4
L3	25.8 ± 0.3
Zn(L3)_2_	9.3 ± 1.1
Cu(L3)_2_	6.7 ± 0.2
Cisplatin	10.9 ± 1.3

a Cisplatin is also included for comparison purposes (determined by the MTT method).^40^ Values are mean ± SD of two independent biological experiments with 4 technical replicates each.

In general, all compounds demonstrated significant antiproliferative activity, with the Cu(II) compounds consistently more active than the Zn(II) complexes. Furthermore, the Cu(II) complexes are ca. 2-fold more cytotoxic than cisplatin (measured with the MTT assay^40^). There is also a clear effect of metal complexation on the cytotoxic profile of the complexes, as they are more cytotoxic than the corresponding ligand precursors.

Comparing the influence of the piperidine or morpholine pendant arms in the cytotoxic activity, it appears that the piperidine moiety (**L1**) confers a slightly higher cytotoxic profile when compared with the morpholine congeners (**L2** and **L3**), although not statistically significant.

In addition, the free ligands and complexes were also tested in a nontumorigenic cell line HaCaT (Table S6) by the colorimetric MTT assay. The luminescent method used to determine the IC_50_ values in the A375 cell line allows a more accurate determination of cell viability compared to other methods, such as the colorimetric MTT, but globally the cytotoxicity is higher against the A375 cell line. Notwithstanding, the IC_50_ values determined by each method are of the same order of magnitude and all compounds showed relatively low selectivity toward cancer cells (selectivity indices in the range of 1.2–2.8; Table S6). Further studies to increase drug targeting by encapsulation in liposomes are ongoing.

### 3.12 Assessment of Cell Death by Guava Viacount Assay

To further assess the effects of selected compounds in A375 cells, **Zn(L2)**_**2**_ and **Cu**_**2**_**(L2)**_**4**_ complexes were analyzed with the ViaCount assay by flow cytometry after incubation with the melanoma cells. The selection was made based on the ongoing liposomal formulation studies for the Cu(II) complexes, which were the metal complexes that showed the best antiproliferative results. In fact, among all Cu(II) complexes, **Cu**_**2**_**(L2)**_**4**_ has the best incorporation efficiency in lipid nanoformulations and thus was selected for further studies, including *in vivo* studies (data not shown). For comparison purposes, the Zn(II) complex bearing the same ligand, **Zn(L2)**_**2**_, was also selected for the Guava Viacount assay.

A positive control, dacarbazine (DTIC), a drug used in the clinic for the treatment of melanoma, was also included in this *in vitro* assay. The assay allows us to distinguish between viable and nonviable cells based on different permeabilities of two DNA-binding dyes that constitute the Guava ViaCount reagent.

**Figure 13:**
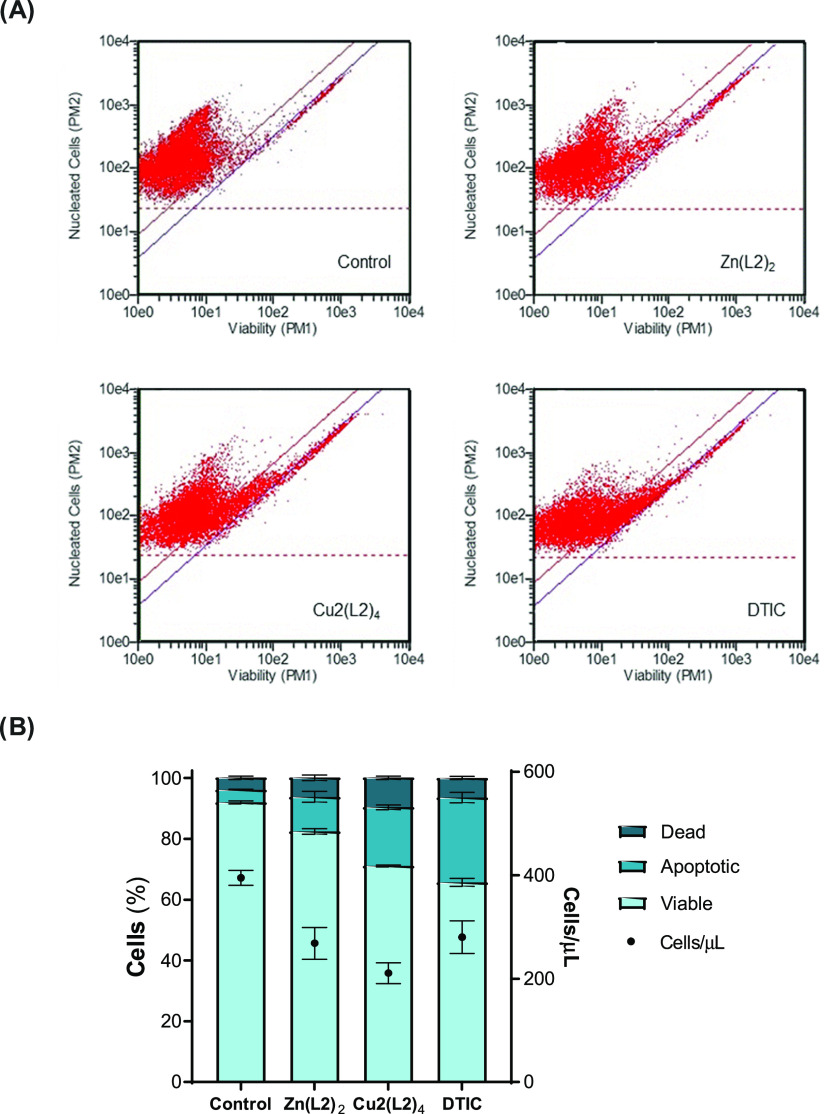
Evaluation of **Zn(L2)**_**2**_, **Cu**_**2**_**(L2)**_**4**_, and dacarbazine (DTIC) effect on A375 cell viability by Guava ViaCount. (A) Cell populations obtained by the Guava ViaCount flow cytometry analysis 48 h after incubation with compounds under study at 15, 12, and 70 μM, respectively. (B) Percentage of viable, apoptotic, and dead cells, as well as the number of cells/μL. Results are expressed as mean ± SD of one experiment with three replicates per condition.

Data obtained demonstrate that for the control (melanoma cells in the presence of complete medium) the percentage of dead and apoptotic cells was around 4% (Figure 13), while for cells incubated with the Zn and Cu complexes, the corresponding values were 10 and 20%, respectively. Furthermore, a 2-fold lower cell concentration was achieved for the cells after incubation with the copper complex. Overall, this assay confirms the antiproliferative effect of the complexes and suggests that apoptosis is involved in the mode of cell death.

## 4 Conclusions

Showing a wide range of biological activities and being important ionophores, many 8-hydroxyquinoline-based compounds are considered privileged structures in medicinal chemistry. In this work, we explored the possibility of obtaining synergistic effects by the combination of 8HQ and morpholine or piperidine heterocycles in the same molecule. 8HQ-2-carbaldehyde was reacted with amines containing morpholine or piperidine moieties, and three distinct Schiff bases **L1**–**L3** were obtained and characterized. These were reacted with Cu(II) and Zn(II) salts, and six new complexes were isolated. All compounds were fully characterized by analytic, spectroscopic, and spectrometric techniques. Noteworthily, the Cu(II) and Zn(II) complexes were also characterized by single-crystal X-ray diffraction data analyses. Complexes **Zn(L1)**_**2**_, **Zn(L2)**_**2**_, and **Zn(L3)**_**2**_ are all monomeric six-coordinated Zn(II) centers in a distorted octahedral geometry, the monodeprotonated ligands being coordinated by an NNO arrangement of donor atoms. In contrast, two of the Cu(II) complexes, with **L1** and **L2**, crystallize as dinuclear entities. The Cu–Cu distances in the dinuclear complexes are of 3.146 and 3.171 Å for **Cu**_**2**_**(L1)**_**4**_ and **Cu**_**2**_**(L2)**_**4**_, respectively, the structures being stabilized by reasonably strong intramolecular hydrogen bonds. The molecular structure of **Cu(L3)**_**2**_ shows the monomeric Cu(II) ion coordinated by two bidentate **L3** ligands acting through the quinolone-N and the phenolate-O atoms.

All compounds present low solubility in water, but they are moderately soluble in DMSO; thus, most studies in solution were carried out either in DMSO or in media also containing DMSO. Their solution behavior and evaluation of the stability of both the free ligands and of complexes were done by UV–vis, EPR, or NMR spectroscopies in different solvent conditions and several time periods. In the concentration range of ca. 35–400 μM, all complexes displayed adequate stability in DMSO. However, as expected, the stability depends on the concentration and amount of water present in the media, lower concentrations of compounds, and higher amounts of water favoring hydrolysis of the compounds. The binding of the Schiff bases **L1**–**L3** to Cu(II) or Zn(II) improves their resistance to hydrolysis, and globally in aqueous media containing 5% (v/v) DMSO, the Cu(II) complexes showed good stability. The Zn(II) complexes, particularly **Zn(L1)**_**2**_ and **Zn(L2)**_**2**_ at ca. 100–120 μM, did not show signs of decomposition but partially precipitated, while **Zn(L3)**_**2**_ showed significant hydrolysis within a 24 h period.

The p*K*_a_ values of the ligand precursors and formation constants of the complexes were determined by UV–vis titrations. Depending on pH and ligand-to-metal molar ratios, both 1:1 and 2:1 (L:M) complexes form and the Cu(II) complexes display higher stability than the Zn(II) complexes. EPR measurements for the Cu(II) systems in the pH range of 2–12.5 allowed the establishment of binding modes. Solubility and lipophilicity were determined at pH 7.4. The free ligands possess a moderate lipophilic character, while their complexes are more lipophilic. The complexes display moderate aqueous solubility (*S* ∼ 0.7–1.8 mM) except **Zn(L3)**_**2**_, which is poorly soluble.

Evaluation of the binding of prospective drugs to albumins is relevant to predict if these proteins may transport them in blood and their bioavailability. For this purpose, we used fluorescence emission titrations and measured the CD spectra of solutions containing various concentrations of BSA and complexes. The prepared Cu(II) and Zn(II) compounds bind to BSA, and particularly for the latter complexes, their solubility significantly increased due to this binding. Noteworthily, CD experiments revealed that the binding of the Zn(II) complexes takes place in relatively slow processes (more than 24 h), yielding reasonably strong induced CD bands, suggesting coordination to albumin. The binding of the compounds to BSA is also relevant to properly understand *in vitro* cytotoxicity data,^41^ as BSA is present in a relevant concentration in the incubation media of mammalian cells and may take roles in the uptake of the complexes by cells.

The reaction of selected Zn and Cu complexes with relevant cell (GSH) and plasma (ascorbic acid) reductants was followed by UV–vis spectroscopy, showing the ability of both complexes to form ternary complexes with these molecules. With **Cu**_**2**_**(L2)**_**4**_ and GSH, the liberation of the free ligand indicates that probably Cu(II) was reduced to Cu(I). These redox processes may lead to Fenton chemistry and oxidative cell stress and need to be further studied.

Preliminary cytotoxicity evaluations were carried out in which the compounds were screened in A375 malignant melanoma cells, as well as a noncancerous cell line HaCaT, to evaluate their selectivity. Selectivity is low (SI values: 1.2–2.8), but all compounds demonstrated significant antiproliferative activity, the Cu(II) compounds being ca. 2-fold more cytotoxic than cisplatin, and are more active than the Zn(II) complexes. Noteworthily, the complexes are more cytotoxic than ligand precursors **L1**–**L3**, the piperidine moiety conferring a slightly higher cytotoxic profile when compared with the morpholine congeners. A Guava ViaCount assay corroborated the impact on melanoma cell viability, with the Cu complexes imposing a higher % of apoptotic and dead cells than the Zn complexes. The copper complex compares well with the positive control, dacarbazine, despite being used at a much lower concentration (12 vs 70 μM). Studies on the mode of cell death are being carried out, involving cell cycle effects, ROS generation, and cellular uptake.

Globally, the prepared Cu(II) and Zn(II) complexes have potential as anticancer agents but require improved drug targeting and selectivity characteristics. We are presently working in this field, namely, testing the encapsulation of the compounds in liposomes.

## Supporting Information Available

Data on ligands and complex characterization: ESI-MS characterization of the ligand precursors and corresponding metal complexes, ^1^H NMR of the ligands and Zn complexes in DMSO-*d*_6_, additional data on FTIR, UV–vis absorption and fluorescence spectroscopies, selected bond lengths (Å) and angles (°) of the metal complexes, and packing of the Cu complexes; stability studies in DMSO and aqueous media by ^1^H NMR and UV–vis spectroscopies; component EPR spectra and their anisotropic EPR parameters obtained from the simulation of frozen solution EPR spectra; BSA interaction studies by UV–vis, circular dichroism, and fluorescence spectroscopies; dose–response curves of the free ligands and complexes in A375 cells; and *in vitro* antiproliferative activity of the compounds in nontumorigenic HaCaT cells (PDF)
