# Supplementary material for: Cu(II) and Zn(II) Complexes of New 8-Hydroxyquinoline Schiff Bases: Investigating Their Structure, Solution Speciation, and Anticancer Potential
Source: Inorg Chem. 2023 Jul 13;62(29):11466–86. doi: 10.1021/acs.inorgchem.3c01066 (PMC10369496; doi:10.1021/acs.inorgchem.3c01066)
Supplement: Supplementary file 1 — ic3c01066_si_001.pdf [file ic3c01066_si_001.pdf]

Supporting Information

Cu(II) and Zn(II) Complexes of New 8-Hydroxyquinoline Schiff Bases: Investigating Their Structure, Solution Speciation and Anticancer Potential

Leonor Côrte-Real[a], Vivien Pósa[b], Matilde Martins[a†], Raquel Colucas[a†], Nóra V. May[c], Xavier Fontrodona[d], Isabel Romero[d], Filipa Mendes[e], Catarina Pinto Reis[f,g], Maria Manuela Gaspar[f], João Costa Pessoa[a], Éva A. Enyedy[b*], Isabel Correia [a*]

[a]Centro de Química Estrutural, Institute of Molecular Sciences, and Department of Chemical Engineering, Instituto Superior Técnico, Universidade de Lisboa, Avenida Rovisco Pais 1, 1049-001 Lisboa, Portugal

[b]MTA-SZTE Lendület Functional Metal Complexes Research Group, Department of Inorganic and Analytical Chemistry, Interdisciplinary Excellence Centre, University of Szeged, Dóm tér 7, H-6720 Szeged, Hungary

[c]Centre for Structural Science, Research Centre for Natural Sciences, Eötvös Loránd Research Network, Magyar Tudósok krt. 2, H-1117 Budapest, Hungary

[d]Departament de Química and Serveis Tècnics de Recerca, Universitat de Girona, Campus de Montilivi, E-17071 Girona, Spain

[e]Centro de Ciências e Tecnologias Nucleares and Department of Nuclear Sciences and Engineering, Instituto Superior Técnico, Universidade de Lisboa, Estrada Nacional 10 (km139,7), 2695-066 Bobadela LRS, Portugal

[f]Research Institute for Medicines (iMed.ULisboa), Faculty of Pharmacy, Universidade de Lisboa, 1649-003 Lisboa, Portugal

[g]Instituto de Biofísica e Engenharia Biomédica, Faculdade de Ciências, Universidade de Lisboa, 1749-016 Lisboa, Portugal

*Correspondence: icorreia@tecnico.ulisboa.pt and enyedy@chem.u-szeged.hu

†These authors contributed equally to this work

Table S1 – ESI-MS characterization of the ligand precursors and corresponding metal complexes in positive and negative mode. FA: formic acid.

Metal	Ligand	Species	Calcd (*m/z*)	Found (*m/z*)
-	**L1**	$[M+H]^+$	322.13	322.12
		$[2M+H]^+$	643.25	642.95
		$[2M+K]^+$	681.20	680.99
-	**L2**	$[M+H]^+$	324.11	324.03
		$[2M+H]^+$	647.21	646.75
		$[2M+K]^+$	685.16	684.90
-	**L3**	$[M+H]^+$	338.13	338.05
		$[2M+H]^+$	675.25	674.73
		$[2M+K]^+$	713.20	712.95
**Zn(II)**	**L1**	$[M+H]^+$	629.26	629.11
		$[M+FA-H]^-$	673.25	673.30
	**L2**	$[M+H]^+$	633.22	633.14
		$[M+FA-H]^-$	677.21	677.63
	**L3**	$[M+H]^+$	661.25	661.03
		$[M+FA-H]^-$	705.24	705.20
**Cu(II)**	**L1**	$[M+H]^+$	628.26	627.97
		$[M+FA-H]^-$	672.25	673.44
	**L2**	$[M+H]^+$	632.22	631.95
		$[M+FA-H]^-$	676.21	676.60
	**L3**	$[M+H]^+$	660.25	659.73
		$[M+FA-H]^-$	704.24	705.45

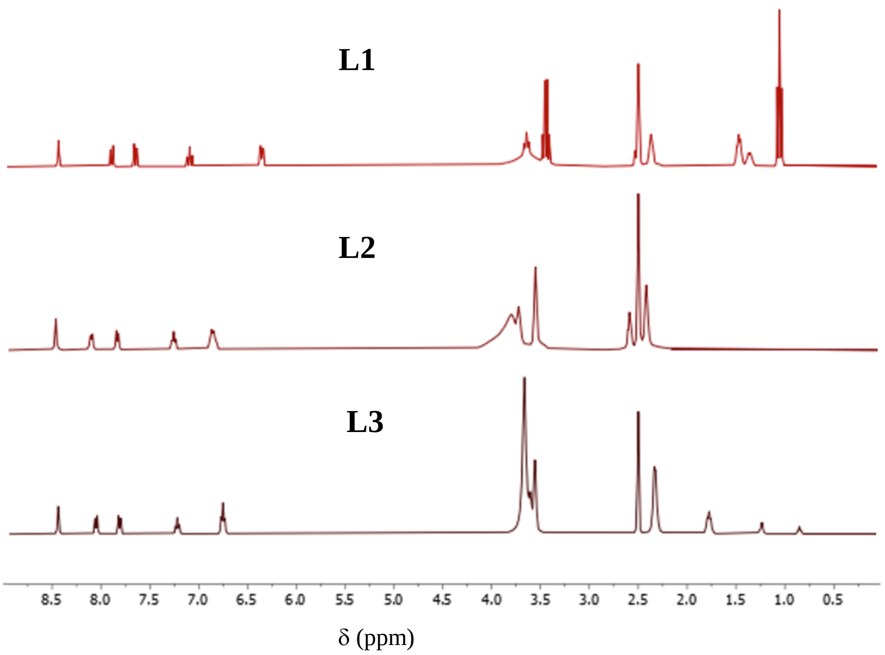

Figure S1 - ^{1}H NMR spectra of **L1**, **L2** and **L3** in DMSO-d_6 at room temperature (concentrations between 84 – 88 mM).

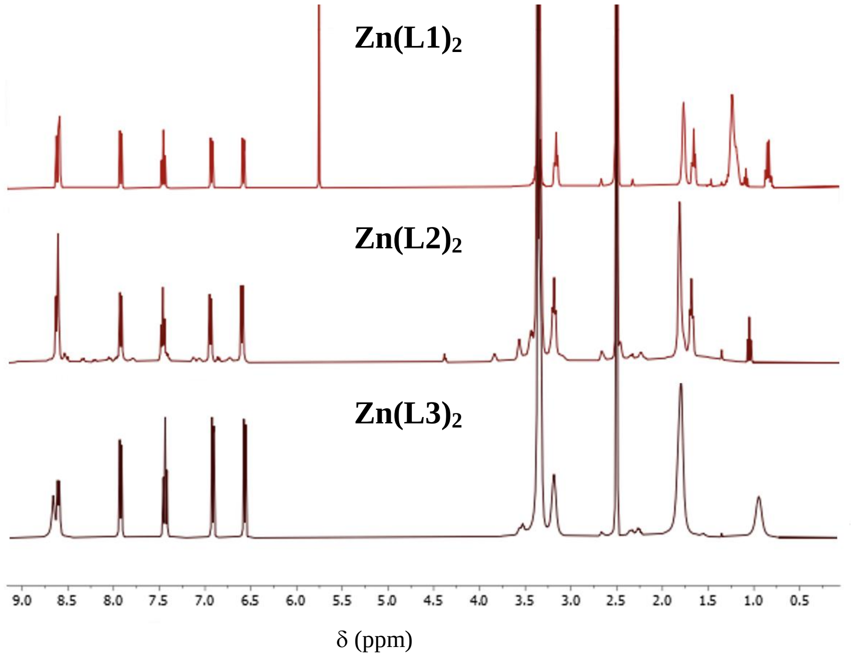

Figure S2 - ^{1}H NMR spectra of **Zn(L1)$_2$**, **Zn(L2)$_2$** and **Zn(L3)$_2$** in DMSO-d$_6$ at room temperature (concentrations between 57 and 60 mM).

Table S2 – Spectroscopic data of the free ligands and corresponding metal complexes in DMSO. (sh means shoulder).

Ligand	Metal	λ_{max} (nm)	ε ($M^{-1}cm^{-1}$)
**L1**	-	270, 314, 347	1.8×10^{4}, sh, 2.5×10^{3}
	Zn(II)	300, 342, 370, 497	4.6×10^{4}, sh, 3.2×10^{3}
	Cu(II)	268, 312, 422	3.8×10^{4}, sh, 2.4×10^{3}
**L2**	-	269, 314, 349	1.9×10^{4}, sh, 2.0×10^{3}
	Zn(II)	301, 342, 370, 497	4.2×10^{4}, sh, 2.9×10^{3}
	Cu(II)	297, 308, 422	5.3×10^{4}, sh, 3.4×10^{3}
**L3**	-	269, 314, 349	2.0×10^{4}, sh, 1.9×10^{3}
	Zn(II)	300, 342, 370, 498	4.3×10^{4}, sh, 3.2×10^{3}
	Cu(II)	270, 293, 418	3.9×10^{4}, sh, 2.4×10^{3}

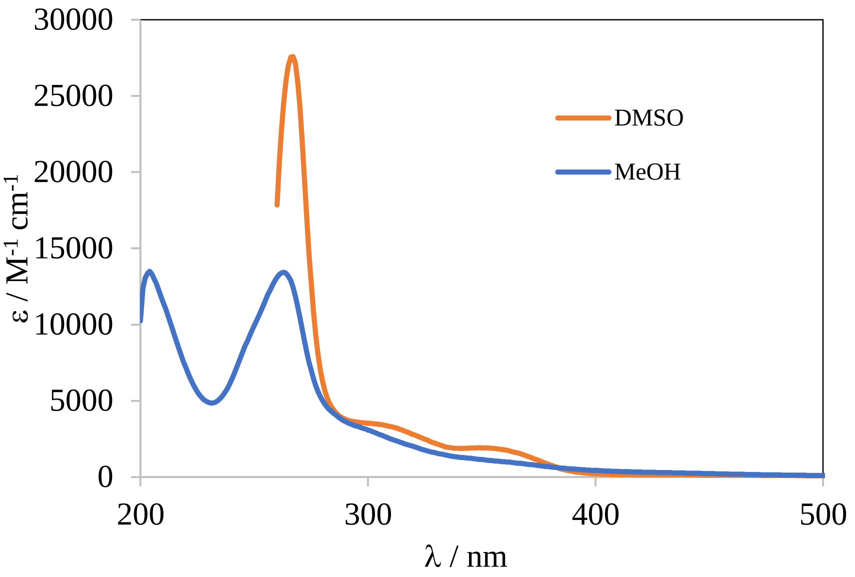

Figure S3 – UV-vis molar absorbance spectra of **L3** measured in DMSO (135 μM) and MeOH (112 μM).

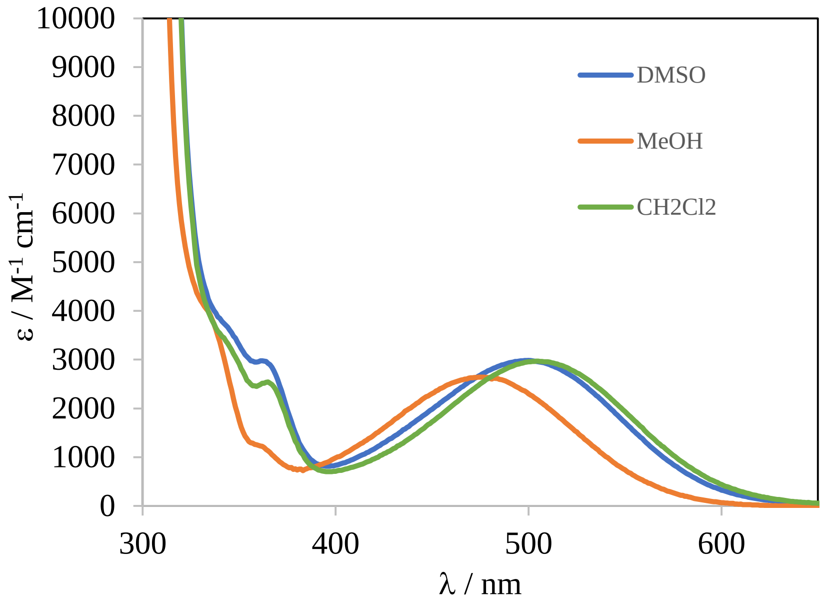

Figure S4 – UV-vis molar absorbance spectra of **Zn(L3)$_2$** measured in DMSO, CH_2Cl_2 and MeOH (ca. 15 μM).

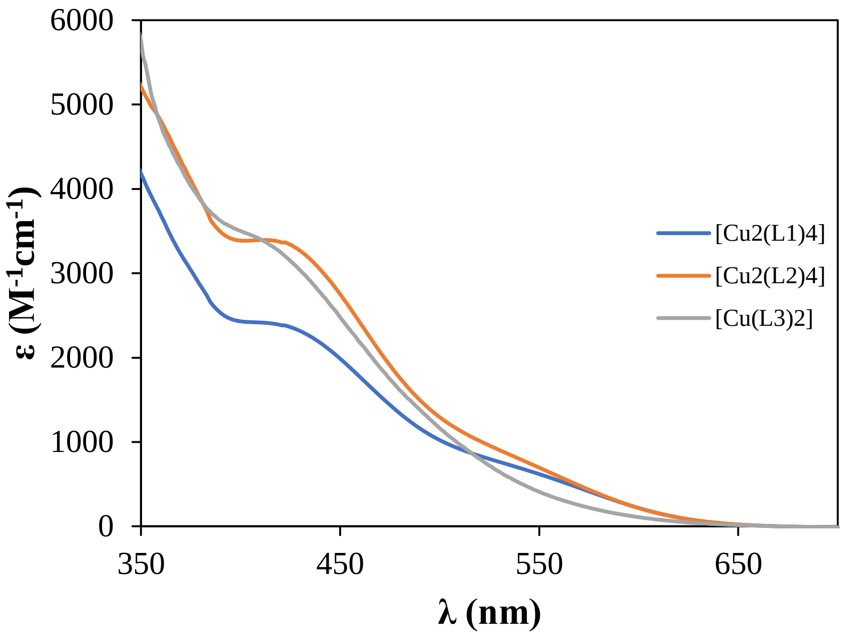

Figure S5 – UV-vis molar absorbance spectra of complexes **Cu_2(L1)$_4$**, **Cu_2(L2)$_4$** and **Cu(L3)$_2$** in DMSO (concentrations between 27-53 μM).

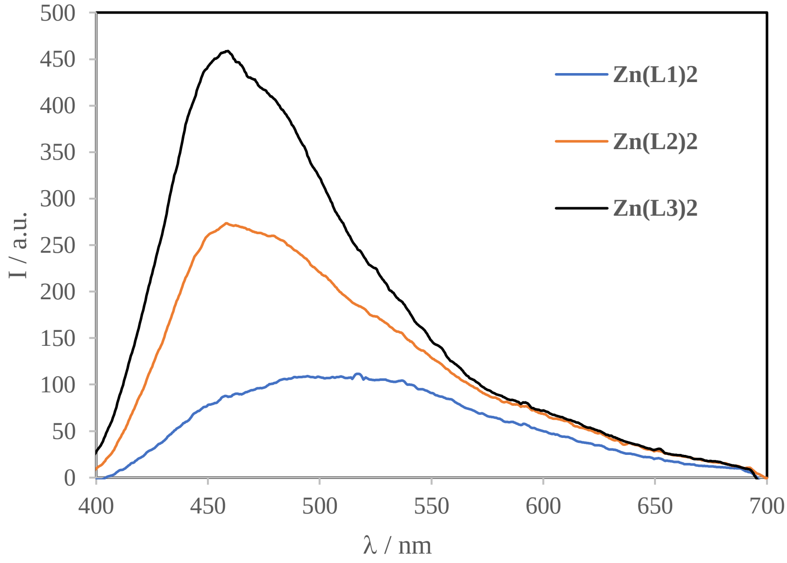

Figure S6 – Fluorescence emission spectra of complexes **Zn(L1)$_2$—Zn(L3)$_2$** (15 μM) in DMSO. λ_{exc} = 350 nm.

Table S3 - Selected bond lengths (Å) and angles (°) for of the **Zn(L1)$_2$**, **Zn(L2)$_2$**, **Zn(L3)$_2$**, **Cu$_2$(L1)$_4$**, **Cu$_2$(L2)$_4$** and **Cu(L3)$_2$** complexes.

**Zn(L1)$_2$**		**Zn(L2)$_2$**	
Zn(1)-N(5)	2.025(15)	Zn(1)-N(11)	2.020(4)
Zn(1)-O(2)	2.078(14)	Zn(1)-O(23)	2.091(14)
Zn(1)-N(35)	2.385(15)	Zn(1)-N(35)	2.374(5)
Zn(1)-N(26)	2.046(15)	Zn(1)-N(32)	2.025(4)
Zn(1)-O(23)	2.088(12)	Zn(1)-O(2)	2.095(4)
Zn(1)-N(14)	2.415(19)	Zn(1)-N(14)	2.401(5)
N(5)-Zn(1)-N(26)	161.9(6)	N(11)-Zn(1)-N(32)	164.89(18)
N(26)-Zn(1)-O(2)	115.3(5)	N(32)-Zn(1)-O(23)	79.71(16)
N(26)-Zn(1)-O(23)	79.8(6)	N(32)-Zn(1)-O(2)	109.39(15)
N(5)-Zn(1)-N(35)	98.6(6)	N(11)-Zn(1)-N(35)	95.30(17)
O(2)-Zn(1)-N(35)	98.0(5)	O(23)-Zn(1)-N(35)	151.66(15)
N(5)-Zn(1)-N(14)	71.1(7)	N(11)-Zn(1)-N(14)	72.35(17)
O(2)-Zn(1)-N(14)	151.6(6)	O(23)-Zn(1)-N(14)	94.11(17)
N(35)-Zn(1)-N(14)	84.5(6)	N(35)-Zn(1)-N(14)	85.71(17)
N(5)-Zn(1)-O(2)	80.6(6)	N(11)-Zn(1)-O(23)	111.68(16)
N(5)-Zn(1)-O(23)	107.5(5)	N(11)-Zn(1)-O(2)	79.63(16)
O(2)-Zn(1)-O(23)	98.0(5)	O(23)-Zn(1)-O(2)	98.00(15)
N(26)-Zn(1)-N(35)	71.7(6)	N(32)-Zn(1)-N(35)	72.26(17)
O(23)-Zn(1)-N(35)	151.2(6)	O(2)-Zn(1)-N(35)	95.13(15)
N(26)-Zn(1)-N(14)	92.4 (6)	N(32)-Zn(1)-N(14)	97.63(17)
O(23)-Zn(1)-N(14)	92.6(6)	O(2)-Zn(1)-N(14)	151.92(15)

**Zn(L3)$_2$**		**Cu$_2$(L1)$_4$**			
Zn(1)-N(11)	2.024(3)	Cu(1)-O(2)	1.886(5)	Cu(1A)-O(2A)	1.896(4)

Zn(1)-O(24)	2.045(3)	Cu(1)-N(5)	2.095(5)	Cu(1A)-N(5A)	2.105(5)
Zn(1)-N(14)	2.404(4)	Cu(1)-O(23A)	2.381(4)	Cu(1A)-O23A)	1.904(4)
Zn(1)-N(33)	2.031(3)	Cu(1)-O(23)	1.914(4)	Cu(1A)-N(27A)	2.107(4)
Zn(1)-O(2)	2.096(3)	Cu(1)-N(26)	2.122(5)		
Zn(1)-N(36)	2.465(5)	O(23)-Cu(1A)	2.360(4)		
N(11)-Zn(1)-N(33)	162.28(12)	O(2)-Cu(1)-O(23)	176.2(2)		
N(33)-Zn(1)-O(24)	80.19(12)	O(23)-Cu(1)-N(5)	98.7(2)		
N(33)-Zn(1)-O(2)	106.79(11)	O(23)- Cu (1)-N(26)	82.33(19)		
N(11)-Zn(1)-N(14)	72.61(12)	O(2)- Cu (1)-O(23A)	91.28(18)		
O(24)-Zn(1)-N(14)	88.70(13)	N(5)- Cu (1)-O(23A)	102.43(18)		
N(11)-Zn(1)-N(36)	91.821(15)	O(2)- Cu (1)-N(5)	84.12(2)		
O(24)-Zn(1)-N(36)	151.21(14)	O(2)- Cu (1)-N(26)	160.4(2)		
N(14)-Zn(1)-N(36)	90.62(16)	O(23)- Cu (1)-O(23A)	85.57(17)		
N(11)-Zn(1)-O(24)	115.30(11)	N(26)- Cu (1)-O(23A)	97.13(8)		
N(11)-Zn(1)-O(2)	76.62(12)	Cu(1)- O(23) -Cu(1A)	94.19(18)		
O(24)-Zn(1)-O(2)	101.21(12)	N(26)- Cu (1)-N(5)	160.4(2)		
N(33)-Zn(1)-N(14)	100.52(12)	O(2A)-Cu(1A)-O(23A)	176.4(2)		
O(2)-Zn(1)-N(14)	152.12(10)	O(23A)-Cu(1A)-N(5A)	95.7(2)		
N(33)-Zn(1)-N(36)	71.65 (15)	O(23A)- Cu (1A)-N(27A)	81.9(2)		
O(2)-Zn(1)-N(36)	92.67(13)	O(2A)- Cu (1A)-O(23)	90.14(18)		
		O(23)-Cu(1A)-N(5A)	93.27(18)		
		O(2A)-Cu(1A)-N(5A)	83.8(2)		
		O(2A)- Cu (1A)-O(27A)	99.5(2)		
		N(5A)- Cu (1A)-O(27A)	165.6(2)		
		O(23A)- Cu (1A)-O(23)	86.38(17)		
		N(27A)- Cu (1A)-O(23)	100.74(18)		
		Cu(1)- O(23A) -Cu(1A)	93.80(18)		

**$Cu(L3)_2$**		**$Cu_2(L2)_4$**			
Cu(1)-O(2)	1.8815(9)	Cu(1)-O(2)	1.910(3)	Cu(1A)-O(2)	2.322(3)

Cu(1)-N(5)	2.0866(11)	Cu(1)-N(11)	2.125(3)	Cu(1A)-N(11)	2.131(3)
		Cu(1)-O(23A)	2.404(3)	Cu(1A)-O23A)	1.905(3)
O(2)-Cu(1)-O(2)	180.00(5)	Cu(1)-O(23)	1.879(3)	Cu(1A)-N(32A)	2.131(3)
O(2)-Cu(1)-N(5)	83.94(4)	Cu(1)-N(32)	2.101(3)	O(2A)Cu(1A)	1.889(3)
O(2)-cu(1)-N(5)	96.06(4)				
N(5)-Cu(1)-N(5)	180	O(2)-Cu(1)-O(23)	175.13(11)		
		O(23)-Cu(1)-N(32)	83.01(12)		
		O(23)- Cu (1)-N(11)	97.33(12)		
		O(2)- Cu (1)-O(32)	99.57(12)		
		N(11)- Cu (1)-O(23A)	100.39(10)		
		O(2)- Cu (1)-N(11)	81.69(12)		
		O(23A)- Cu (1)-N(32)	99.78(11)		
		O(23)- Cu (1)-O(23A)	92.06(10)		
		O(2)- Cu (1)-O(23A)	83.45(10)		
		Cu(1)- O(2) -Cu(1A)	96.59 (11)		
		N(32)- Cu (1)-N(11)	159.80(12)		
		O(2A)-Cu(1A)-O(23A)	179.48(12)		
		O(23A)-Cu(1A)-N(32A)	81.70(12)		
		O(23A)- Cu (1A)-N(11a)	97.05(12)		
		O(2)- Cu (1A)-O(2A)	93.79(11)		
		N(11A)- Cu (1A)-O(2)	95.28(10)		
		O(2A)- Cu (1A)-N(11A)	82.64(12)		
		O(2A)- Cu (1A)-N(32A)	98.70(12)		
		O(2A)- Cu (1A)-O(32A)	98.70(12)		
		O(2)- Cu (1A)-O(23A)	85.82(10)		
		Cu(1A)- O(23A) -Cu(1)	94.07 (10)		
		N(32A- Cu (1)-N(11A)	167.03(12)		
		O(2)-Cu(1A)-N(32A)	97.50(11)		

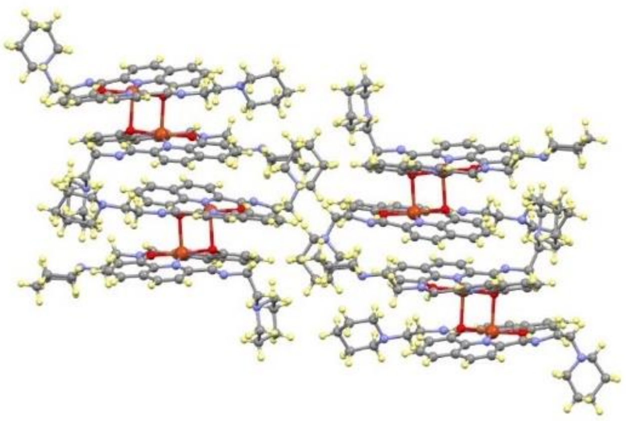

$Cu_2(L1)_4$

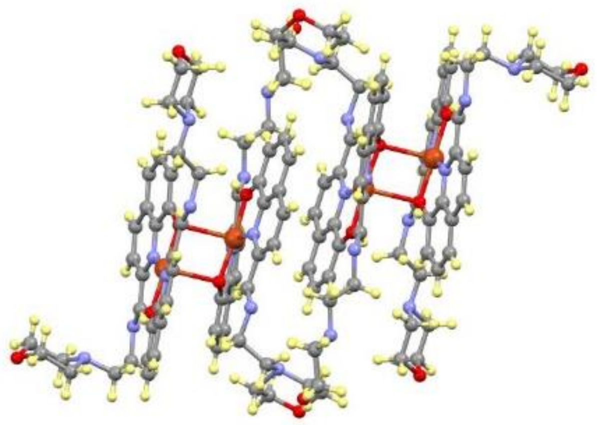

$Cu_2(L2)_4$

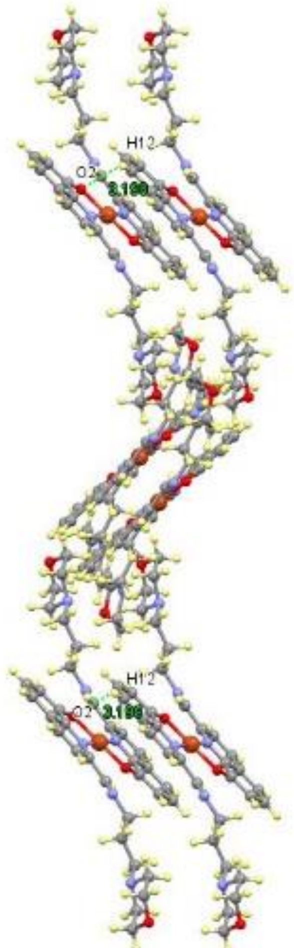

$Cu(L3)_2$

Figure S7 - Packing of the **$Cu_2(L1)_4$, $Cu_2(L2)_4$** and **$Cu(L3)_2$** complexes along the a axis.

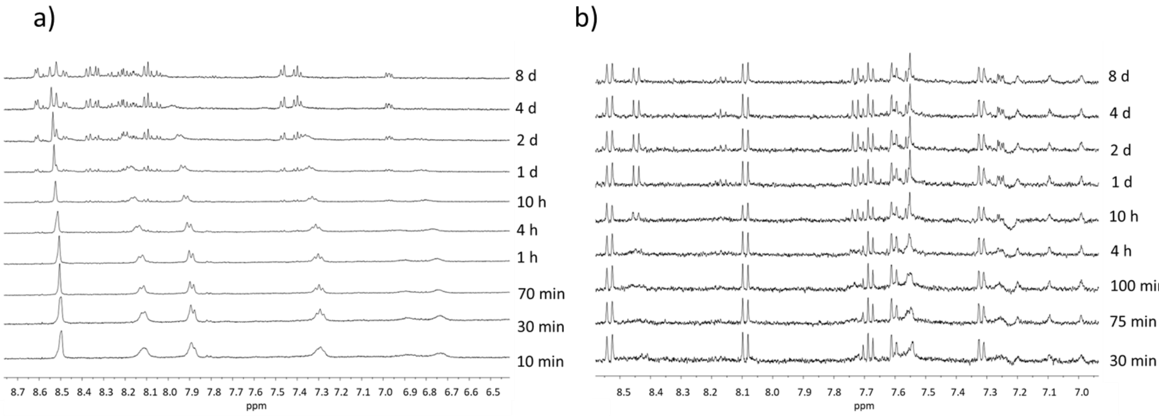

Figure S8 - ^{1}H NMR spectra of **L3** in a) DMSO-d_6 and b) 30% (v/v) DMSO-d_6/H_2O at pH ~ 4 followed for 8 days.

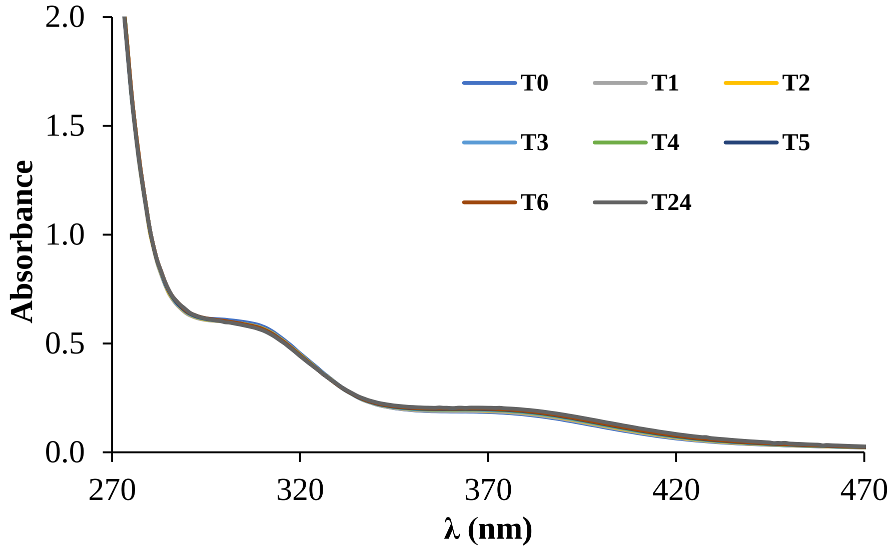

Figure S9 – UV-vis spectra of **L1** (228 μM) from time zero to the 24 h measurement in HEPES buffer (10 mM, pH 7.4) with 5% (v/v) DMSO.

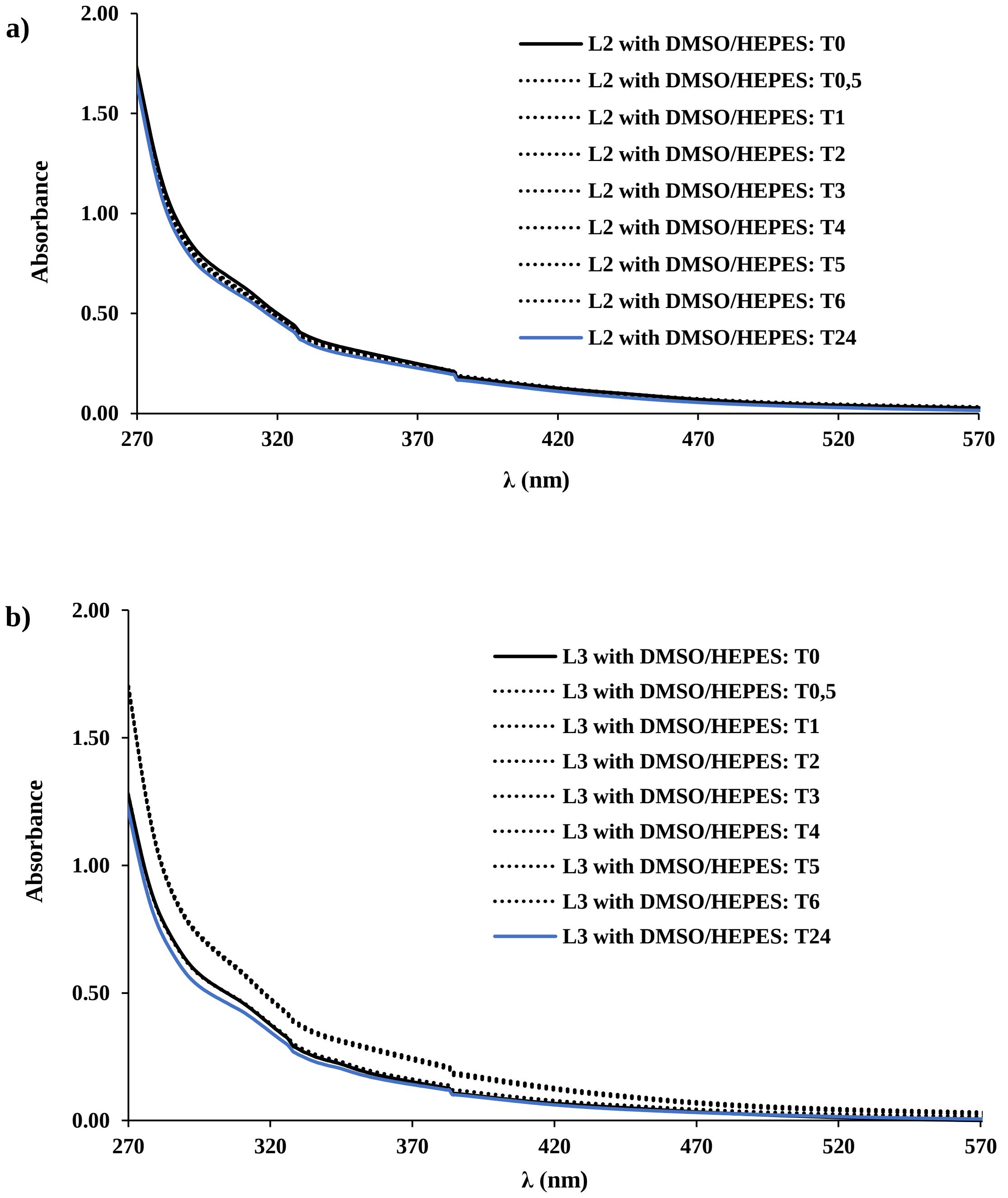

Figure S10 – UV-vis spectra of a) **L2** (147 μM) and b) **L3** (141 μM) from time zero to the 24 h measurement in HEPES buffer (10 mM, pH 7.4) with 5% (v/v) DMSO.

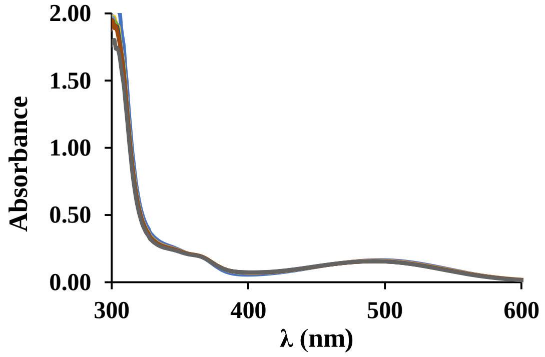

Zn(L1)$_2$ (75 µM)

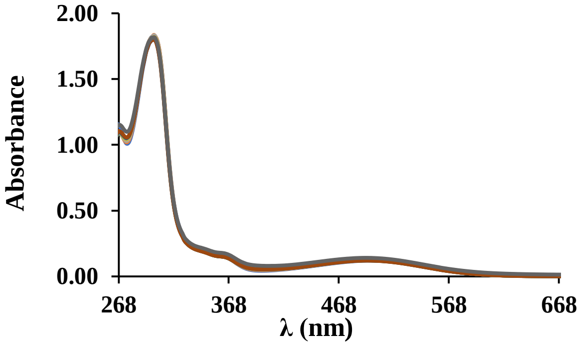

Zn(L2)$_2$ (45 µM)

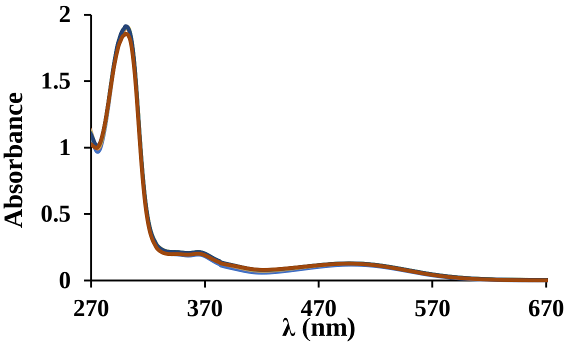

Zn(L3)$_2$ (45 µM)

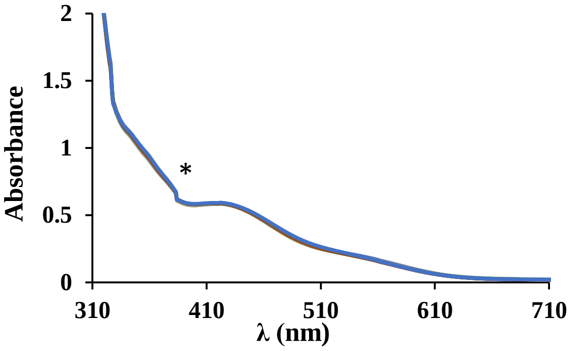

Cu$_2$(L1)$_4$ (255µM)

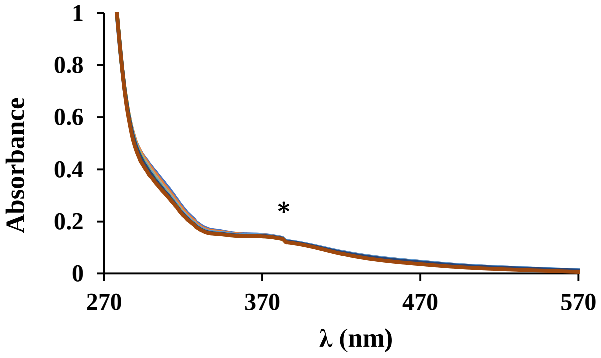

Cu$_2$(L2)$_4$ (35 µM)

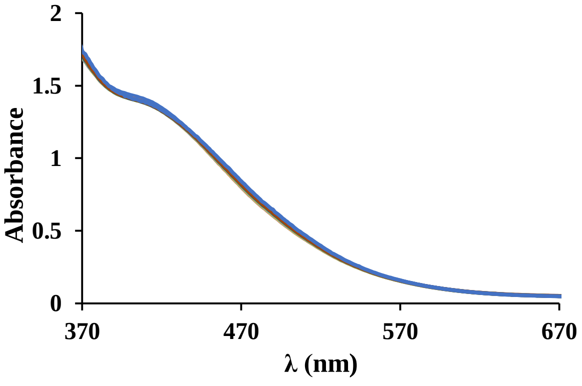

Cu(L3)$_2$ (375 µM)

Figure S11 – UV-vis spectra of the Zn(II) and Cu(II) complexes in DMSO from time zero to the 24 h measurement. Complex concentrations indicated in the figure. *Electronic artifact due to lamp change.

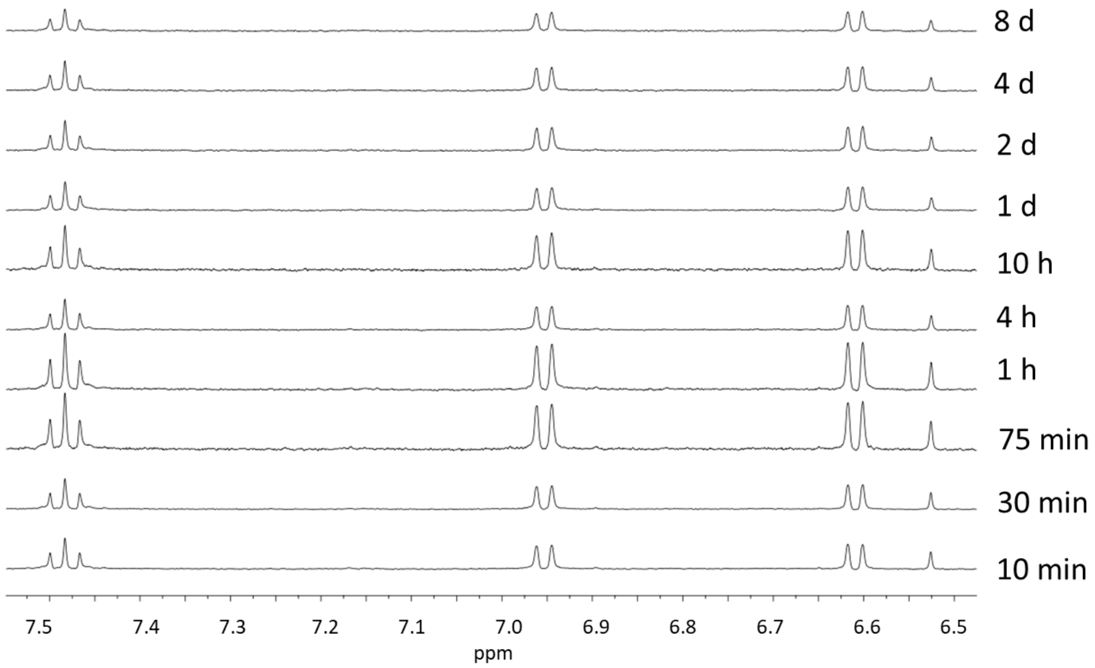

Figure S12 - ^{1}H NMR spectra of **Zn(L1)₂** in DMSO measured with time up to 8 days. *[c_L = 1 mM; t = 25 ˚C]*

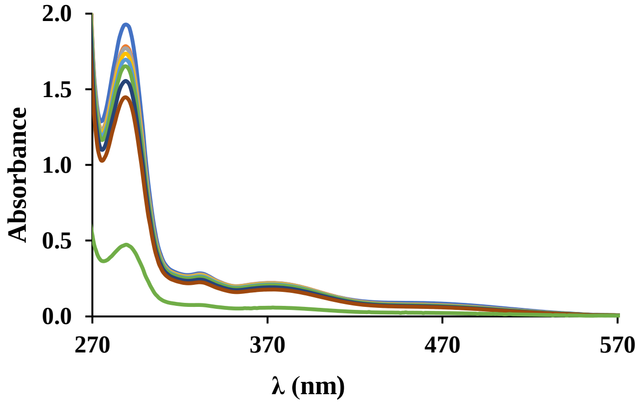

Zn(L1)$_2$ (100 μM)

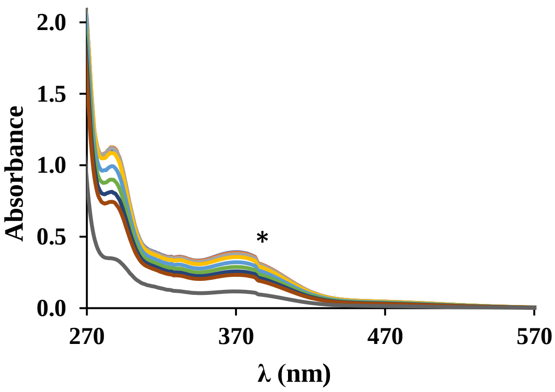

Zn(L2)$_2$ (122 μM)

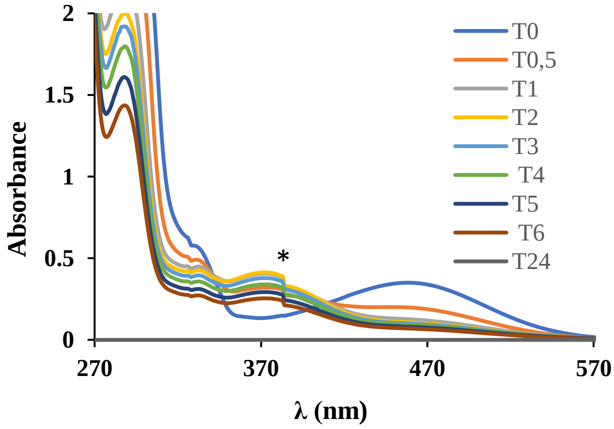

Zn(L3)$_2$ (135 μM)

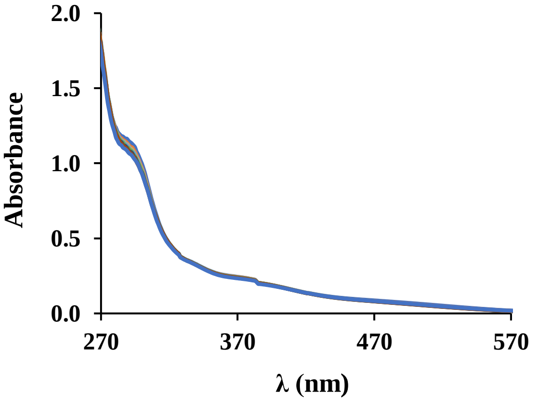

Cu$_2$(L1)$_4$ (52 μM)

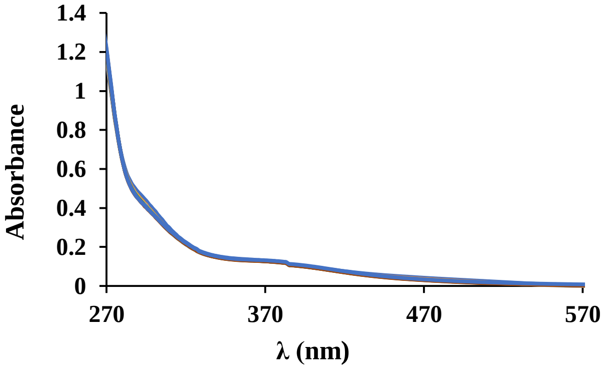

Cu$_2$(L2)$_4$ (35 μM)

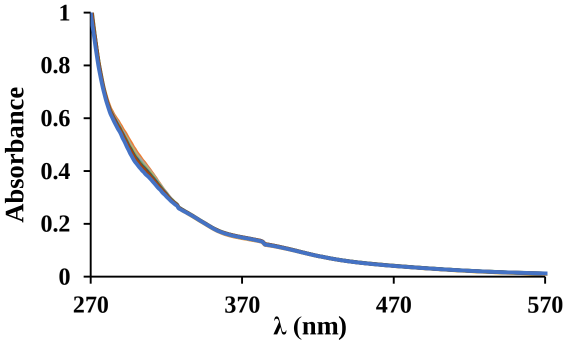

Cu(L3)$_2$ (35 μM)

Figure S13 – UV-vis spectra of the Zn(II) and Cu(II) complexes in HEPES buffer: DMSO 5% (v/v) from time zero to the 24 h measurement. Complex concentrations indicated in the figure. *Electronic artifact due to lamp change.

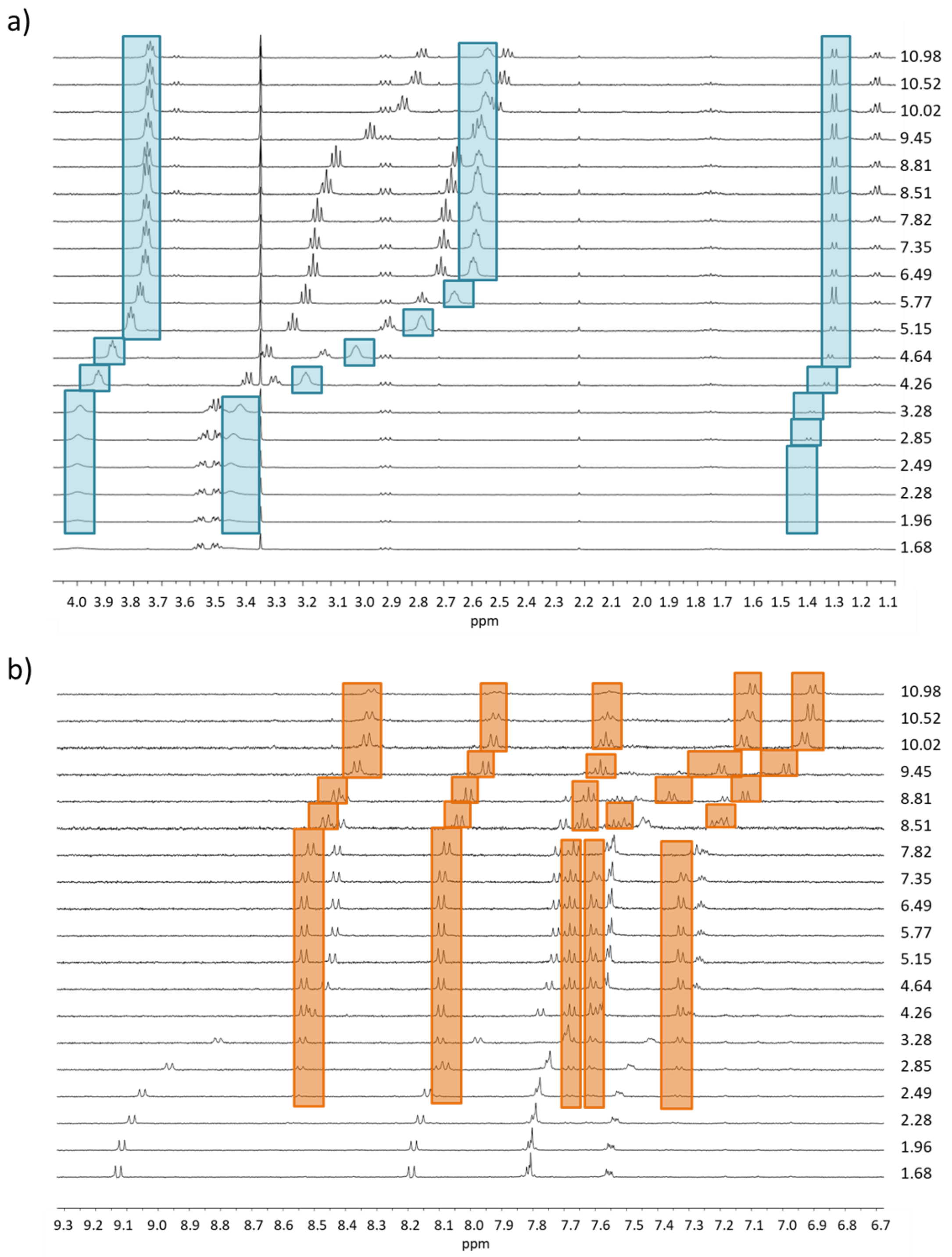

Figure S14 - ^{1}H NMR spectra of **L2** at various pH values in the a) up-field and b) the downfield regions. The framed peak assignments refer to the decomposed compound. *[c_L = 415 μM; t = 25 °C; I = 0.10 M (KCl), 10% (v/v) D_2O/H_2O]*

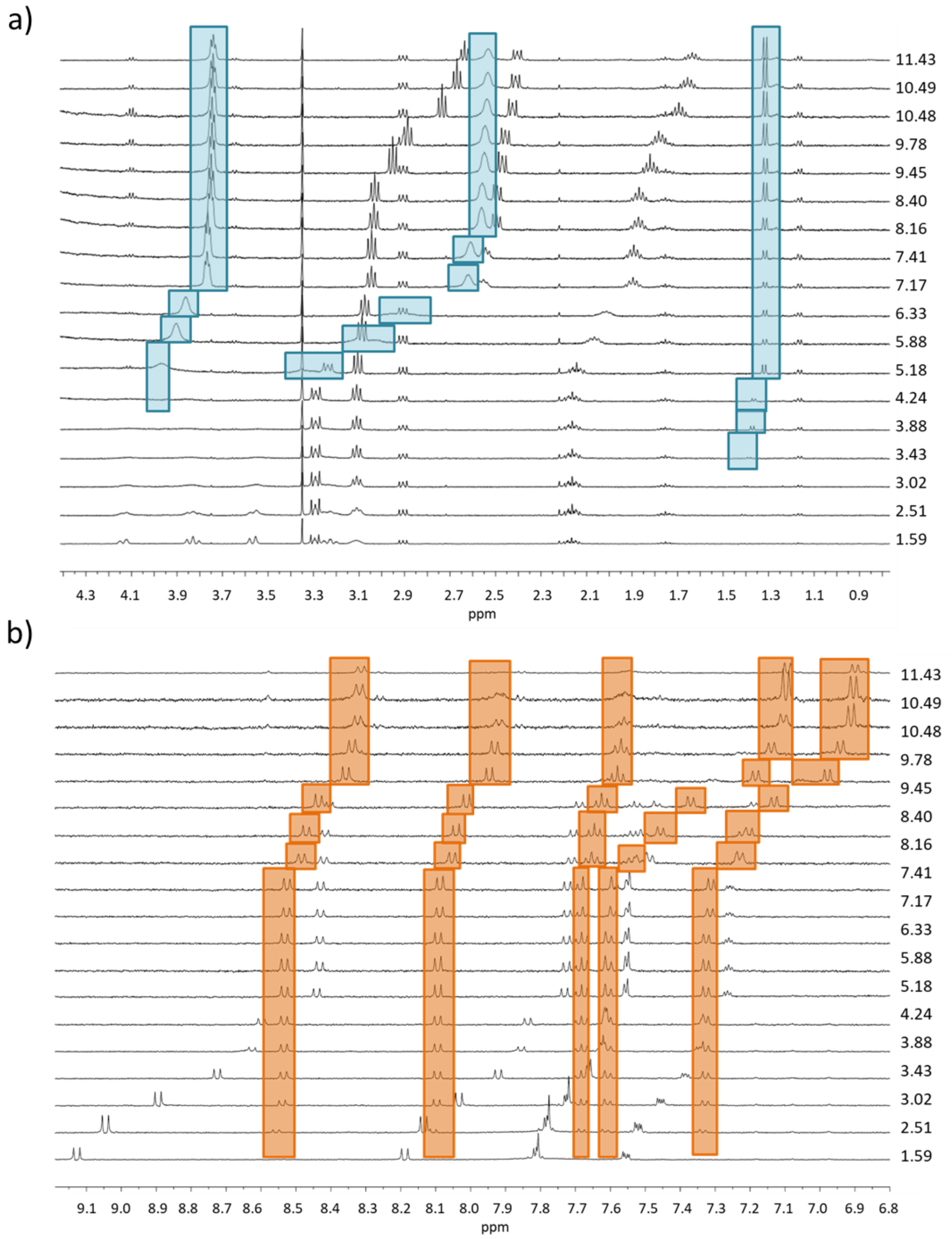

Figure S15 - ^{1}H NMR spectra of **L3** at various pH values in the a) up-field and b) the downfield regions. The framed peak assignments refer to the decomposed compound. *[c_L = 415 μM; t = 25 °C; I = 0.10 M (KCl), 10% (v/v) D_2O/H_2O]*

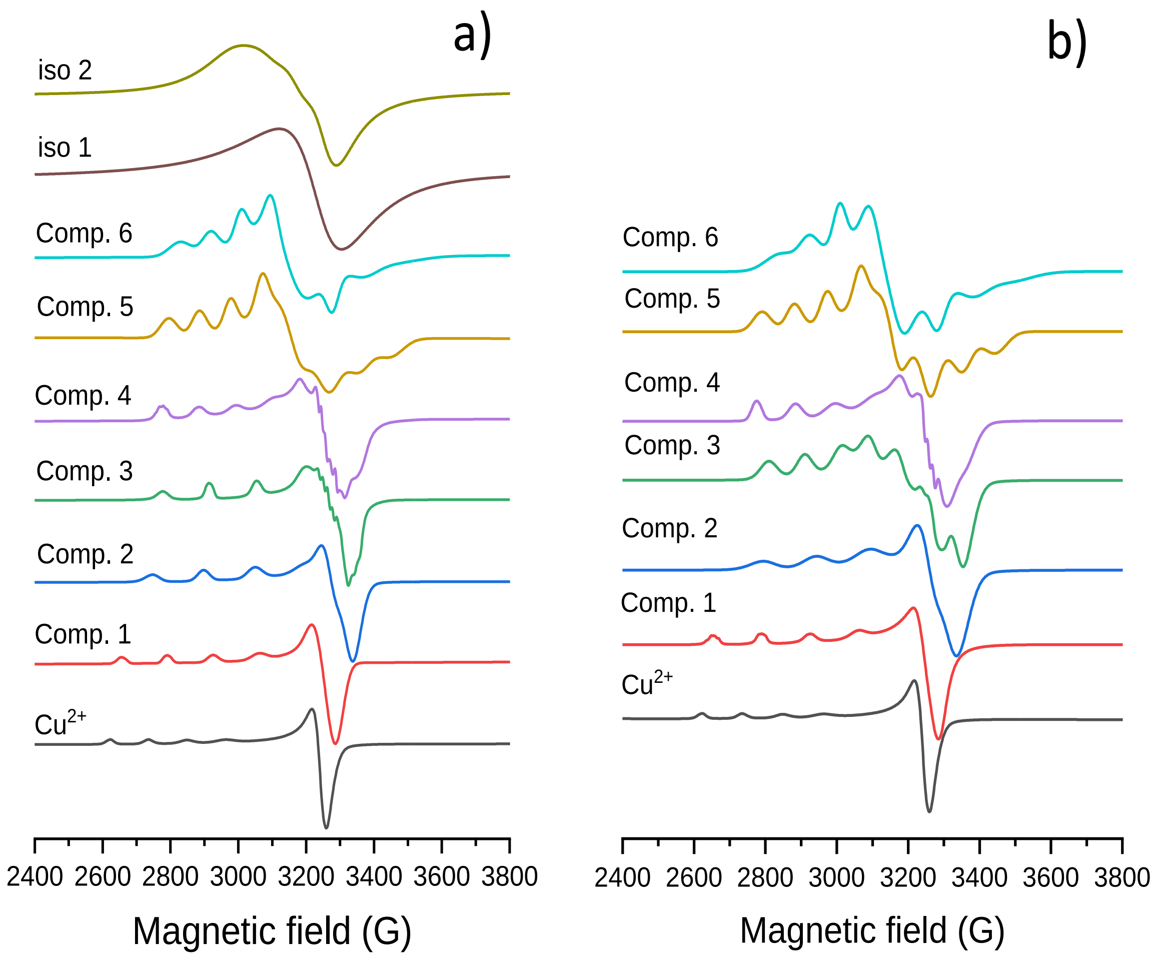

Figure S16 - Component EPR spectra obtained from the simulation of frozen solution EPR spectra recorded in the a) Cu(II) – **L2** and b) Cu(II) – **L3** systems.

Table S5 - Anisotropic EPR parameters of the complexes determined by the simulation of frozen solution EPR spectra recorded in Cu(II) – **L2** and Cu(II) – **L3** systems. Coupling values are listed in $10^{-4}cm^{-1}$ units.[a] Detailed explanation for the difficulties of the decomposition of the spectra are shown below the table. Description of the components is also shown below.

	g_x	g_y	g_z	A_x	A_y	A_z	a_x^{N1} / a_x^{N2}	a_y^{N1} / a_y^{N2}	a_z^{N1} / a_z^{N2}	$g_{o,calc}$[b]
**Cu(II) – L2**[c]										
$[Cu(aqua)]^{2+}$	2.076	2.076	2.410	8	8	126				2.187
Component 1	2.068	2.068	2.356	11	11	145				2.164
Component 2	2.062	2.062	2.263	34	34	151				2.129
Component 3	2.046	2.065	2.256	26	18	142	18 / 6	12 / 15	4 / 9	2.122
Component 4	2.052	2.055	2.292	47	17	113	13 / 6	9 / 15	4 / 12	2.133
Component 5	2.295	2.110	2.035	97	20	86				2.147
Component 6	2.270	2.135	2.027	95	10	87				2.144
**Cu(II) – L3**										
Component 1	2.068	2.068	2.356	10	10	145				2.164
Component 2	2.074	2.074	2.231	42	42	144				2.126
Component 3	2.055	2.085	2.274	25	80	106	6 / 11	14 / 9	9 / 6	2.138
Component 4	2.051	2.058	2.290	52	14	114	12 / 8	9 / 14	6 / 4	2.133
Component 5	2.295	2.110	2.035	97	20	85				2.147
Component 6	2.265	2.143	2.021	86	36	94				2.143

[a]The experimental error were ±0.002 for g_x and g_y and ±0.001 for g_z, ± 2×10^{-4} cm^{-1} for A_x and A_y and ±1 $\times10^{-4}$ cm^{-1} for A_z. [b]Calculated by the equation $g_{0,calc} = (g_x+g_y+g_z)/3$. [c]The two broad singlet components (iso 1 and iso 2) were treated with isotropic parameters $g_0 = 2.077$, $\alpha = 205$, $\beta = 36$, $\gamma = -11$ for iso 1 and $g_0 = 2.146$, $A_0 = 73$, $\alpha = 99$, $\beta = -16$, $\gamma = 4$ for iso 2.

Note: The measured spectral series in both Cu(II) – **L2** and Cu(II) – **L3** systems are quite complex, and the presence of dominant complexes could not be detected. The evaluation software can only take three component spectra into account to describe the measured spectra, which caused a significant limitation in the spectrum evaluation process. The evaluation was also further complicated by the fact that the intensity of the spectra decreased significantly in the more alkaline pH ranges, presumably due to the formation of EPR-inactive complexes and/or precipitates. The flexibility of the component spectra is also high, as four of the six components were treated with highly rhombic *g*-tensors. Based on these, the uncertainty of the determined component spectra is higher, especially in the case of minor components. Nevertheless, it was possible to fit the spectra of the two Cu(II) – ligand systems with the same six

components (with the exception of **component 3**, where the parameters differ more), which supports the reliability of the obtained component spectra.

Description of the components: **Component 1** and **component 2** were treated with axial *g*-tensors and low ligand strength and the usual elongated octahedral geometry are seen in the spectra. In regular situations, when the geometry is elongated octahedral, square pyramidal or square planar, the ground state is the d_{x2-y2} orbital and the relation $g_z > g_y \approx g_x > 2.0023$ is expected (normal spectrum). **Component 1** appears in the acidic pH range only and based on the weak ligand field strength, the coordination of one nitrogen donor is probable in this complex. **Component 2** appears between pH 5–10 and most probably it is the mono-complex with [N,O^-] coordination of the ligands. The *g*-tensor symmetry of **component 3** was found to be rhombic in both the Cu – **L2** and Cu – **L3** systems. The nitrogen splitting is resolved in the perpendicular region of these spectra and could be fitted with two nitrogen couplings. The rhombicity also suggests that probably the ligand binds to the Cu(II) ion with three donor atoms [O,N,N], which represents a significant distortion in the equatorial plane due to the coupled five-membered chelate rings. In the largest amount, this **component 3** complex is formed in the equimolar solution in the pH range of 8 – 12. At very high pH the **component 4** was detected where the A_z values decreased, and A_x value increased significantly when comparing to **component 3**. This is probably a mixed hydroxido complex that formed by the deprotonation of the water molecule coordinating in the fourth equatorial position. This complex appears at pH > 11 both in the equimolar solutions and when having ligand excess.

Component 5 and **6** only appear in solutions containing ligand excess so these are most probably bis-ligand complexes. **Component 5** forms in the acidic pH range and **component 6** in the alkaline pH range at pH > 8. For both **components 5** and **6** strongly rhombic *g*-tensor was detected which reflect that the ground state of the unpaired electron is a linear combination of the d_{x2-y2} and $d_z{}^2$ orbitals. Distortion parameter (*R*) (see explanation in section entitled 'EPR measurements of the isolated Cu(II) complexes') for **component 5** the *R* values are 0.41 for both **L2** and **L3**, and for **component 6** these values are 0.80 and 1.00 for **L2** and **L3**, respectively. This means that for **component 5** a greater contribution arises from the d_{x2-y2} orbital and the contribution of the two orbitals is approximately equal

for **component 6**. This suggests a compressed octahedral or strongly distorted octahedral geometry for these complexes which was also detected for their Zn(II) complexes from single crystal X-ray diffraction data.

In case of Cu – **L2** system broad singlet lines were considered with components iso 1 and iso 2 (**Fig. S16**). As the single crystal study of Cu – **L1** and Cu – **L2** bis-ligand complexes revealed the dinuclear nature of the complexes, it is very probable that the formation of this Cu_2L_2 may be responsible for this broad spectrum and the decrease in intensity.

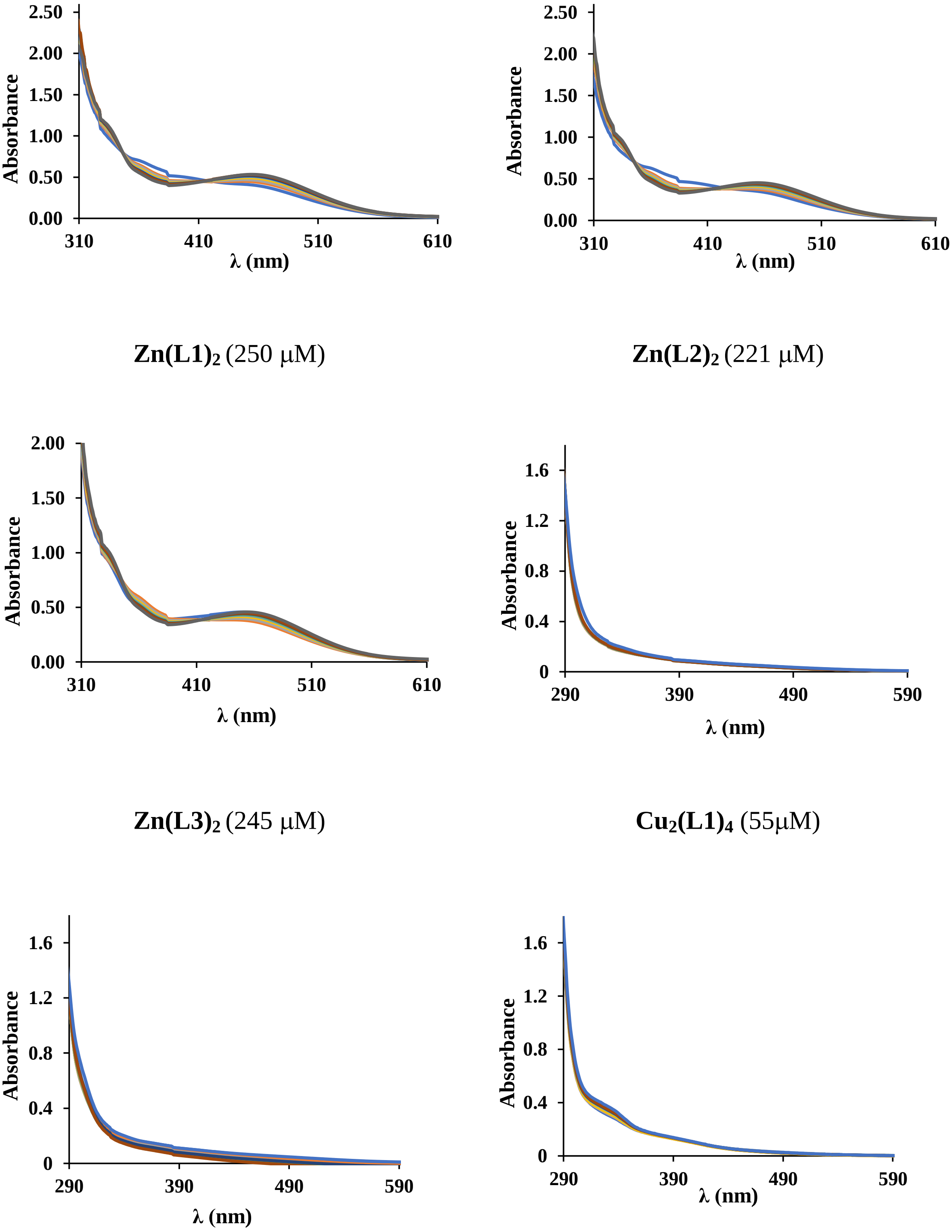

Zn(L1)$_2$ (250 μM)

Zn(L2)$_2$ (221 μM)

Zn(L3)$_2$ (245 μM)

Cu$_2$(L1)$_4$ (55μM)

Cu$_2$(L2)$_4$ (35 μM)

Cu(L3)$_2$ (45 μM)

Figure S17 – UV-vis spectra of equimolar mixtures of BSA and the Zn(II) and Cu(II) complexes in HEPES buffer: DMSO 5% (v/v), from time zero to the 24 h measurement. Concentrations are indicated for each figure.

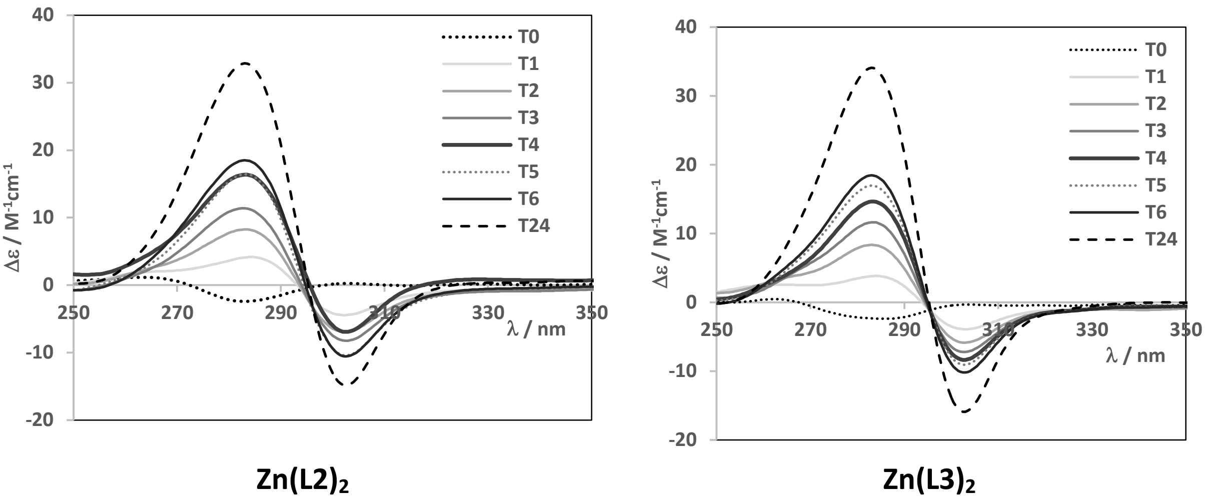

Figure S18 – Circular dichroism spectra of solutions containing BSA (20 mM) and **Zn(L2)$_2$** or **Zn(L3)$_2$** (1:1) measured with time (indicated in hours in the legend).

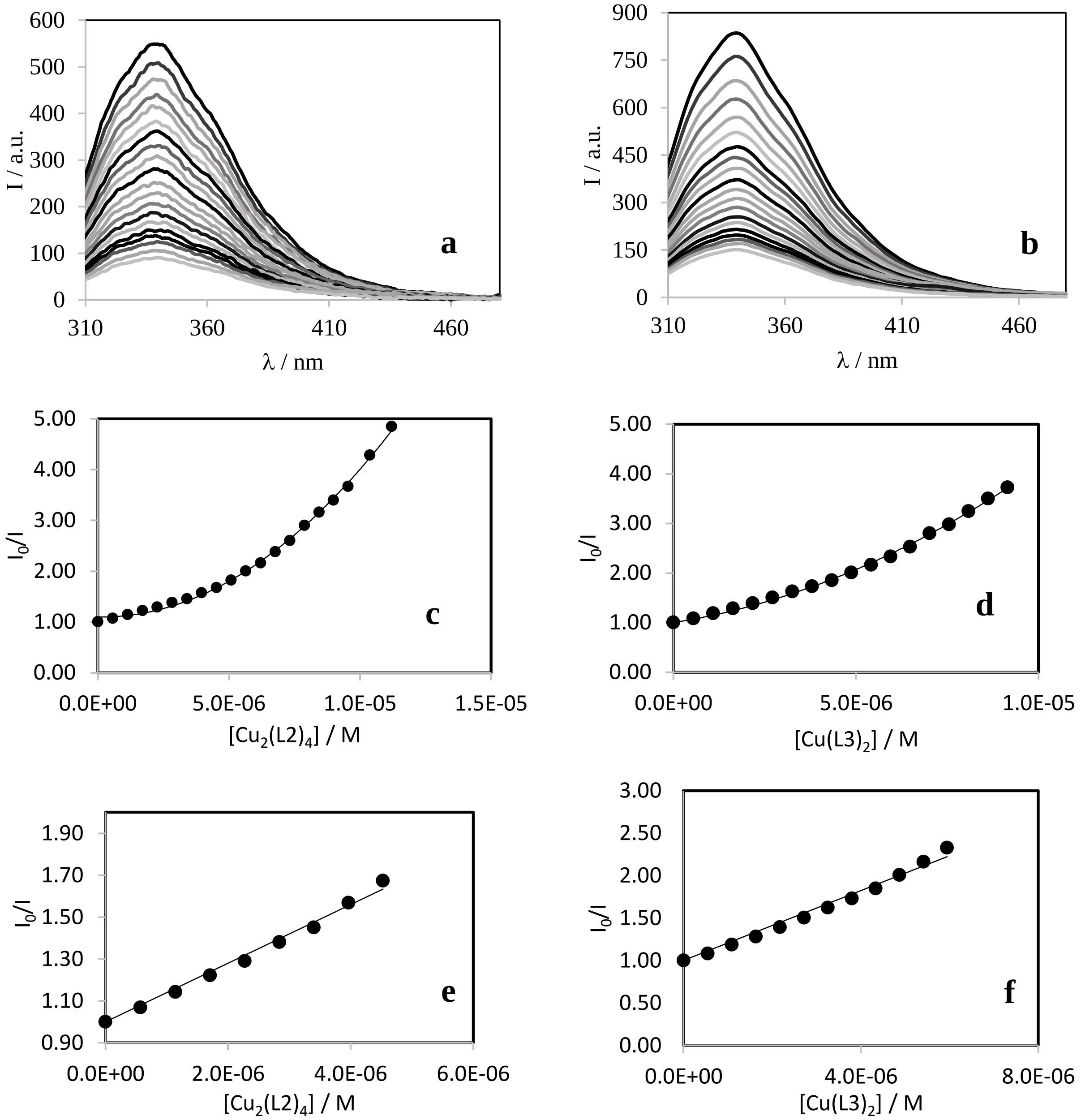

Figure S19 – Fluorescence emission spectra (λ_{exc} = 295 nm) of **a**) BSA (1.0 μM) with **$Cu_2(L2)_4$** and **b**) BSA (0.5 μM) with **$Cu(L3)_2$**. **c)** and **d)** Fitting of I_0/I (λ_{em} = 340 nm) *vs.* complex concentration with a second order equation; **c**) **$Cu_2(L2)_4$** [$I_0/I = 30\ x^2 - 1.1\times10^5\ x + 1.1$, $R^2 = 0.998$] and **d**) **$Cu(L3)_2$** [$I_0/I = 20\ x^2 + 1.2\times10^5\ x + 1$, $R^2 = 0.999$]. **e)** and **f)**: Fitting of I_0/I (λ_{em} = 340 nm) *vs* complex concentration according to the stern-Volmer equation for **e**) **$Cu_2(L2)_4$** [$I_0/I = 1.5\times10^5\ x + 1$, $R^2 = 0.993$ and f) **$Cu(L3)_2$** [$I_0/I = 2.1\times10^5\ x + 1$, $R^2 = 0.991$].

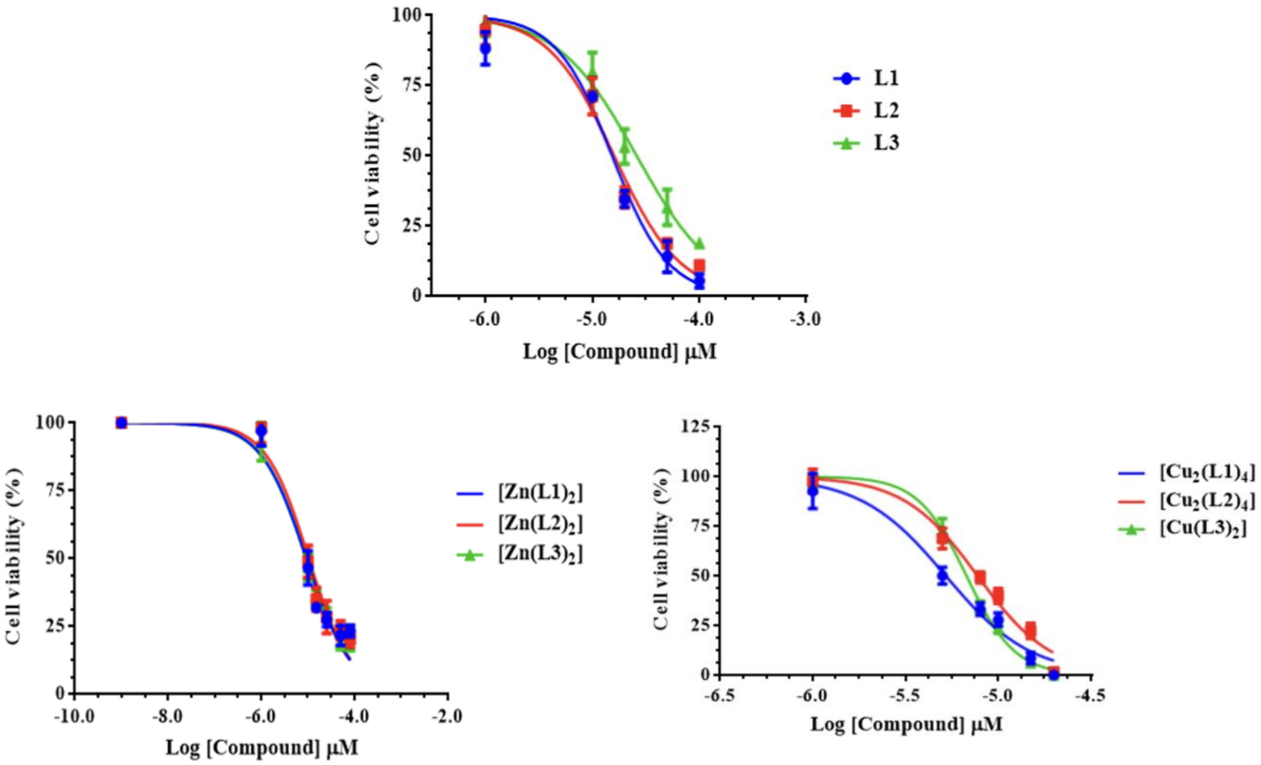

Figure S20 - Dose-response curves of the free ligands and complexes in A375 cell line after 48 h incubation.

Table S6 - *In vitro* antiproliferative activity of compounds **L1-L3**, Zn(II) complexes **Zn(L1)$_2$ -Zn(L3)$_2$** and Cu(II) complexes **Cu$_2$(L1)$_4$**, **Cu$_2$(L2)$_4$** and **Cu(L3)$_2$** after 48 h incubation in the non-tumorigenic cell line HaCaT determined by the MTT colorimetric method. Values are mean ± SD of 2 independent experiments. Selectivity index (SI) of the complexes in A375 cells compared to HaCaT (IC_{50} HaCaT/IC_{50} A375) is also presented.

**Compounds**	**IC_{50} (μM) ± SD**	**SI**
**L1**	>30	-
**[Zn(L1)$_2$]**	22.3 ± 0.7	2.5
**[Cu$_2$(L1)$_4$]**	10.3 ± 1.1	2.0
**L2**	>30	-
**[Zn(L2)$_2$]**	24.4 ± 0.6	2.4
**[Cu$_2$(L2)$_4$]**	11.8 ± 1.6	1.5
**L3**	>30	-
**[Zn(L3)$_2$]**	26.3 ± 0.7	2.8
**[Cu(L3)$_2$]**	8.1 ± 0.2	1.2
